# How Positive Psychology Can Augment Leadership Through the Therapeutic Alliance

**DOI:** 10.15766/mep_2374-8265.11510

**Published:** 2025-03-20

**Authors:** Ashten Duncan, Michael McKinney

**Affiliations:** 1 Core Faculty, Medicos de El Centro Family Medicine Residency; 2 Attending Physician, Family Medicine, CHRISTUS St. Vincent

**Keywords:** Behavior Change, Hope Theory, Interpersonal Leadership, Positive Psychology, Therapeutic Alliance, Leadership Development/Skills, Patient Care, Psychology & Behavioral Science, Well-Being/Mental Health

## Abstract

**Introduction:**

Today's health care professionals must apply psychology and leadership principles to help patients achieve health behavior changes. However, this content is not currently emphasized in most medical curricula. This curriculum synthesized these topics in a way enabling learners to apply them to direct patient care.

**Methods:**

The curriculum consisted of two 1-hour workshops about positive psychology and leadership in direct patient care, respectively. In total, 35 medical students, faculty, family medicine residents, and psychiatry residents participated. Participants completed preparticipation, postparticipation, and 6-week follow-up surveys. The primary outcomes were learners’ perceived importance, confidence, and knowledge regarding positive psychology and leadership. The secondary outcome was the impact on patient care practices after receiving the education.

**Results:**

Perceived importance of positive psychology and leadership to patient care increased modestly from pre- (*Mdn* = 4.2) to posttest (*Mdn* = 4.7). Confidence in the core concepts increased from pre- (*Mdn* = 1.6) to posttest (*Mdn* = 4.0). Knowledge increased markedly from pre- (*Mdn* = 2.9) to posttest (*Mdn* = 4.7). Participants reported changes in patient care practices after receiving the positive psychology and leadership content at the 6-week follow-up. They also reported high levels of relevance of the content to direct patient care.

**Discussion:**

This curriculum significantly increased learners’ perceived importance, confidence, and knowledge regarding the core topics and was associated with changes in their patient care practices. Given its brevity and effectiveness at producing participant-level behavior changes, this content could be easily integrated into medical trainee and staff didactic time.

## Educational Objectives

By the end of this session, learners will be able to:

1. List major positive psychology frameworks that have emerged in recent years and articulate the importance and relevance of these frameworks to direct patient care.
2. Summarize the basic neurobiology of key positive psychology traits.
3. Classify the components of hope to nurturing well-being and relate this to situations in which patients may be deficient in hope.
4. Describe how goal prioritization can promote better health and apply this concept to barriers to health-related goal attainment.
5. Summarize key interpersonal leadership concepts and cognitive biases that influence the patient-provider dyad and articulate the importance and relevance of these topics to direct patient care.
6. Describe how emotional intelligence, self-awareness, and psychological safety impact patient care and use this knowledge to navigate common patient care challenges.
7. Identify the role of the physician/provider as a leader in the therapeutic alliance and apply this understanding to cases involving patient care.

## Introduction

For several decades, the majority of diseases, disability, and deaths in the United States have been attributable to chronic diseases that are directly linked to preventable lifestyle factors.^1^ Reflecting this reality, the United States Department of Health and Human Services has outlined several leading health indicators tied to modifiable health behaviors in Healthy People 2030.^2^ As a result of this health paradigm shift, modern health care requires physicians and other health care professionals who are adept at applying psychology and leadership principles to direct patient care to help patients achieve health behavior changes and other health-related goals.^3–5^ However, this content is not currently emphasized in many undergraduate and graduate medical curricula. Consequently, physicians outside of behavioral health care settings do not routinely apply these concepts to their care of patients.^6^ There needs to be clear, standardized content to educate medical trainees, including medical students and resident physicians.

In the late 20th century, positive psychology emerged as a field that distinguished itself from the rest of psychology and psychiatry by focusing on the cognitive, affective, and behavioral dimensions of optimal human functioning, which refers to people being able to achieve their goals and potentials in the absence of adversity.^7^ Major psychological states and traits (i.e., temporary conditions and long-term characteristics, respectively) include frameworks like hope,^8^ flow,^9^ and many others connected to psychosocial flourishing, which occurs when people can focus on their purposes in life rather than individual challenges.^10^ As a specific example, hope theory describes how pathways thinking (i.e., mental road maps to goal attainment) and agency thinking (i.e., mental energy to use a specific pathway) influence a person's tendency toward goal pursuit.

While different positive psychological states and traits translate to different behaviors (e.g., hope promoting goal pursuit, gratitude promoting positive appraisal of experiences, etc.), there is robust research showing the similar effects they have on the brain. Functional MRI and other neurobiological studies have demonstrated how the brain works differently when self-reported levels of positive psychological domains like hope are high,^11,12^ and this difference has significant implications for executive function.^13^ Specifically, these differences are reflected in increased activity in the prefrontal cortex, which is essential to goal setting and other adaptive functions.^13^ For these reasons, it has been theorized that positive psychology has a direct and critical role to play in promoting health behavior changes in patients.^3^

Most undergraduate and graduate medical curricula acknowledge how indispensable strong leadership is to high-quality patient care and teamwork, particularly in topics like motivational interviewing^14^ and bidirectional performance feedback.^15^ Underpinning this emphasis is the well-accepted theory that adequate leadership practices lead to better outcomes for an organization,^16^ which can include better health outcomes for a health care organization. More recent research on interpersonal leadership has also demonstrated how impactful skills and qualities like emotional intelligence,^17^ psychological safety,^18^ and specific adaptive leadership styles^19^ are to achieving optimal organizational performance. For these reasons, a deep understanding of leadership concepts has the potential to improve direct patient care, especially when coupled with major underlying theories of positive psychology.

Educational approaches to date have included isolated didactic content on special topics in positive psychology and leadership, as well as leadership workshops.^20,21^ While important in their coverage of these topics, those past approaches have fallen short because they have not directly shown how positive psychology can inform leadership practices and how effective leadership can radically affect the health of patients and their communities.^3,9–11^ Our work improves upon this by synthesizing core topics in positive psychology and interpersonal leadership so that learners can apply them to direct patient care and understand the foundations of optimal goal-setting environments.

## Methods

### Curricular Content and Materials

To align with the educational delivery methods most medical trainees would encounter as students and residents, we created a curriculum consisting of two interactive workshops lasting 1 hour each during dedicated didactic time and a medical education conference. These workshops occurred one after the other during a 2-hour block and were intended for medical students in their fourth year and resident physicians at any level of training. We needed only a conference room, projector, screen, and computer to implement the workshops. We found that the ideal number of learners per session ranged from 10 to 15.

For these workshops, instructors had to be experienced in clinical medicine and direct patient care to facilitate discussions among the learners about how the topics applied to the provision of health care services. Additional training in psychology and leadership was preferred but optional. We facilitated this educational programming and required basic knowledge of the core topics covered in the workshops. These topics included major positive psychology frameworks, basic neurobiology of critical positive psychology traits, goal prioritization, cognitive biases, emotional intelligence, self-awareness, and psychological safety (Appendices A and B). We followed the facilitator guide closely to ensure consistent content delivery during each session (Appendix C).

### Implementation

The first workshop concerned foundational topics and applied theory in positive psychology (Appendix A). After participants completed a preparticipation survey (Appendix D) using Google Forms, we provided a 15-minute introduction to major positive psychology frameworks, a 5-minute review of the basic neurobiology of key positive psychology traits, a 10-minute discussion of the components of hope nurturing well-being, and a 5-minute overview of how goal prioritization could promote better health. We then moved into audience engagement questions and case-based discussions for about 20 minutes. After completing the content for the first workshop, we immediately transitioned to the second one.

The second workshop centered on interpersonal leadership in direct patient care and the therapeutic alliance (Appendix B). We provided a 20-minute introduction to key interpersonal leadership concepts and cognitive biases influencing the patient-provider dyad; a 10-minute review of how emotional intelligence, self-awareness, and psychological safety could impact patient care; and a 4-minute overview of the role of the health care provider as a leader in the therapeutic alliance. We then dedicated 6 minutes to an interactive case discussion and 10 minutes to a general group discussion before concluding. Following the presentation, participants completed a postparticipation survey (Appendix D) using Google Forms.

### Participants

Thirty-five medical students, faculty, family medicine residents, and psychiatry residents participated. These participants came from the University of New Mexico—Santa Fe Family Medicine Residency Program, the University of New Mexico Psychiatry Residency Program, and Building the Next Generation of Academic Physicians (BNGAP). During the sessions, we presented informational slides with key definitions and conceptual models to ground the learners in the workshop's major takeaways. We then followed up with audience engagement questions, group discussion questions, and interactive cases requiring the application of the information covered in the presentation. We needed only basic facilitation skills to deliver the content in each workshop (Appendix C).

### Evaluation

To assess the effectiveness of the educational content, participants completed preparticipation, postparticipation, and 6-week follow-up surveys (Appendix D). We developed the surveys based on the learning objectives for the educational content and a brief review of the medical education literature, without a validation procedure. Internal reliability values for the preparticipation, postparticipation, and follow-up surveys were all acceptable, with Cronbach's alpha values of .82, .79, and .77, respectively. The primary outcomes were learners’ perceived importance, confidence, and knowledge regarding positive psychology and leadership based on our learning objectives. The secondary outcome was the impact on patient care practices after receiving the education. We selected these specific outcomes to evaluate reactions, learning, and behavior based on the first three levels of the Kirkpatrick model of learning evaluation.^22^

Given the small sample size and skewness of the distributions, we performed nonparametric mean comparison testing using the Mann-Whitney *U* test and screening group comparison testing using the Kruskal-Wallis *H* test and calculated descriptive statistics where appropriate. For the preparticipation and postparticipation surveys, the response rate was 100%. After exempt review, this project was approved by the University of New Mexico Institutional Review Board (2761125605).

## Results

We implemented the educational module three times: once in person during family medicine residents’ didactic session in February 2023, once virtually during the BNGAP Medical Education Conference in March 2023, and once virtually during psychiatry residents’ didactic session in May 2023. Of the 35 total participants, three (9%) were faculty, eight (23%) were medical students, 16 (46%) were family medicine residents, and eight (23%) were psychiatry residents. The majority of participants identified as female (63%), between the ages of 20 and 35 (83%), and White (57%). Other racial and ethnic groups represented included Black (11%), Native American (9%), Hispanic (14%), and Asian (9%). The initial analysis of variance demonstrated no significant differences in response patterns based on any other demographic characteristics.

Based on 5-point Likert-type scales (1 corresponding to a low level, 5 to a high one; Appendix D), participants’ perceived importance of positive psychology and leadership to patient care increased modestly from pre- (*Mdn* = 4.2, IQR = 4.0–4.5) to posttest (*Mdn* = 4.7, IQR = 4.4–4.9, *U* = 6.6, *p* = .01). Confidence with the core concepts increased from pre- (*Mdn* = 1.6, IQR = 1.2–2.3) to posttest (*Mdn* = 4.0, IQR = 3.5–4.5, *U* = 5.4, *p* = .02). Knowledge increased markedly from pre- (*Mdn* = 2.9, IQR = 2.4–3.5) to posttest (*Mdn* = 4.7, IQR = 4.4–4.9, *U* = 16.3, *p* < .001).

The Table summarizes all of the question-level medians, interquartile ranges, and statistical values from the preparticipation and postparticipation surveys.

**Table.**
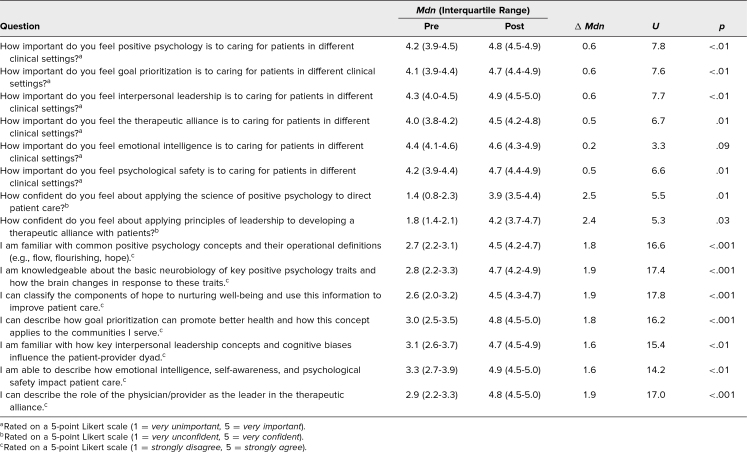
Results From Preparticipation and Postparticipation Surveys (*N* = 35)

For the follow-up survey, 26 participants (74%) responded. The survey questions used 10-point Likert-type scales (1 corresponding to low, 5 to neutral, and 10 to high; Appendix D). These scales differed from the initial 5-point scales to increase the sensitivity of our measurements since we used nonvalidated questions. Participants reported significant changes in patient care practices after receiving both the positive psychology (*M* = 7.2, *SD* = 0.7) and leadership content (*M* = 8.1, *SD* = 0.7) at the 6-week follow-up. They also reported high levels of relevance of the content to direct patient care (*M* = 7.9, *SD* = 0.9). In the optional feedback section on the follow-up survey, a few common themes emerged regarding the educational content, including how the content “helped guide conversations with patients,” “made goals of care discussions more frequent,” and “supported rapport-building with patients and their families.”

## Discussion

This curriculum centering on positive psychology and interpersonal leadership in the therapeutic alliance produced favorable results. Our results demonstrated that the curriculum significantly increased learners’ perceived importance, confidence, and knowledge regarding the core topics and was associated with changes in their patient care practices. This is promising because other researchers have highlighted practical implementation strategies for positive psychology in clinical care, making this content highly actionable.^23^

While creating the content and designing the workshops, we learned how little participants had previously heard about positive psychology and its intersections with leadership concepts. Although we did not formally collect qualitative data, we observed a positive and enthusiastic response to applying the concepts introduced in the workshops to actual patient care, which likely translated to the behavioral changes we captured. After the three implementations, the contents of the presentations were streamlined to maximize group discussion time based on immediate participant feedback. This involved condensing the text, focusing more on the figures and tables in the slide decks, and highlighting the key takeaways from information-dense slides.

This educational project has several notable strengths and limitations. Its strengths include theory-derived content, exercises to practice applying the concepts to actual patient care scenarios, short duration, multifaceted assessment strategy grounded in the Kirkpatrick model of learning evaluation, and overlap of familiar and unfamiliar concepts. Our limitations include a geographically limited and small sample, likely self-selection bias suggested by the skewness of the distributions, use of nonparametric statistical methods, use of nonvalidated survey questions, and lack of narrative data and qualitative synthesis. Regarding external validity, the results are generalizable only to medical students, primary care resident physicians, and faculty with a vested interest in these topics.

This project signals the need for further research into how positive psychology and leadership content can be more expansively integrated into undergraduate and graduate medical education, as well as into the content's potential long-term implications for patient care practices. Given its brevity and demonstrated effectiveness, this content could be easily integrated into didactic time for medical trainees and staff to enhance direct patient care. Future researchers could expand on this work by providing more instruction to learners on specific positive psychology interventions, which have been shown to be beneficial for different populations.^24^

## Appendices


Intro to Positive Psychology.pptxIntro to Leadership in the Therapeutic Alliance.pptxFacilitator Guide.docxSurveys.docx
*All appendices are peer reviewed as integral parts of the Original Publication.*
