# Supplementary material for: How Positive Psychology Can Augment Leadership Through the Therapeutic Alliance
Source: MedEdPORTAL. 2025 Mar 20;21:11510. doi: 10.15766/mep_2374-8265.11510 (PMC11922800; doi:10.15766/mep_2374-8265.11510)
Supplement: Supplementary file 1 — Intro to Positive Psychology.pptxIntro to Leadership in the Therapeutic Alliance.pptxFacilitator Guide.docxSurveys.docx [file mep_2374-8265.11510-s001.zip › B. Intro to Leadership in the Therapeutic Alliance.pptx]

## Slide 1
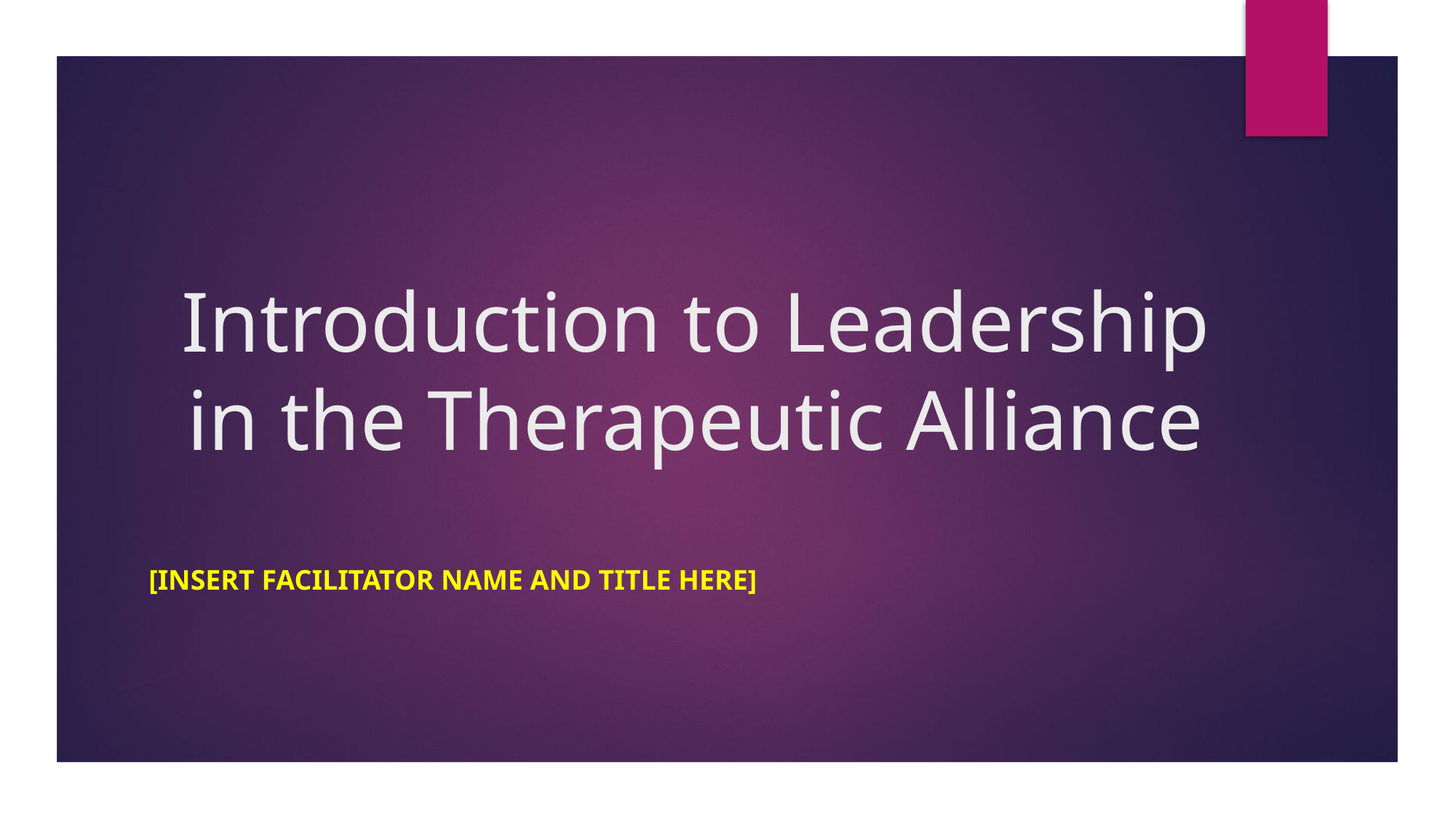

# Introduction to Leadership in the Therapeutic Alliance
[INSERT FACILITATOR NAME AND TITLE HERE]

## Slide 2
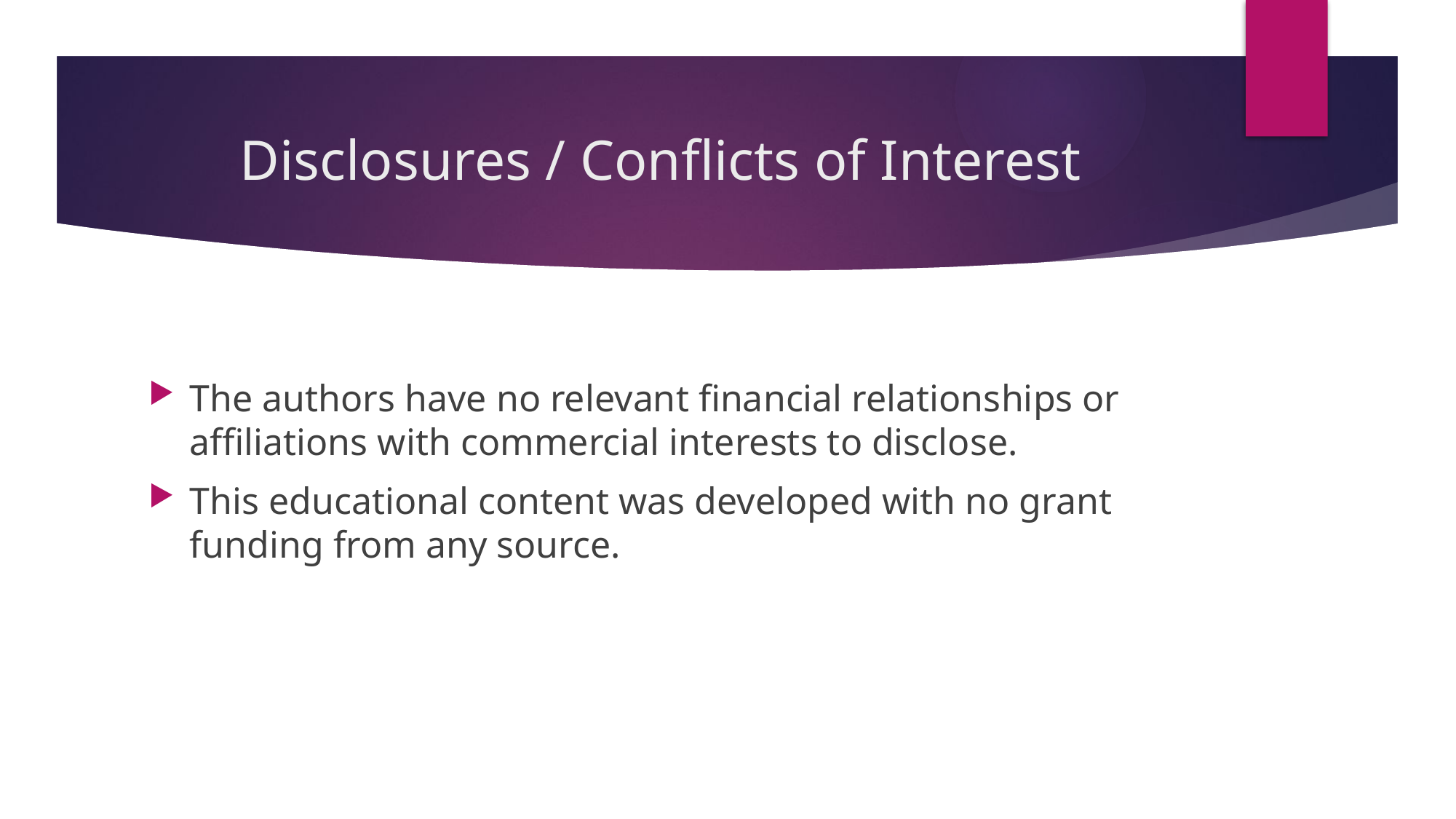

# Disclosures / Conflicts of Interest
The authors have no relevant financial relationships or affiliations with commercial interests to disclose.
This educational content was developed with no grant funding from any source.

## Slide 3
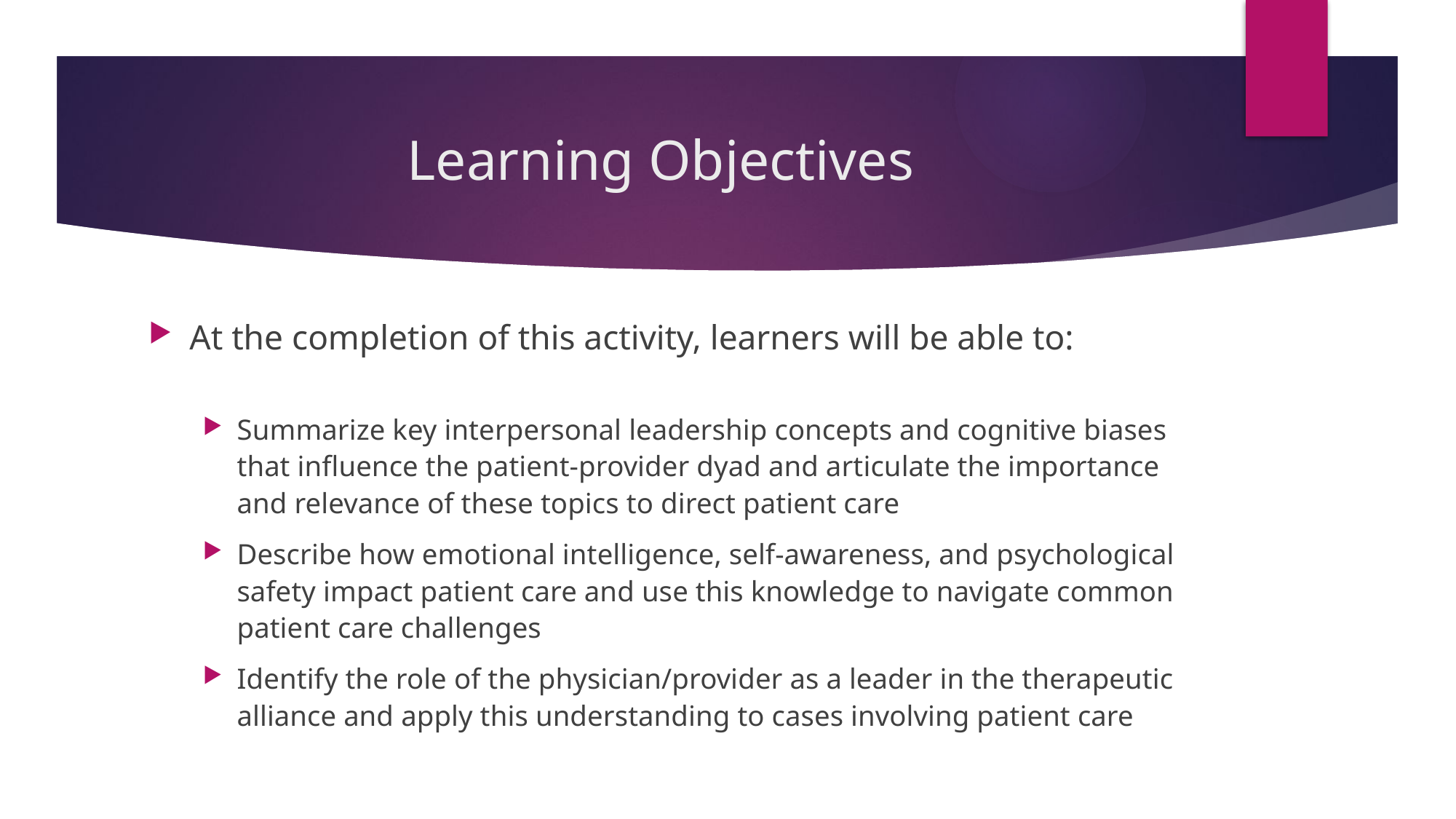

# Learning Objectives
At the completion of this activity, learners will be able to:
Summarize key interpersonal leadership concepts and cognitive biases that influence the patient-provider dyad and articulate the importance and relevance of these topics to direct patient care
Describe how emotional intelligence, self-awareness, and psychological safety impact patient care and use this knowledge to navigate common patient care challenges
Identify the role of the physician/provider as a leader in the therapeutic alliance and apply this understanding to cases involving patient care

## Slide 4
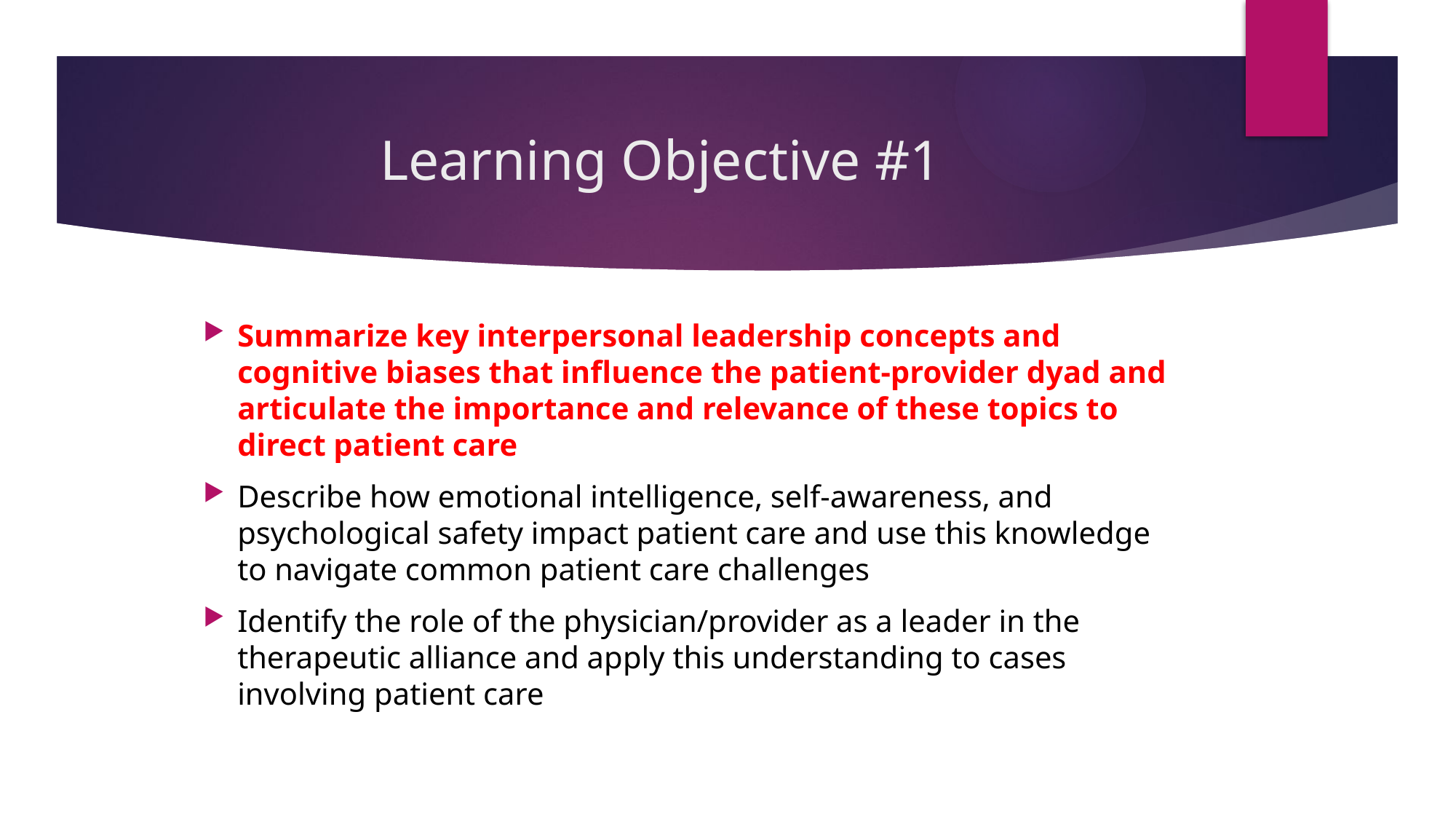

# Learning Objective #1
Summarize key interpersonal leadership concepts and cognitive biases that influence the patient-provider dyad and articulate the importance and relevance of these topics to direct patient care
Describe how emotional intelligence, self-awareness, and psychological safety impact patient care and use this knowledge to navigate common patient care challenges
Identify the role of the physician/provider as a leader in the therapeutic alliance and apply this understanding to cases involving patient care

## Slide 5
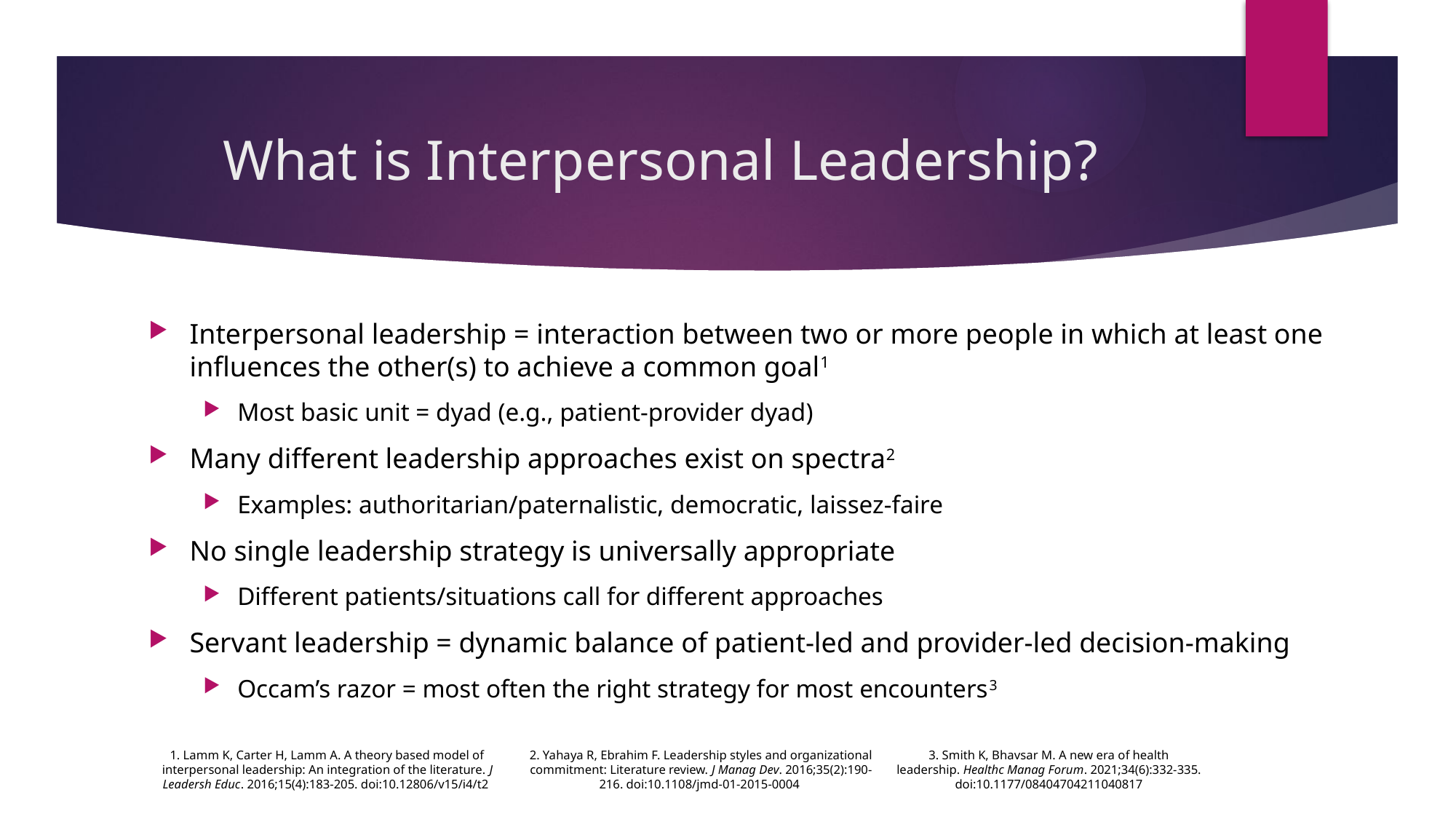

# What is Interpersonal Leadership?
Interpersonal leadership = interaction between two or more people in which at least one influences the other(s) to achieve a common goal1
Most basic unit = dyad (e.g., patient-provider dyad)
Many different leadership approaches exist on spectra2
Examples: authoritarian/paternalistic, democratic, laissez-faire
No single leadership strategy is universally appropriate
Different patients/situations call for different approaches
Servant leadership = dynamic balance of patient-led and provider-led decision-making
Occam’s razor = most often the right strategy for most encounters3
3. Smith K, Bhavsar M. A new era of health leadership. Healthc Manag Forum. 2021;34(6):332-335. doi:10.1177/08404704211040817
2. Yahaya R, Ebrahim F. Leadership styles and organizational commitment: Literature review. J Manag Dev. 2016;35(2):190-216. doi:10.1108/jmd-01-2015-0004
1. Lamm K, Carter H, Lamm A. A theory based model of interpersonal leadership: An integration of the literature. J Leadersh Educ. 2016;15(4):183-205. doi:10.12806/v15/i4/t2

## Slide 6
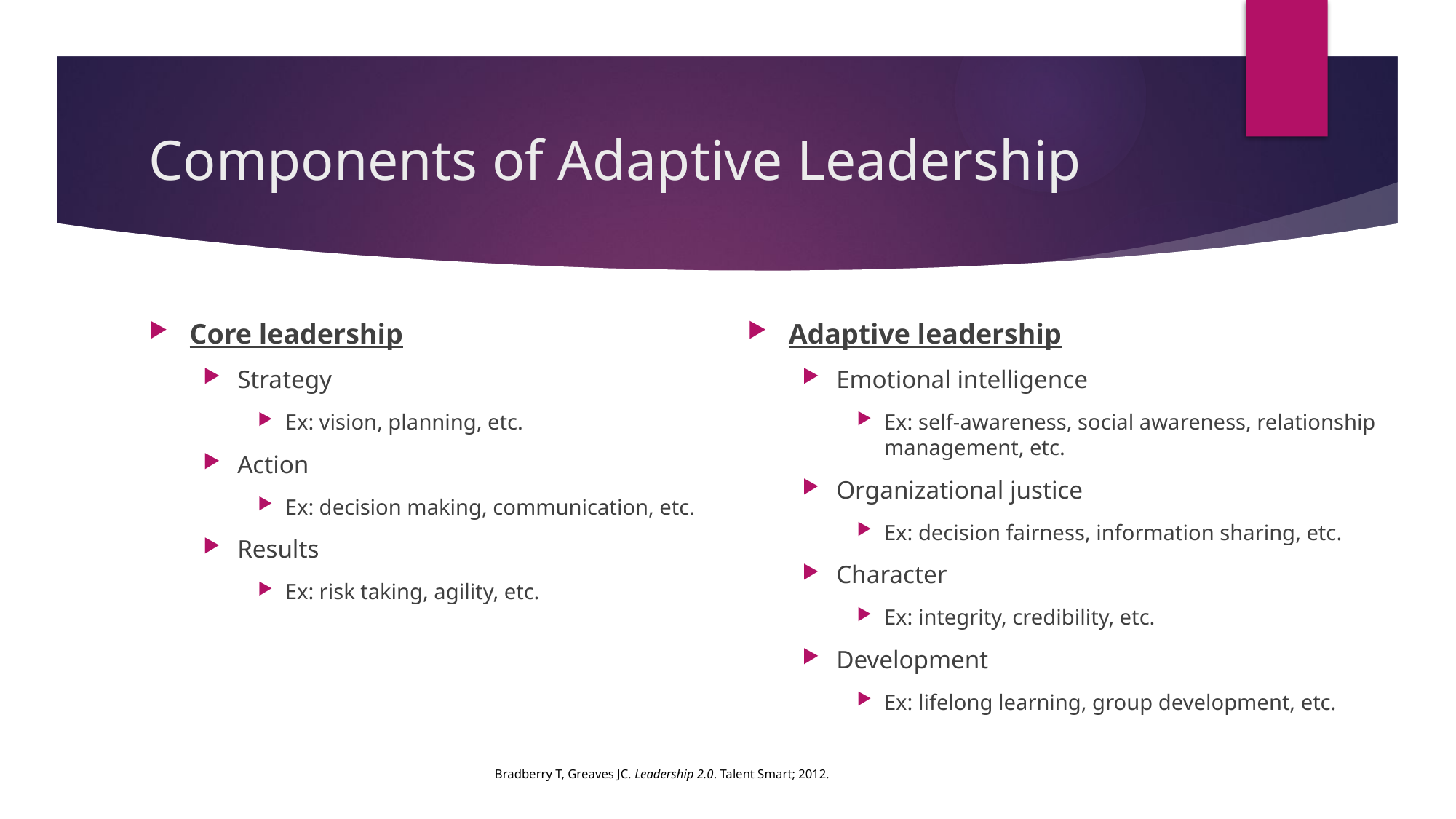

# Components of Adaptive Leadership
Core leadership
Strategy
Ex: vision, planning, etc.
Action
Ex: decision making, communication, etc.
Results
Ex: risk taking, agility, etc.
Adaptive leadership
Emotional intelligence
Ex: self-awareness, social awareness, relationship management, etc.
Organizational justice
Ex: decision fairness, information sharing, etc.
Character
Ex: integrity, credibility, etc.
Development
Ex: lifelong learning, group development, etc.
Bradberry T, Greaves JC. Leadership 2.0. Talent Smart; 2012.

## Slide 7
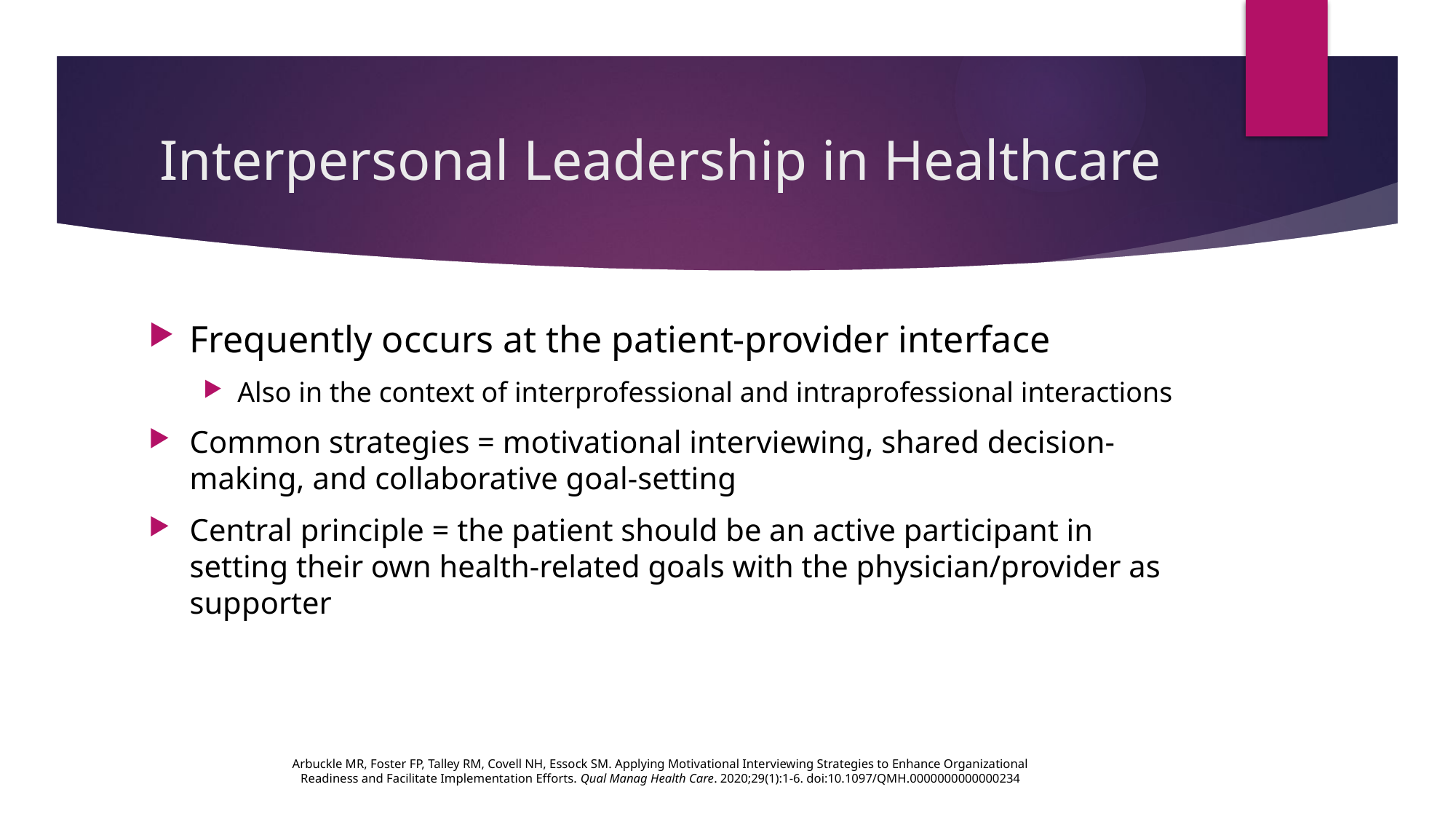

# Interpersonal Leadership in Healthcare
Frequently occurs at the patient-provider interface
Also in the context of interprofessional and intraprofessional interactions
Common strategies = motivational interviewing, shared decision-making, and collaborative goal-setting
Central principle = the patient should be an active participant in setting their own health-related goals with the physician/provider as supporter
Arbuckle MR, Foster FP, Talley RM, Covell NH, Essock SM. Applying Motivational Interviewing Strategies to Enhance Organizational Readiness and Facilitate Implementation Efforts. Qual Manag Health Care. 2020;29(1):1-6. doi:10.1097/QMH.0000000000000234

## Slide 8
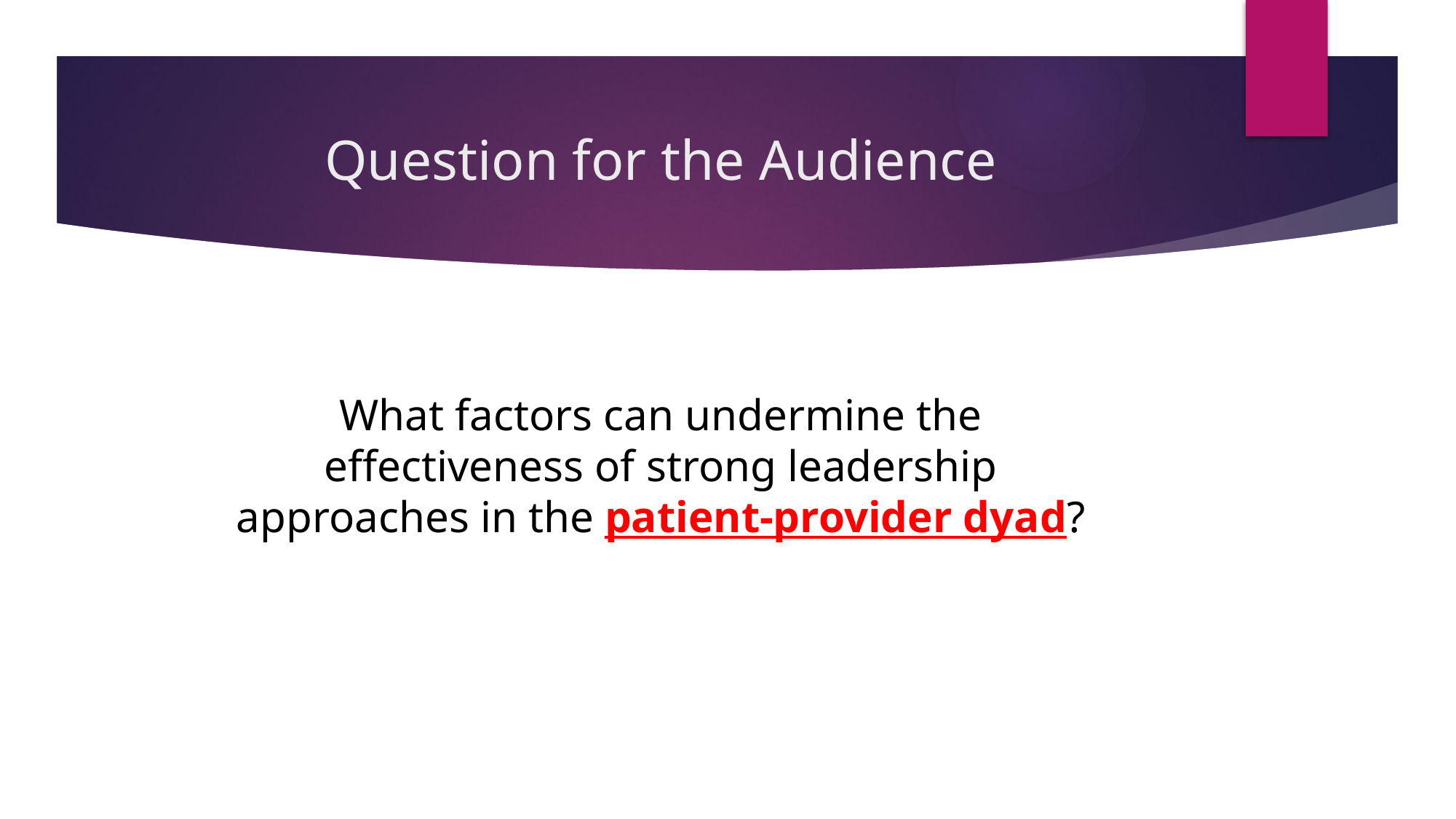

# Question for the Audience
What factors can undermine the effectiveness of strong leadership approaches in the patient-provider dyad?

## Slide 9
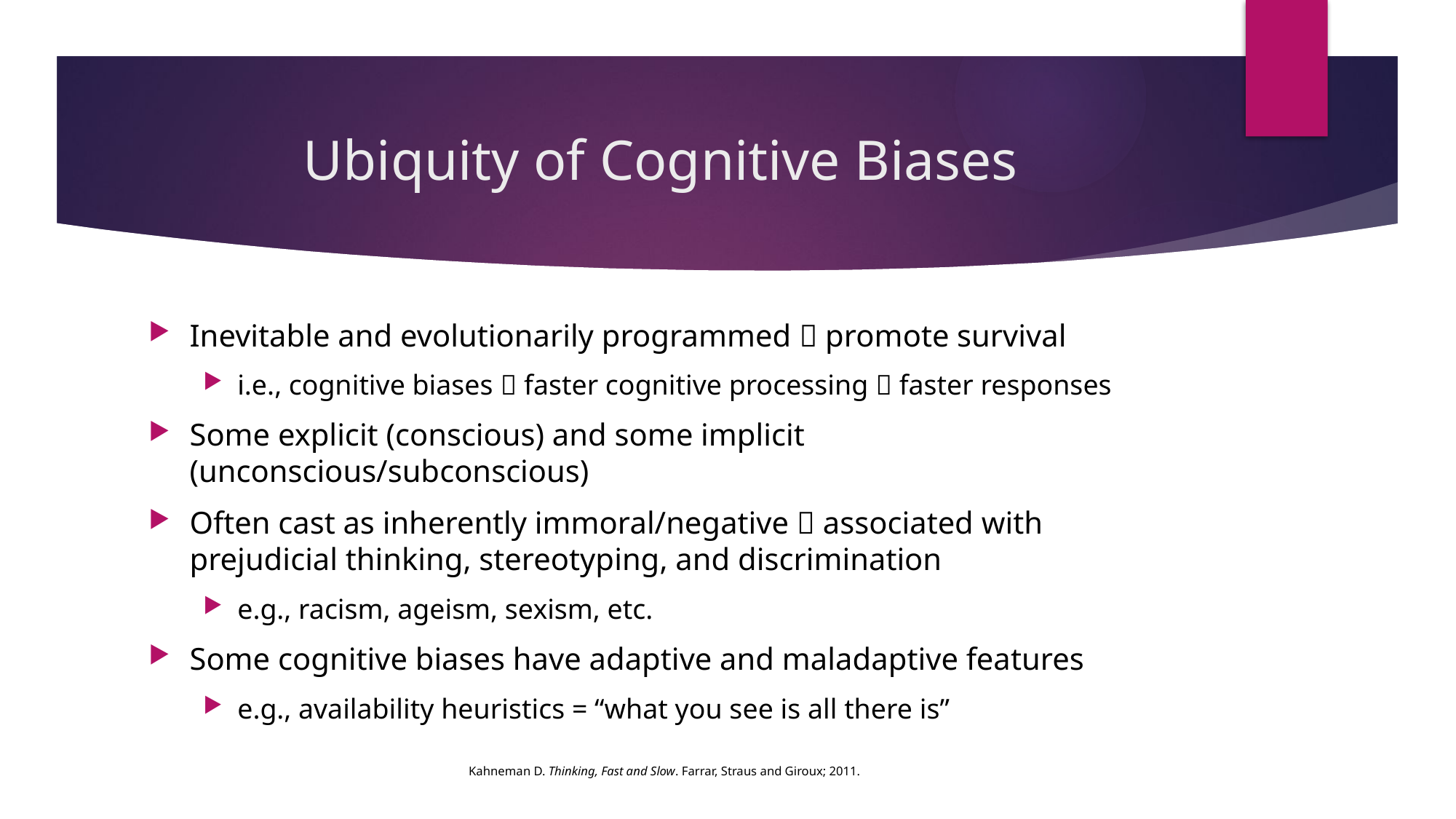

# Ubiquity of Cognitive Biases
Inevitable and evolutionarily programmed  promote survival
i.e., cognitive biases  faster cognitive processing  faster responses
Some explicit (conscious) and some implicit (unconscious/subconscious)
Often cast as inherently immoral/negative  associated with prejudicial thinking, stereotyping, and discrimination
e.g., racism, ageism, sexism, etc.
Some cognitive biases have adaptive and maladaptive features
e.g., availability heuristics = “what you see is all there is”
Kahneman D. Thinking, Fast and Slow. Farrar, Straus and Giroux; 2011.

## Slide 10
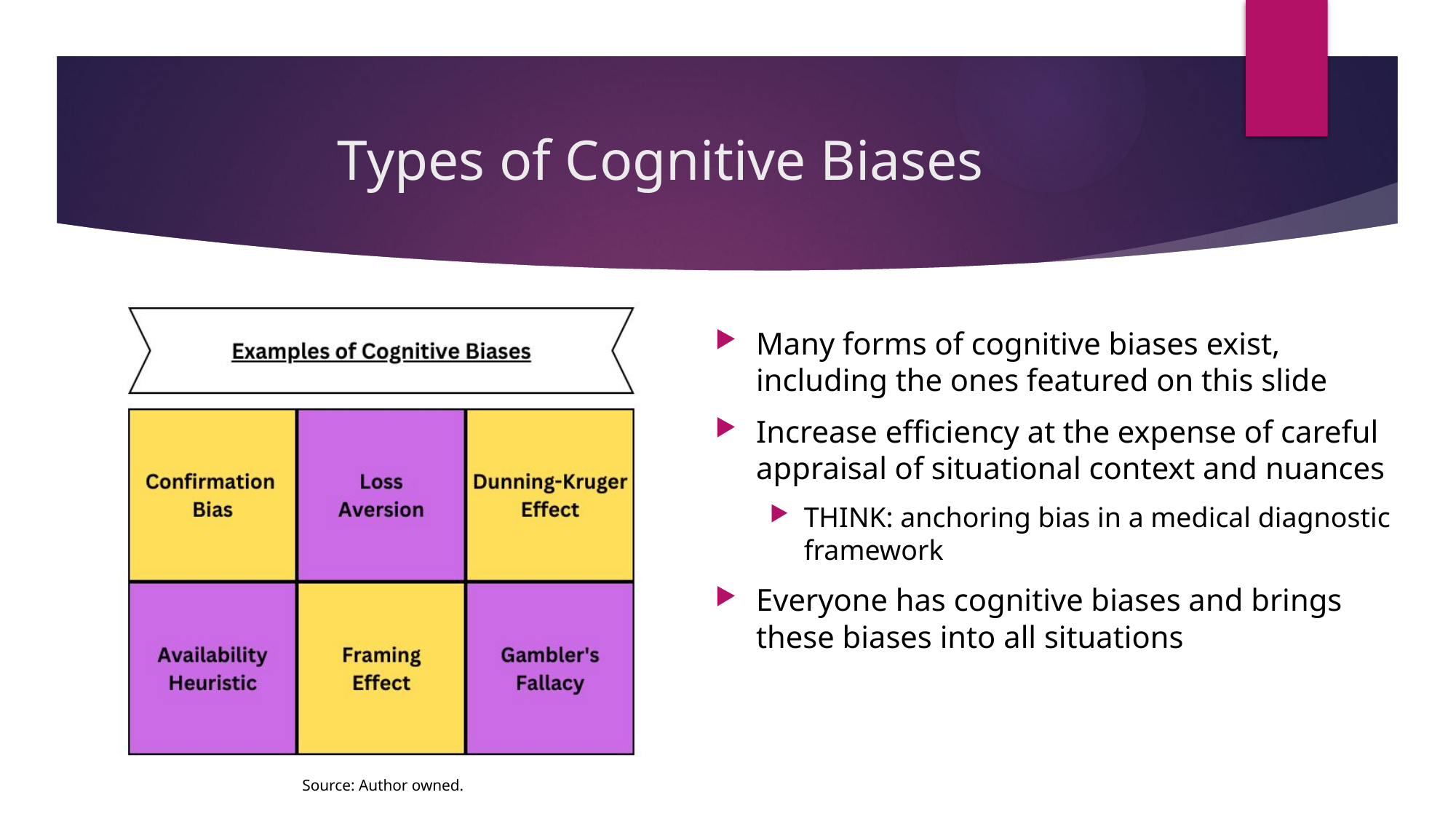

# Types of Cognitive Biases
Many forms of cognitive biases exist, including the ones featured on this slide
Increase efficiency at the expense of careful appraisal of situational context and nuances
THINK: anchoring bias in a medical diagnostic framework
Everyone has cognitive biases and brings these biases into all situations
Source: Author owned.

## Slide 11
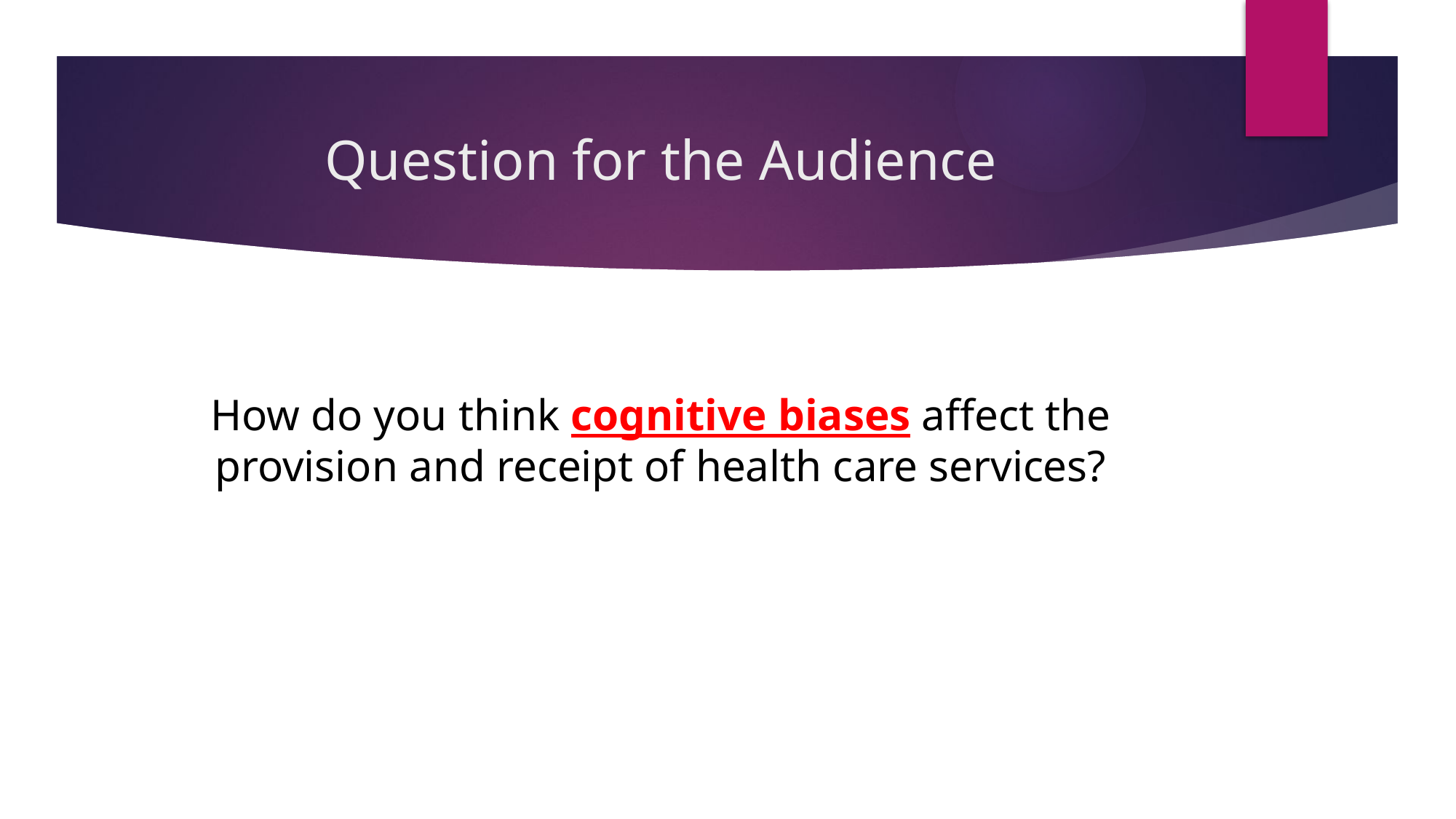

# Question for the Audience
How do you think cognitive biases affect the provision and receipt of health care services?

## Slide 12
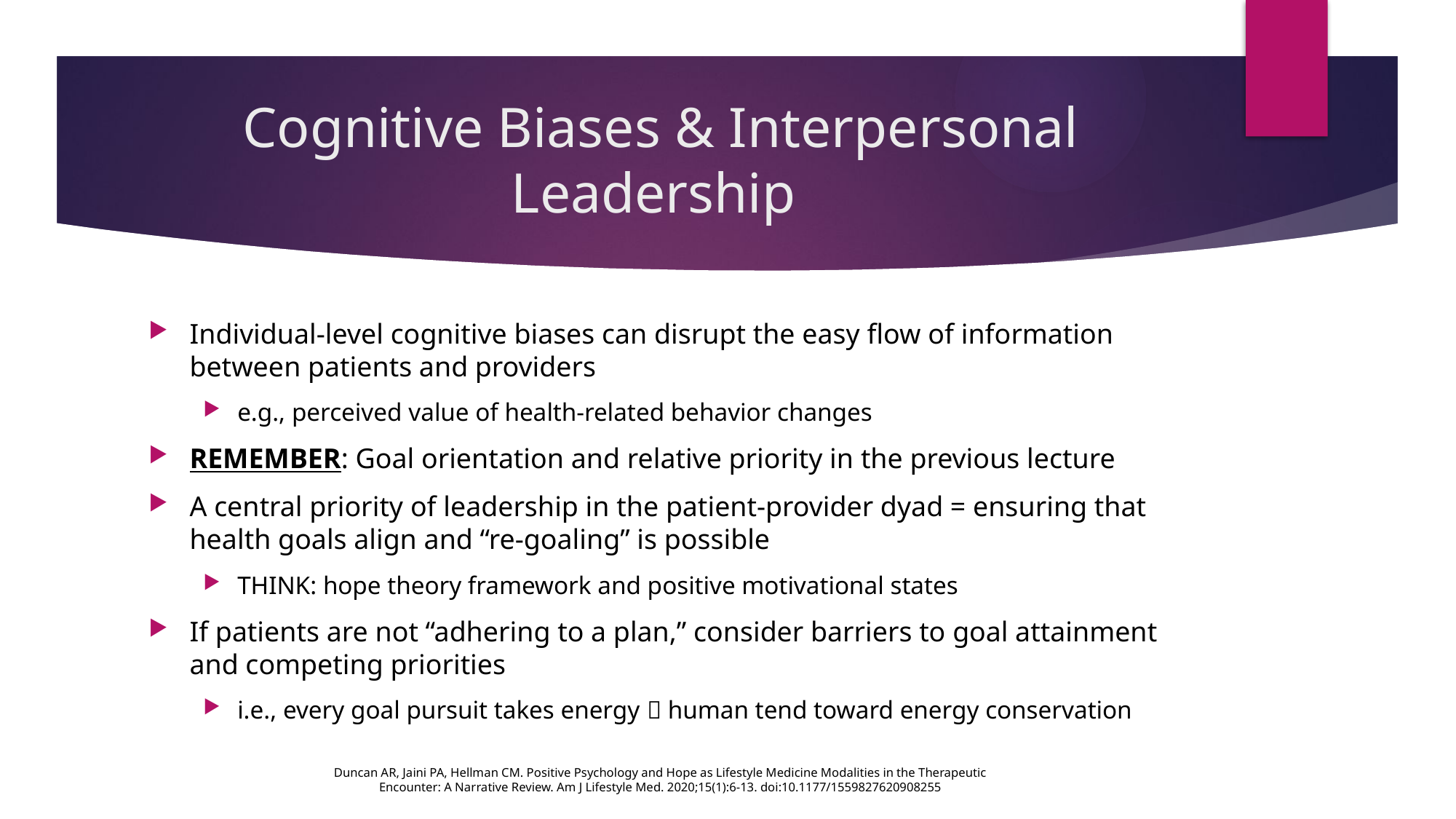

# Cognitive Biases & Interpersonal Leadership
Individual-level cognitive biases can disrupt the easy flow of information between patients and providers
e.g., perceived value of health-related behavior changes
REMEMBER: Goal orientation and relative priority in the previous lecture
A central priority of leadership in the patient-provider dyad = ensuring that health goals align and “re-goaling” is possible
THINK: hope theory framework and positive motivational states
If patients are not “adhering to a plan,” consider barriers to goal attainment and competing priorities
i.e., every goal pursuit takes energy  human tend toward energy conservation
Duncan AR, Jaini PA, Hellman CM. Positive Psychology and Hope as Lifestyle Medicine Modalities in the Therapeutic Encounter: A Narrative Review. Am J Lifestyle Med. 2020;15(1):6-13. doi:10.1177/1559827620908255

## Slide 13
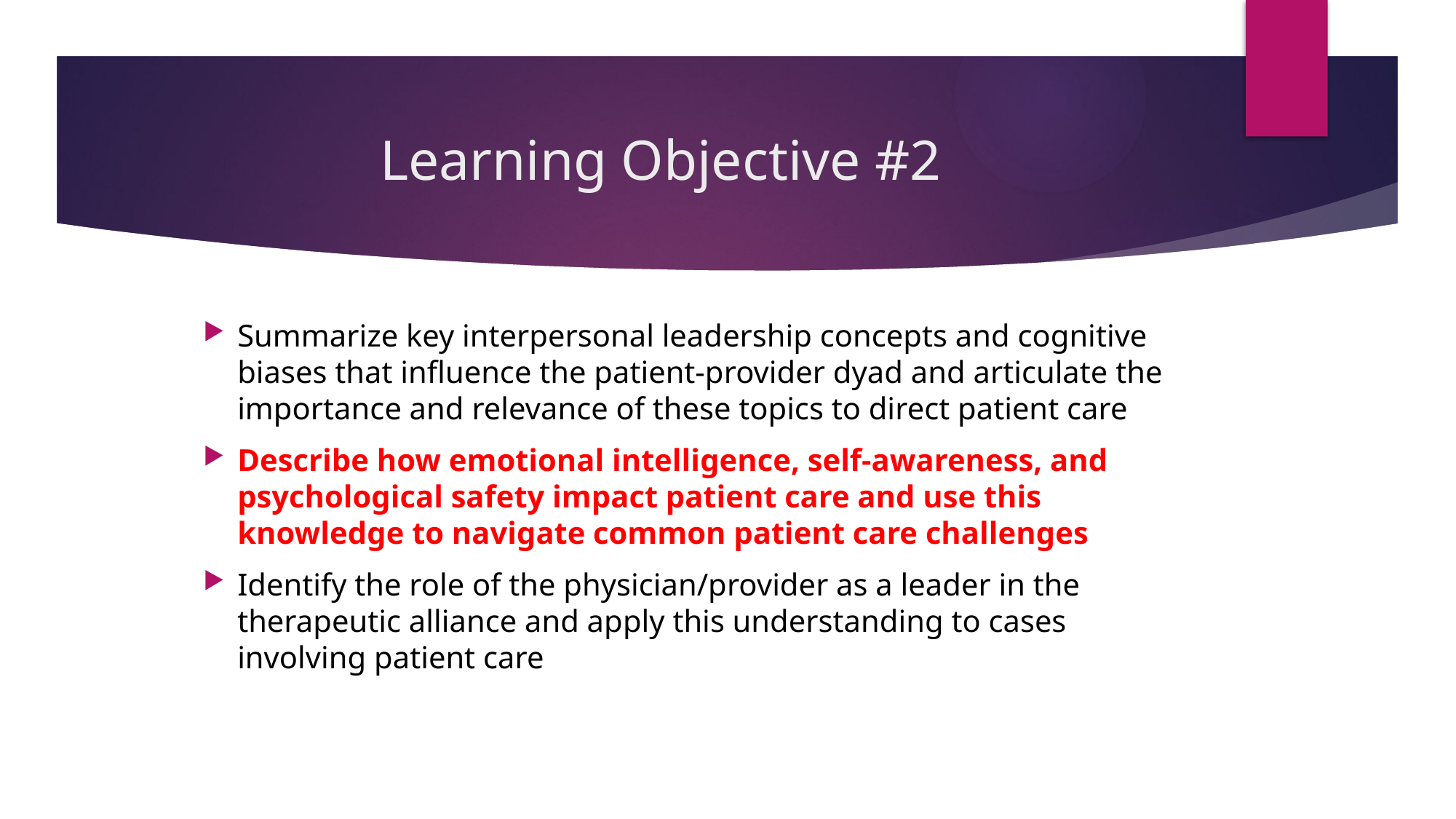

# Learning Objective #2
Summarize key interpersonal leadership concepts and cognitive biases that influence the patient-provider dyad and articulate the importance and relevance of these topics to direct patient care
Describe how emotional intelligence, self-awareness, and psychological safety impact patient care and use this knowledge to navigate common patient care challenges
Identify the role of the physician/provider as a leader in the therapeutic alliance and apply this understanding to cases involving patient care

## Slide 14
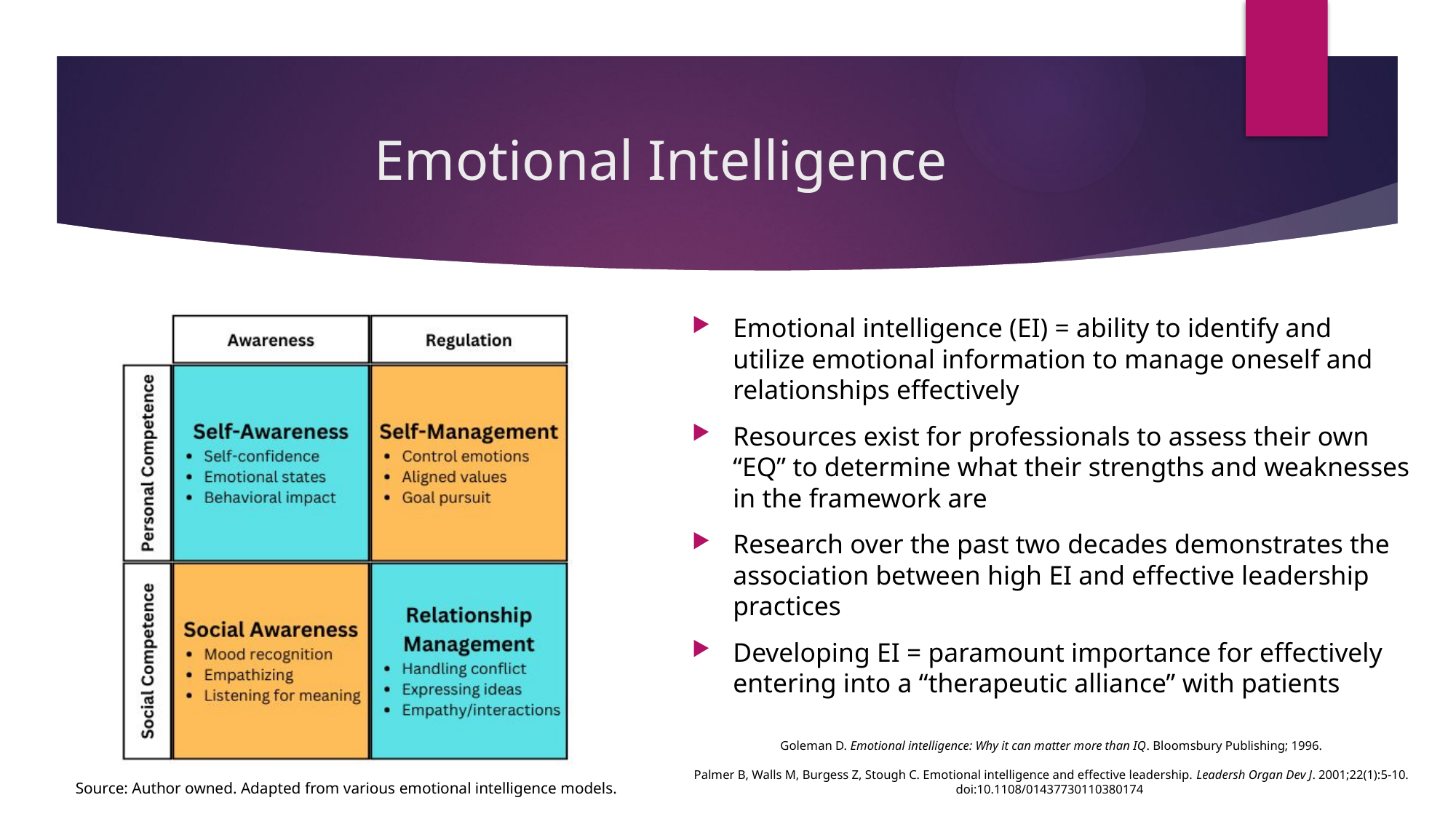

# Emotional Intelligence
Emotional intelligence (EI) = ability to identify and utilize emotional information to manage oneself and relationships effectively
Resources exist for professionals to assess their own “EQ” to determine what their strengths and weaknesses in the framework are
Research over the past two decades demonstrates the association between high EI and effective leadership practices
Developing EI = paramount importance for effectively entering into a “therapeutic alliance” with patients
Goleman D. Emotional intelligence: Why it can matter more than IQ. Bloomsbury Publishing; 1996.
Palmer B, Walls M, Burgess Z, Stough C. Emotional intelligence and effective leadership. Leadersh Organ Dev J. 2001;22(1):5-10. doi:10.1108/01437730110380174
Source: Author owned. Adapted from various emotional intelligence models.

## Slide 15
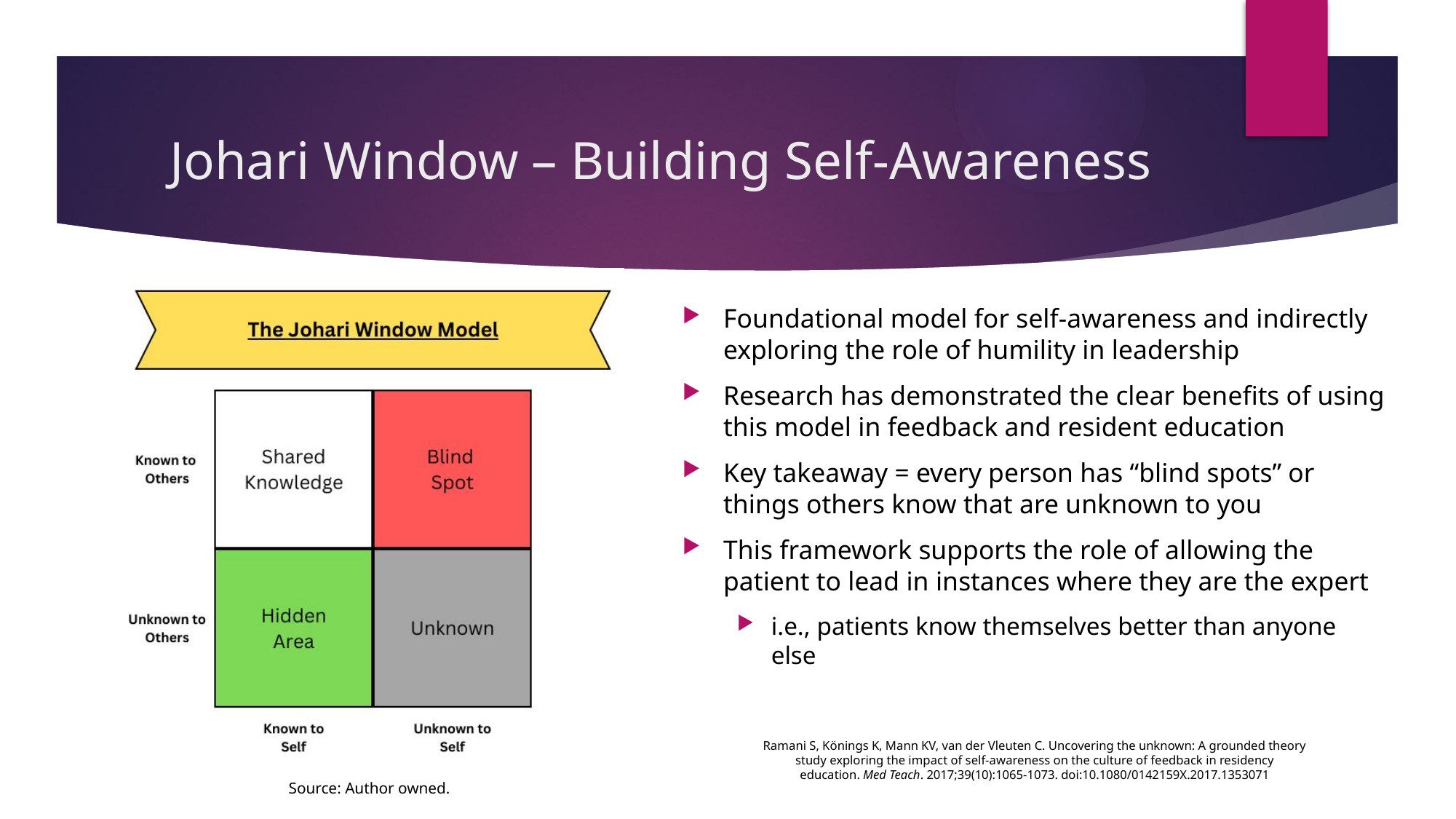

# Johari Window – Building Self-Awareness
Foundational model for self-awareness and indirectly exploring the role of humility in leadership
Research has demonstrated the clear benefits of using this model in feedback and resident education
Key takeaway = every person has “blind spots” or things others know that are unknown to you
This framework supports the role of allowing the patient to lead in instances where they are the expert
i.e., patients know themselves better than anyone else
Ramani S, Könings K, Mann KV, van der Vleuten C. Uncovering the unknown: A grounded theory study exploring the impact of self-awareness on the culture of feedback in residency education. Med Teach. 2017;39(10):1065-1073. doi:10.1080/0142159X.2017.1353071
Source: Author owned.

## Slide 16
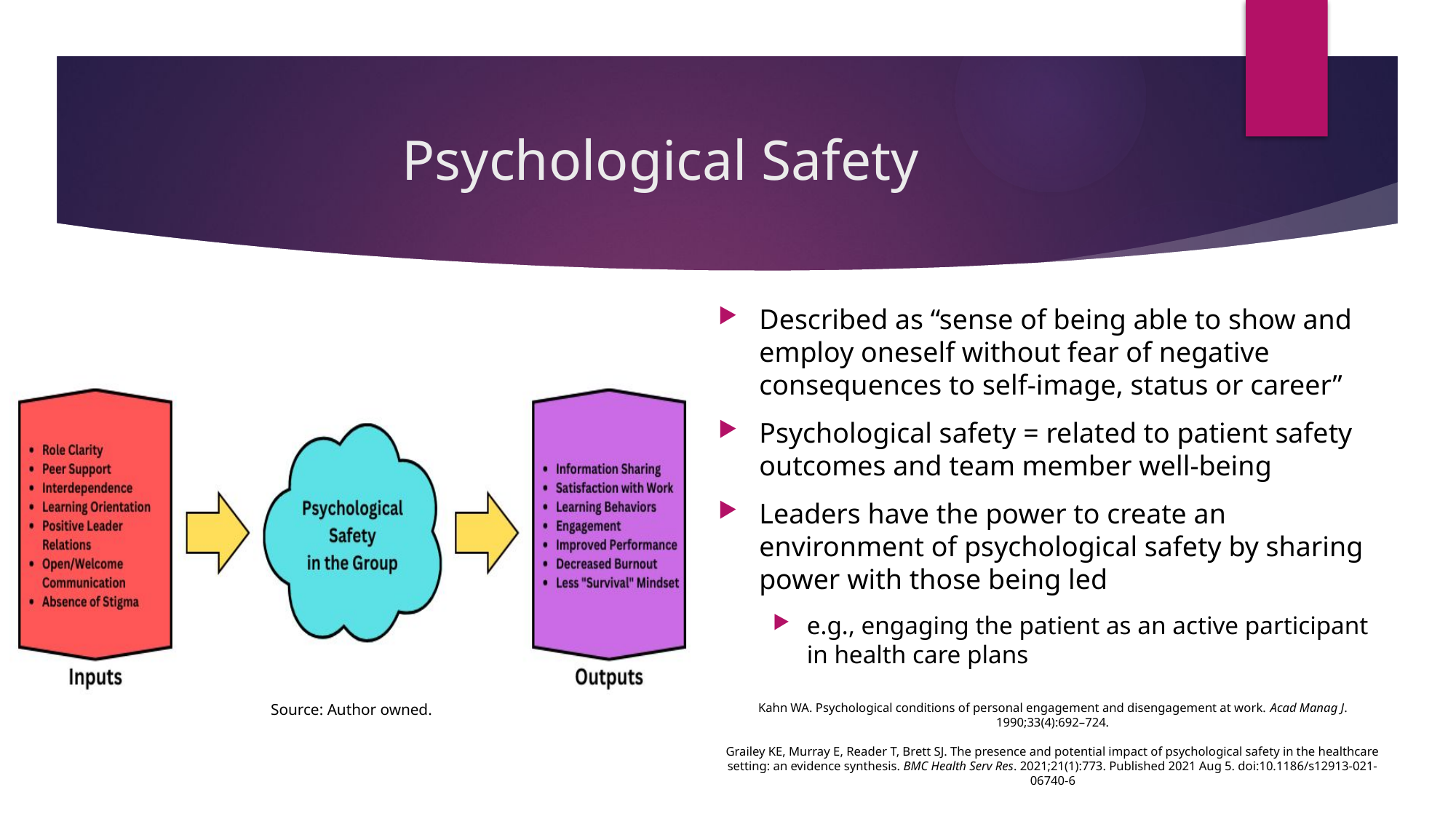

# Psychological Safety
Described as “sense of being able to show and employ oneself without fear of negative consequences to self-image, status or career”
Psychological safety = related to patient safety outcomes and team member well-being
Leaders have the power to create an environment of psychological safety by sharing power with those being led
e.g., engaging the patient as an active participant in health care plans
Source: Author owned.
Kahn WA. Psychological conditions of personal engagement and disengagement at work. Acad Manag J. 1990;33(4):692–724.
Grailey KE, Murray E, Reader T, Brett SJ. The presence and potential impact of psychological safety in the healthcare setting: an evidence synthesis. BMC Health Serv Res. 2021;21(1):773. Published 2021 Aug 5. doi:10.1186/s12913-021-06740-6

## Slide 17
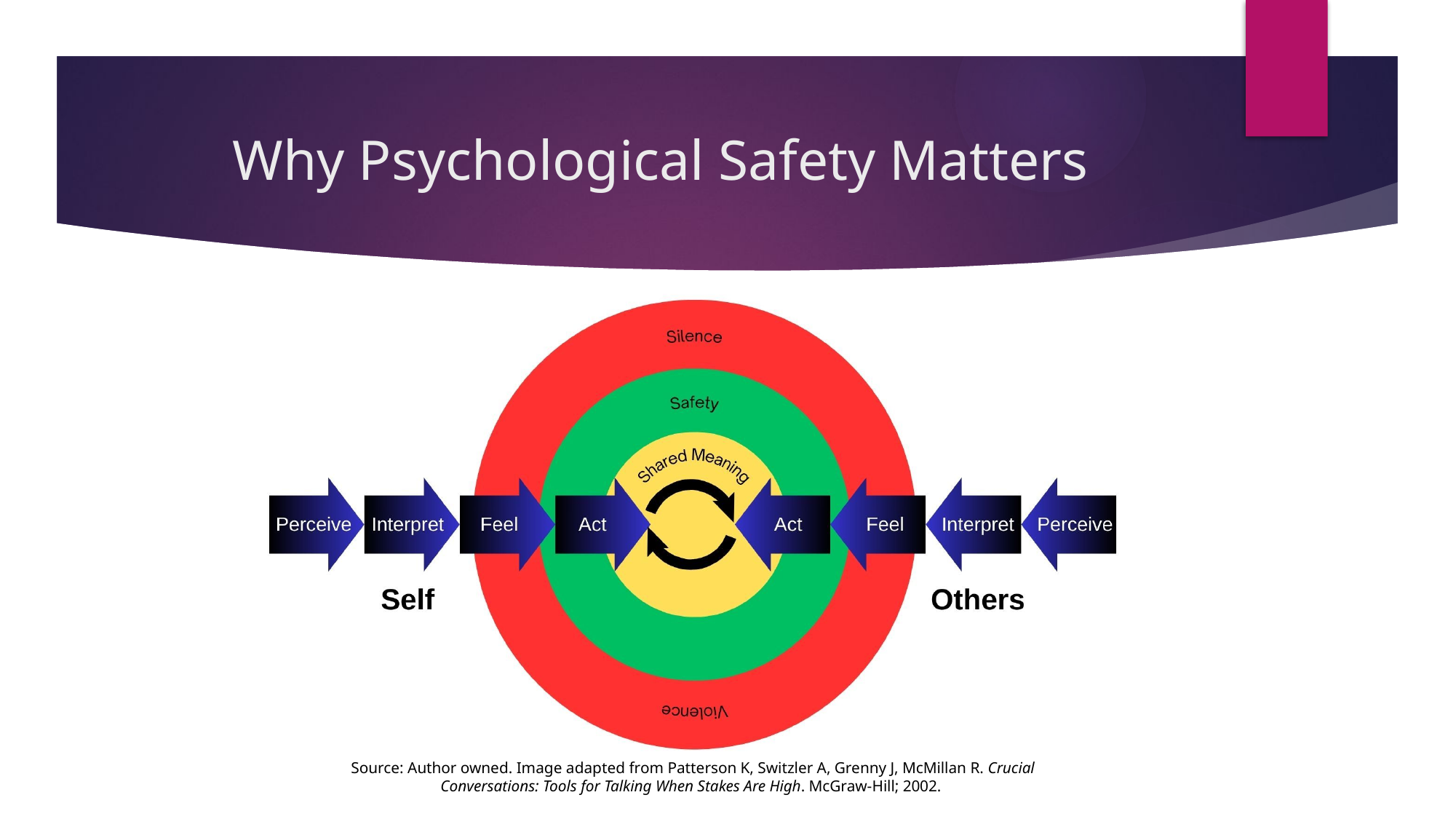

# Why Psychological Safety Matters
Source: Author owned. Image adapted from Patterson K, Switzler A, Grenny J, McMillan R. Crucial Conversations: Tools for Talking When Stakes Are High. McGraw-Hill; 2002.

## Slide 18
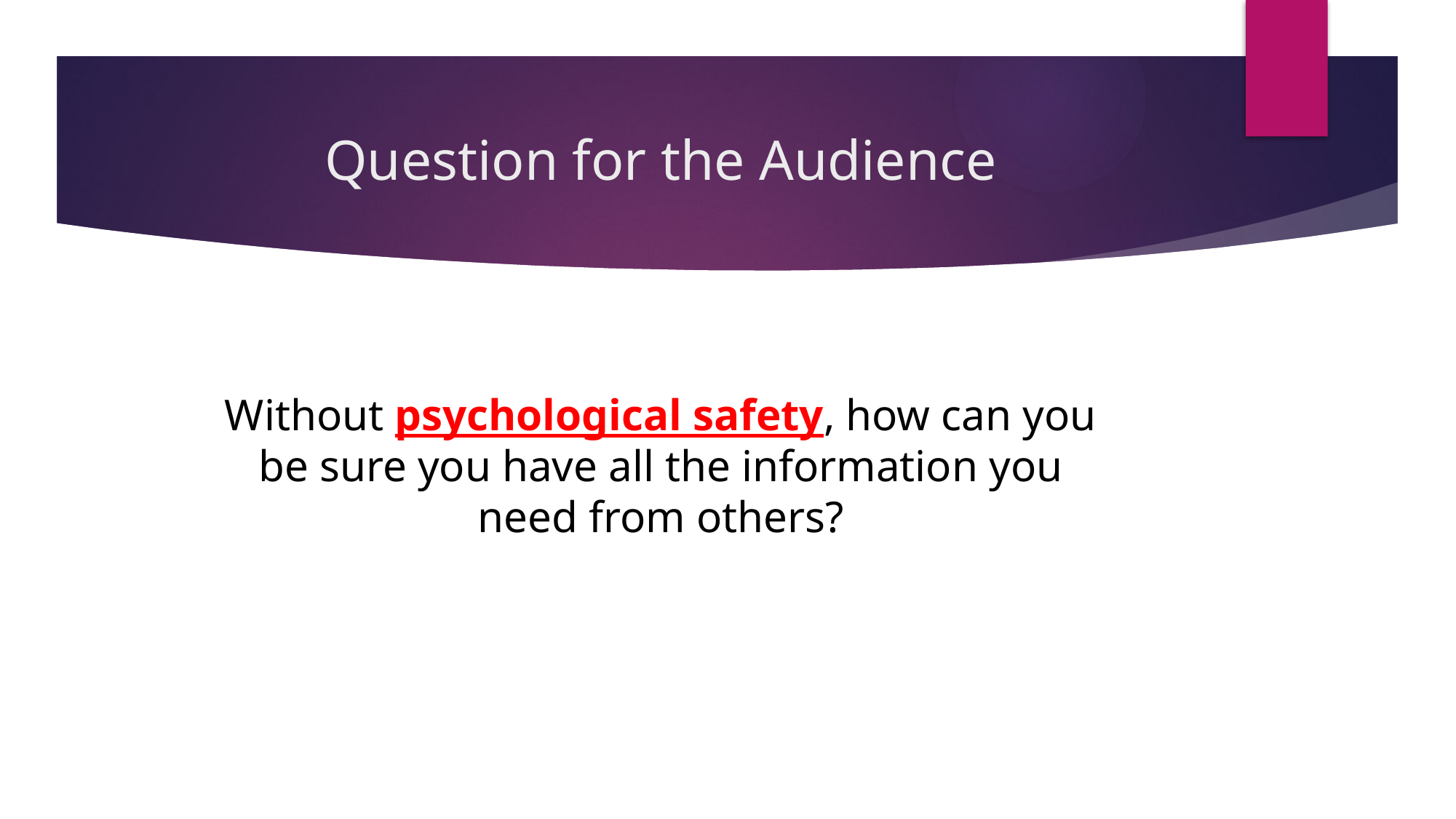

# Question for the Audience
Without psychological safety, how can you be sure you have all the information you need from others?

## Slide 19
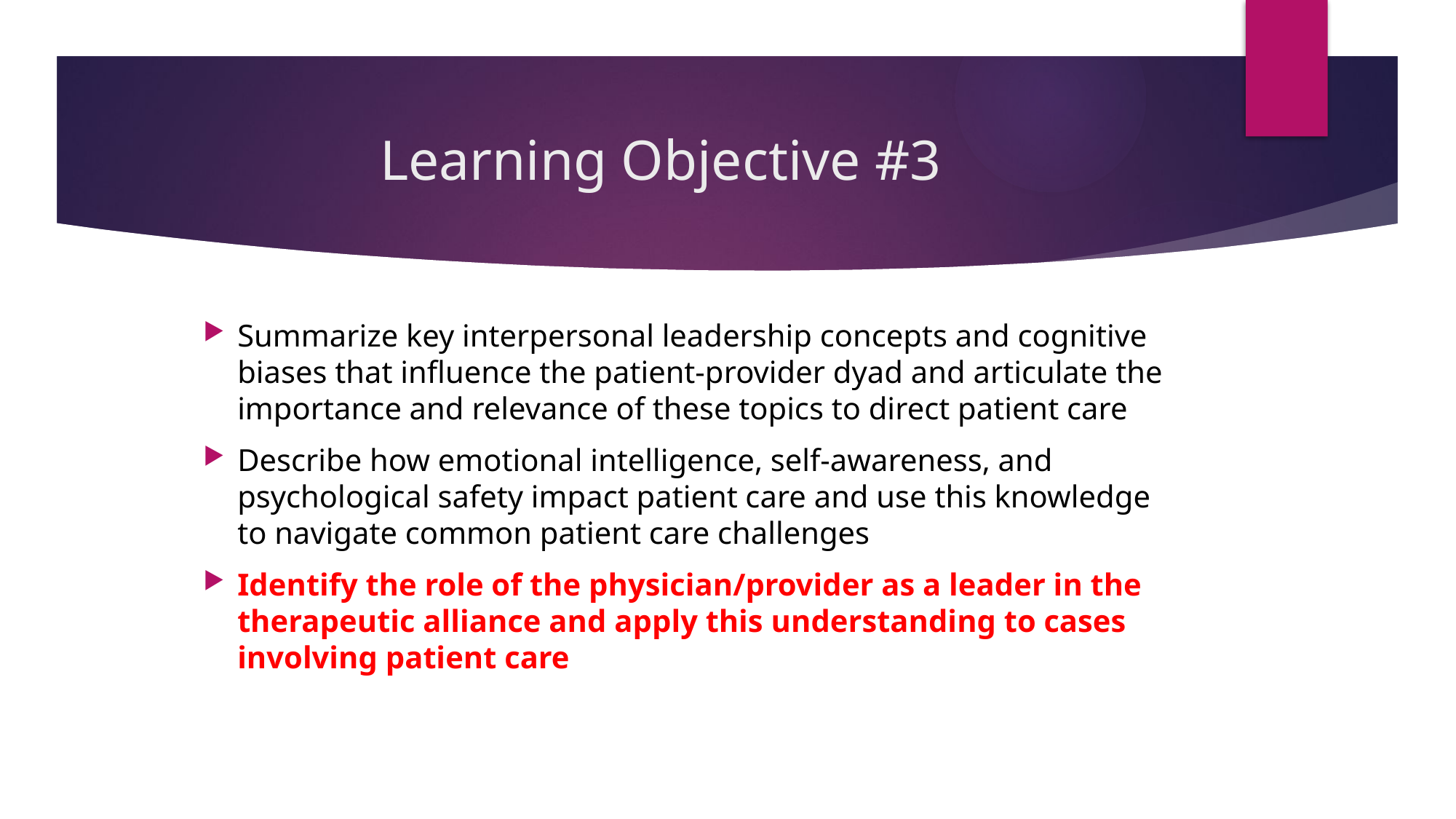

# Learning Objective #3
Summarize key interpersonal leadership concepts and cognitive biases that influence the patient-provider dyad and articulate the importance and relevance of these topics to direct patient care
Describe how emotional intelligence, self-awareness, and psychological safety impact patient care and use this knowledge to navigate common patient care challenges
Identify the role of the physician/provider as a leader in the therapeutic alliance and apply this understanding to cases involving patient care

## Slide 20
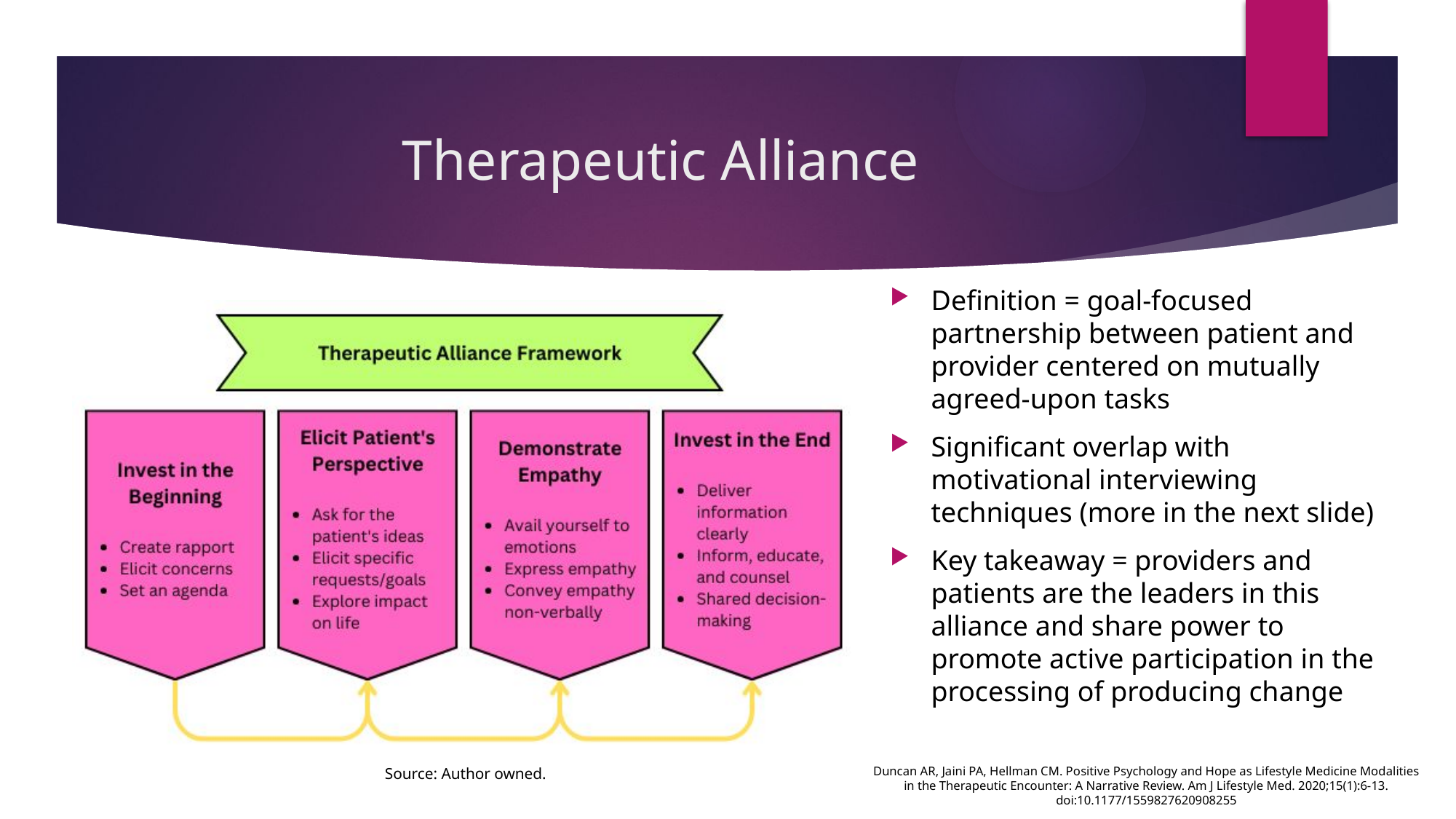

# Therapeutic Alliance
Definition = goal-focused partnership between patient and provider centered on mutually agreed-upon tasks
Significant overlap with motivational interviewing techniques (more in the next slide)
Key takeaway = providers and patients are the leaders in this alliance and share power to promote active participation in the processing of producing change
Source: Author owned.
Duncan AR, Jaini PA, Hellman CM. Positive Psychology and Hope as Lifestyle Medicine Modalities in the Therapeutic Encounter: A Narrative Review. Am J Lifestyle Med. 2020;15(1):6-13. doi:10.1177/1559827620908255

## Slide 21
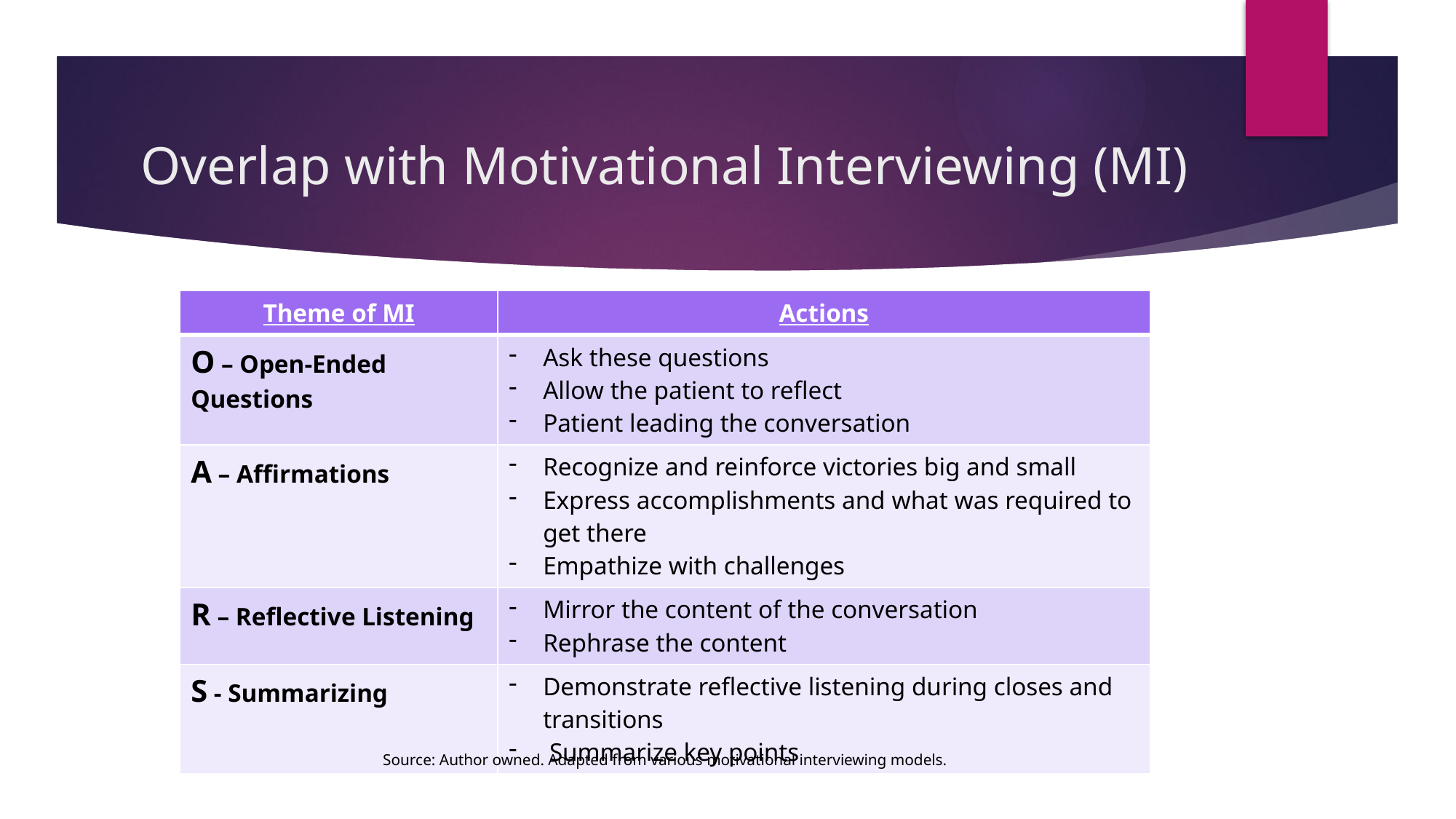

# Overlap with Motivational Interviewing (MI)
| Theme of MI | Actions |
| --- | --- |
| O – Open-Ended Questions | Ask these questions Allow the patient to reflect Patient leading the conversation |
| A – Affirmations | Recognize and reinforce victories big and small Express accomplishments and what was required to get there Empathize with challenges |
| R – Reflective Listening | Mirror the content of the conversation Rephrase the content |
| S - Summarizing | Demonstrate reflective listening during closes and transitions Summarize key points |
Source: Author owned. Adapted from various motivational interviewing models.

## Slide 22
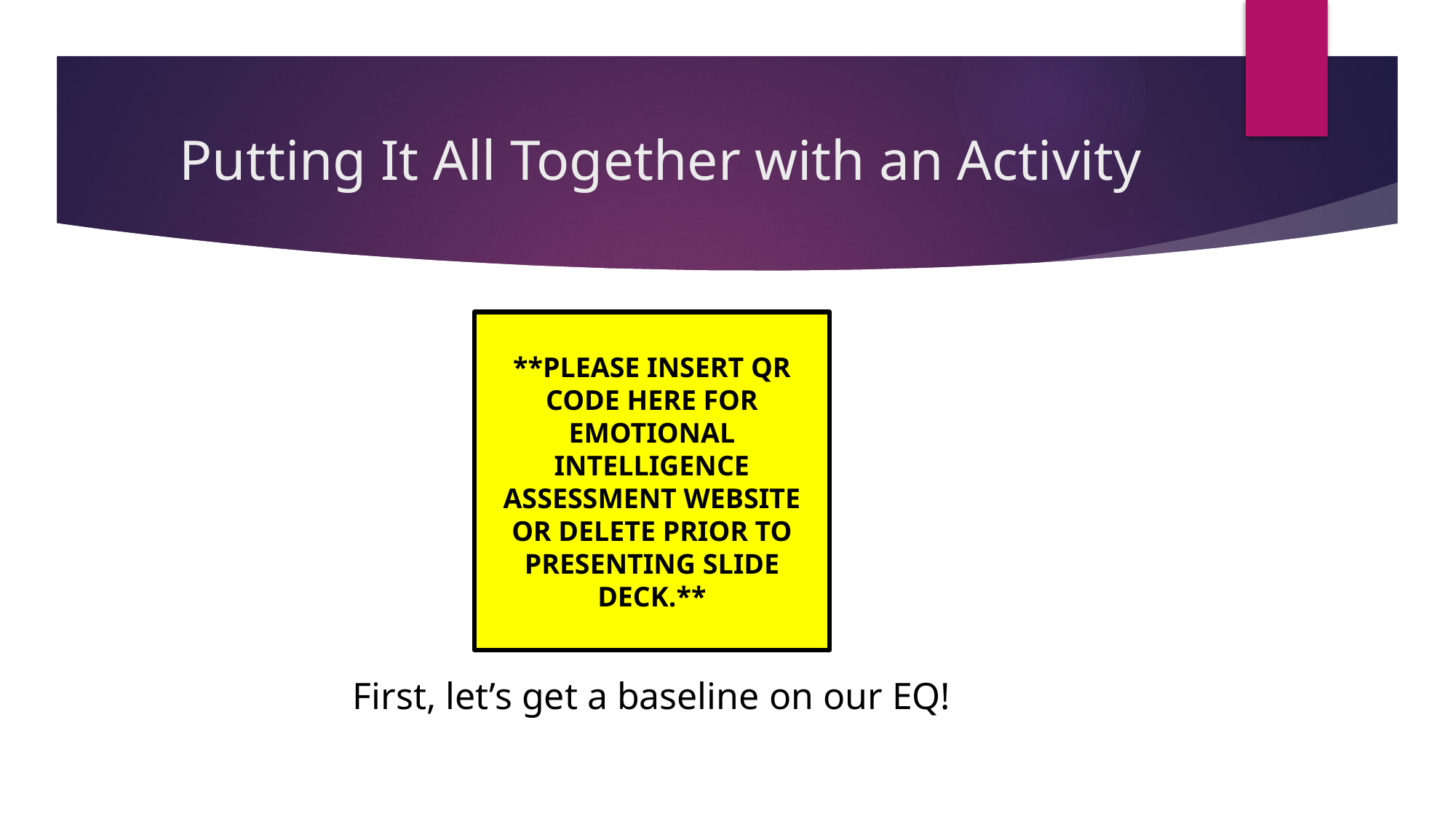

# Putting It All Together with an Activity
**PLEASE INSERT QR CODE HERE FOR EMOTIONAL INTELLIGENCE ASSESSMENT WEBSITE OR DELETE PRIOR TO PRESENTING SLIDE DECK.**
First, let’s get a baseline on our EQ!

## Slide 23
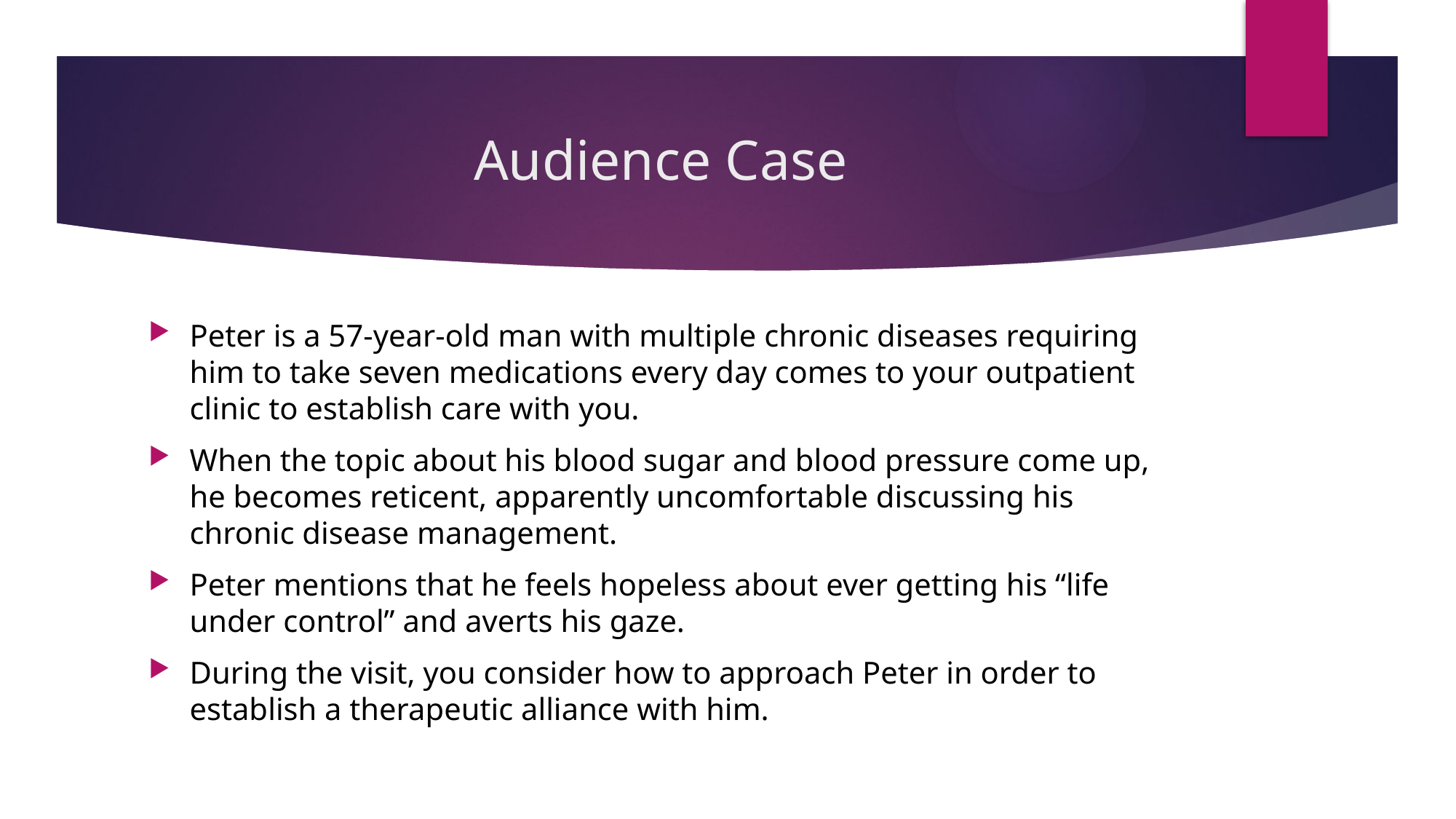

# Audience Case
Peter is a 57-year-old man with multiple chronic diseases requiring him to take seven medications every day comes to your outpatient clinic to establish care with you.
When the topic about his blood sugar and blood pressure come up, he becomes reticent, apparently uncomfortable discussing his chronic disease management.
Peter mentions that he feels hopeless about ever getting his “life under control” and averts his gaze.
During the visit, you consider how to approach Peter in order to establish a therapeutic alliance with him.

## Slide 24
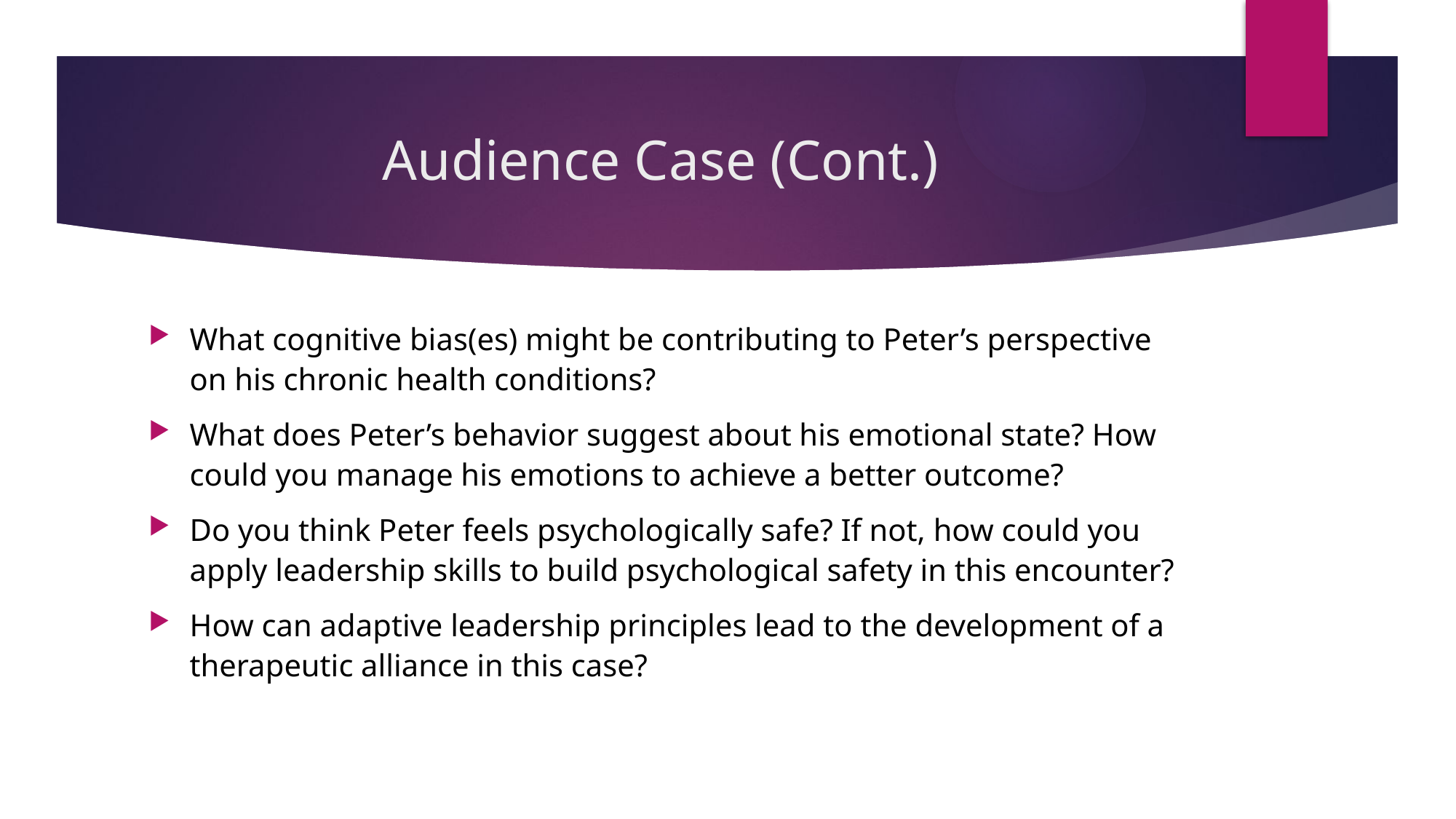

# Audience Case (Cont.)
What cognitive bias(es) might be contributing to Peter’s perspective on his chronic health conditions?
What does Peter’s behavior suggest about his emotional state? How could you manage his emotions to achieve a better outcome?
Do you think Peter feels psychologically safe? If not, how could you apply leadership skills to build psychological safety in this encounter?
How can adaptive leadership principles lead to the development of a therapeutic alliance in this case?

## Slide 25
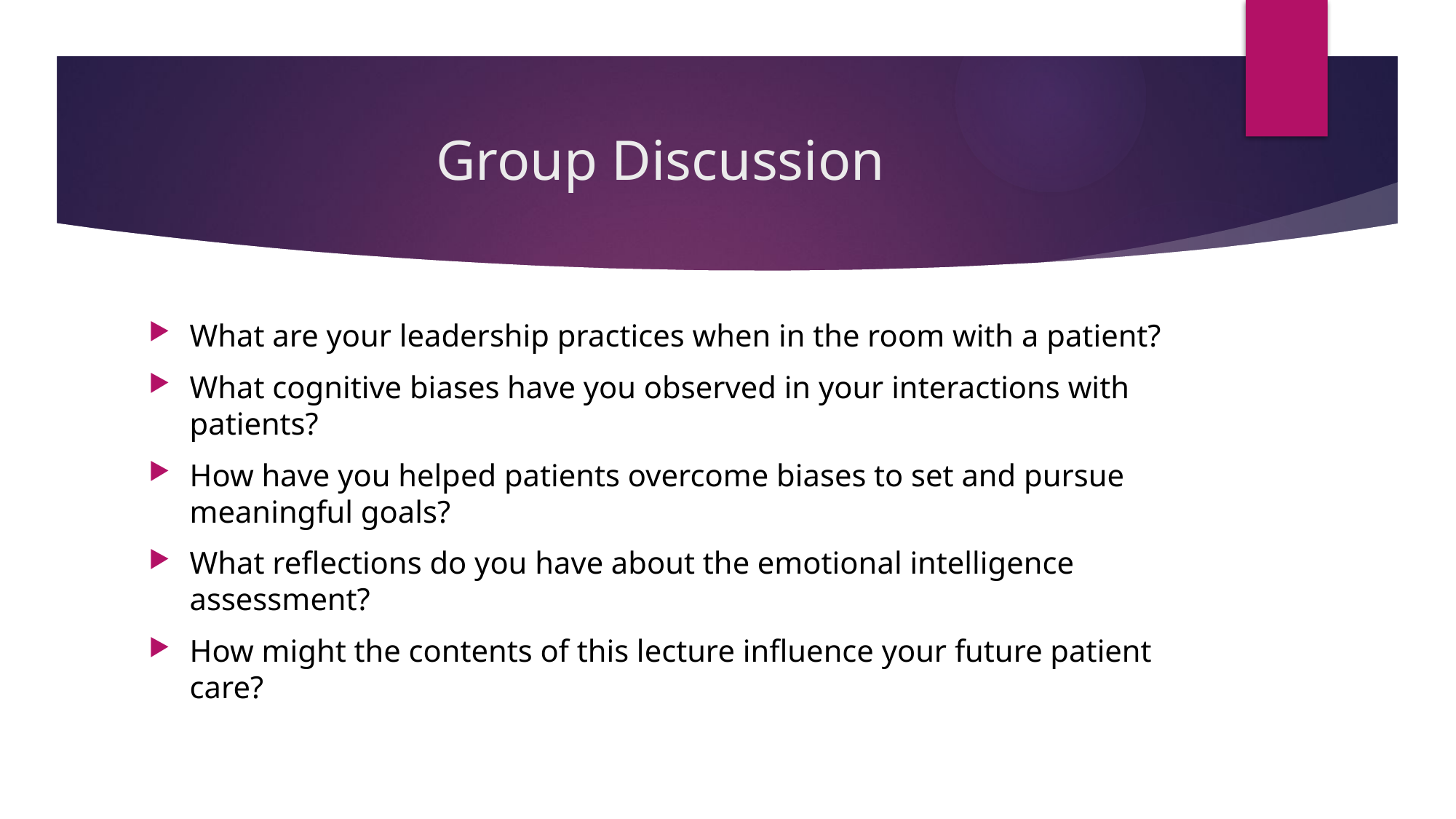

# Group Discussion
What are your leadership practices when in the room with a patient?
What cognitive biases have you observed in your interactions with patients?
How have you helped patients overcome biases to set and pursue meaningful goals?
What reflections do you have about the emotional intelligence assessment?
How might the contents of this lecture influence your future patient care?

## Slide 26
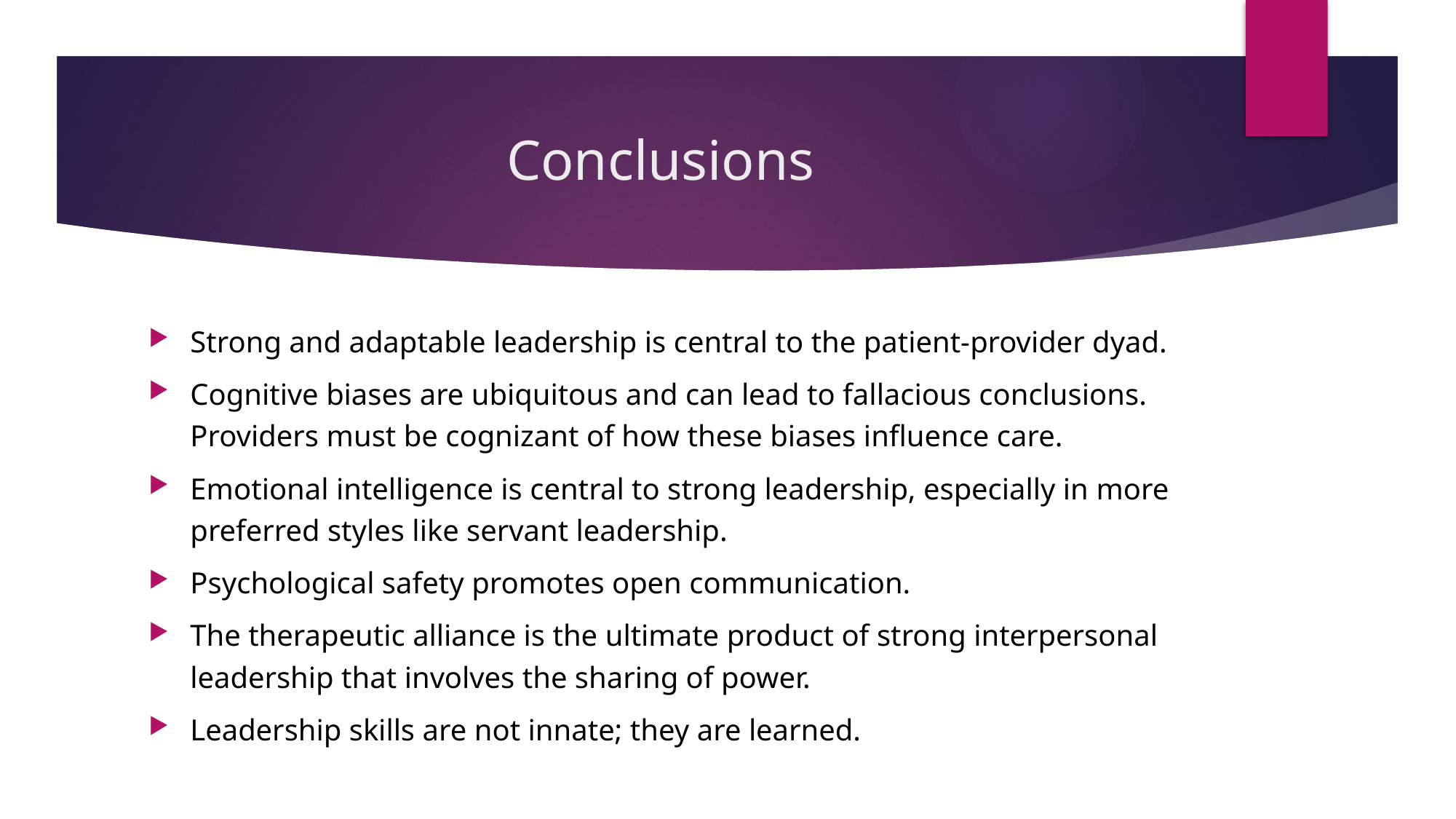

# Conclusions
Strong and adaptable leadership is central to the patient-provider dyad.
Cognitive biases are ubiquitous and can lead to fallacious conclusions. Providers must be cognizant of how these biases influence care.
Emotional intelligence is central to strong leadership, especially in more preferred styles like servant leadership.
Psychological safety promotes open communication.
The therapeutic alliance is the ultimate product of strong interpersonal leadership that involves the sharing of power.
Leadership skills are not innate; they are learned.

## Slide 27
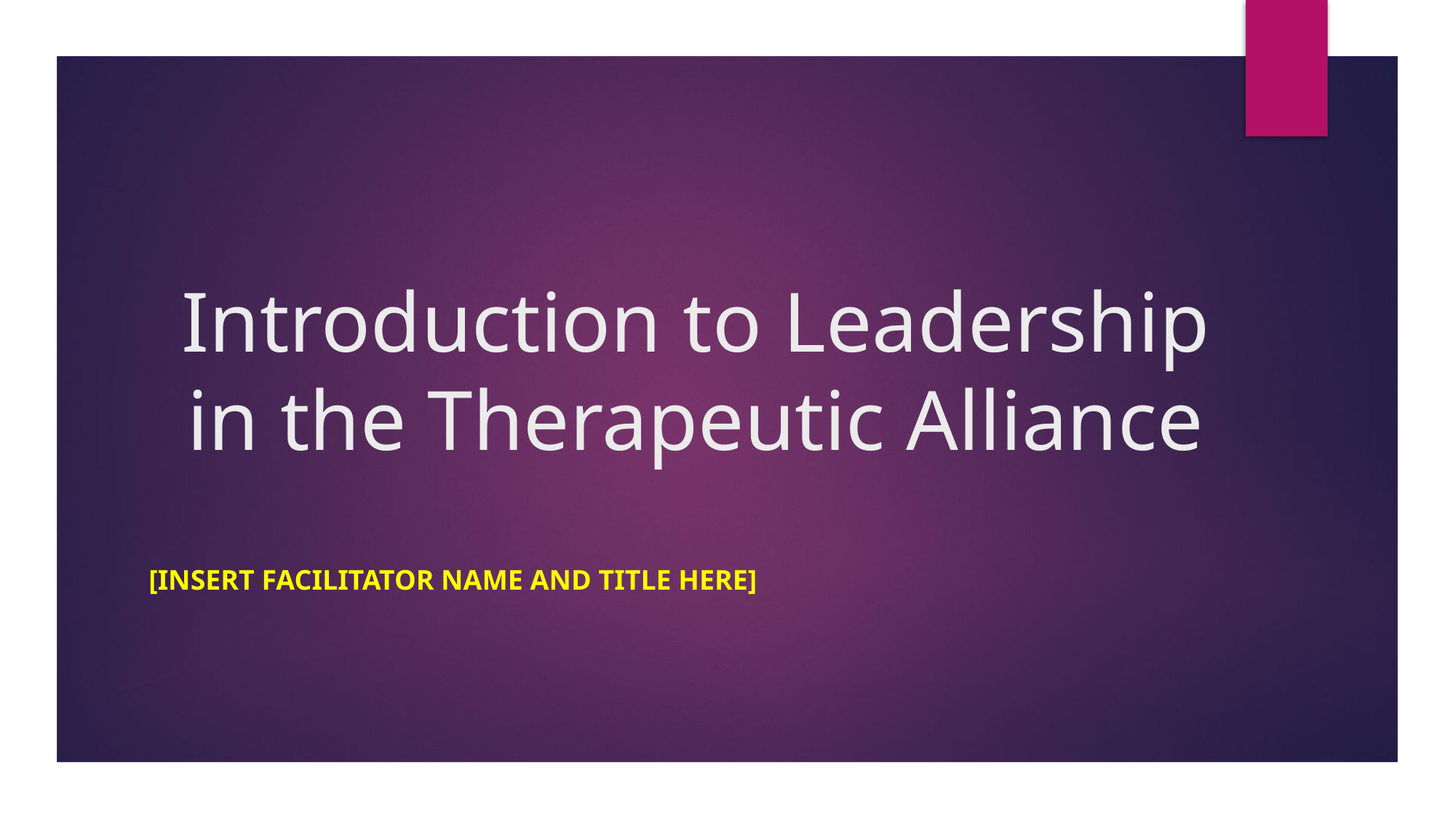

# Introduction to Leadership in the Therapeutic Alliance
[INSERT FACILITATOR NAME AND TITLE HERE]
