# Supplementary material for: How Positive Psychology Can Augment Leadership Through the Therapeutic Alliance
Source: MedEdPORTAL. 2025 Mar 20;21:11510. doi: 10.15766/mep_2374-8265.11510 (PMC11922800; doi:10.15766/mep_2374-8265.11510)
Supplement: Supplementary file 1 — Intro to Positive Psychology.pptxIntro to Leadership in the Therapeutic Alliance.pptxFacilitator Guide.docxSurveys.docx [file mep_2374-8265.11510-s001.zip › D. Surveys.docx]

Introduction to Positive Psychology & Leadership in the Therapeutic Alliance - Pre-Participation Survey

1. How do you identify in terms of race/ethnicity? Mark all that apply.
   - American Indian / Alaskan Native
   - Asian / Pacific Islander
   - Black or African American
   - Hispanic
   - White / Caucasian
   - Other: ___________________
2. How do you identify in terms of gender? *Mark only one oval.*
   - Cisgender Male
   - Cisgender Female
   - Transgender Male
   - Transgender Female
   - Other: ___________________
3. What is your age?

______________________

1. What is your educational level?

- Medical student
- Resident
- Fellow
- Faculty
- Other: ___________________

1. What is your medical specialty?

- Family medicine
- Internal medicine
- Pediatrics
- Psychiatry
- General surgery
- Obstetrics and gynecology
- Other: ___________________

Please respond to each question on a five-point Likert scale based on how important or unimportant you feel each topic is to directly caring for patients.

1. How important do you feel positive psychology is to caring for patients in different clinical settings?

- Very unimportant
- Unimportant
- Neutral
- Important
- Very important
- N/A; I do not know enough about the topic to comment

1. How important do you feel goal prioritization is to caring for patients in different clinical settings?

- Very unimportant
- Unimportant
- Neutral
- Important
- Very important
- N/A; I do not know enough about the topic to comment

1. How important do you feel interpersonal leadership is to caring for patients in different clinical settings?
   - Very unimportant
   - Unimportant
   - Neutral
   - Important
   - Very important
   - N/A; I do not know enough about the topic to comment
2. How important do you feel the therapeutic alliance is to caring for patients in different clinical settings?
   - Very unimportant
   - Unimportant
   - Neutral
   - Important
   - Very important
   - N/A; I do not know enough about the topic to comment
3. How important do you feel emotional intelligence is to caring for patients in different clinical settings?
   - Very unimportant
   - Unimportant
   - Neutral
   - Important
   - Very important
   - N/A; I do not know enough about the topic to comment
4. How important do you feel psychological safety is to caring for patients in different clinical settings?
   - Very unimportant
   - Unimportant
   - Neutral
   - Important
   - Very important
   - N/A; I do not know enough about the topic to comment

Please respond to each question on a five-point Likert scale based on how confident you feel about each topic.

1. How confident do you feel about applying the science of positive psychology to direct patient care?
   - Very unconfident
   - Unconfident
   - Neutral
   - Confident
   - Very confident
2. How confident do you feel about applying principles of leadership to developing a therapeutic alliance with patients?
   - Very unconfident
   - Unconfident
   - Neutral
   - Confident
   - Very confident

Please respond to each statement on a five-point Likert scale ranging from "Strongly disagree" to "Strongly agree."

1. I am familiar with common positive psychology concepts and their operational definitions (e.g., flow, flourishing, hope, etc).
   - Strongly disagree
   - Disagree
   - Neutral
   - Agree
   - Strongly Agree
2. I am knowledgeable about the basic neurobiology of key positive psychology traits and how the brain changes in response to these traits.
   - Strongly disagree
   - Disagree
   - Neutral
   - Agree
   - Strongly Agree
3. I can classify the components of hope to nurturing well-being and use this information to improve patient care.
   - Strongly disagree
   - Disagree
   - Neutral
   - Agree
   - Strongly Agree
4. I can describe how goal prioritization can promote better health and how this concept applies to the communities I serve.
   - Strongly disagree
   - Disagree
   - Neutral
   - Agree
   - Strongly Agree
5. I am familiar with how key interpersonal leadership concepts and cognitive biases influence the patient-provider dyad.
   - Strongly disagree
   - Disagree
   - Neutral
   - Agree
   - Strongly Agree
6. I am able to describe how emotional intelligence, self-awareness, and psychological safety impact patient care.
   - Strongly disagree
   - Disagree
   - Neutral
   - Agree
   - Strongly Agree
7. I can describe the role of the physician/provider as the leader in the therapeutic alliance.
   - Strongly disagree
   - Disagree
   - Neutral
   - Agree
   - Strongly Agree

To receive your follow-up survey, please provide your email:

_________________________________________________

Introduction to Positive Psychology & Leadership in the Therapeutic Alliance - Post-Participation Survey

1. How do you identify in terms of race/ethnicity? Mark all that apply.
   - American Indian / Alaskan Native
   - Asian / Pacific Islander
   - Black or African American
   - Hispanic
   - White / Caucasian
   - Other: ___________________
2. How do you identify in terms of gender? *Mark only one oval.*
   - Cisgender Male
   - Cisgender Female
   - Transgender Male
   - Transgender Female
   - Other: ___________________
3. What is your age?

______________________

1. What is your educational level?

- Medical student
- Resident
- Fellow
- Faculty
- Other: ___________________

1. What is your medical specialty?

- Family medicine
- Internal medicine
- Pediatrics
- Psychiatry
- General surgery
- Obstetrics and gynecology
- Other: ___________________

Please respond to each question on a five-point Likert scale based on how important or unimportant you feel each topic is to directly caring for patients.

1. How important do you feel positive psychology is to caring for patients in different clinical settings?

- Very unimportant
- Unimportant
- Neutral
- Important
- Very important
- N/A; I do not know enough about the topic to comment

1. How important do you feel goal prioritization is to caring for patients in different clinical settings?

- Very unimportant
- Unimportant
- Neutral
- Important
- Very important
- N/A; I do not know enough about the topic to comment

1. How important do you feel interpersonal leadership is to caring for patients in different clinical settings?
   - Very unimportant
   - Unimportant
   - Neutral
   - Important
   - Very important
   - N/A; I do not know enough about the topic to comment
2. How important do you feel the therapeutic alliance is to caring for patients in different clinical settings?
   - Very unimportant
   - Unimportant
   - Neutral
   - Important
   - Very important
   - N/A; I do not know enough about the topic to comment
3. How important do you feel emotional intelligence is to caring for patients in different clinical settings?
   - Very unimportant
   - Unimportant
   - Neutral
   - Important
   - Very important
   - N/A; I do not know enough about the topic to comment
4. How important do you feel psychological safety is to caring for patients in different clinical settings?
   - Very unimportant
   - Unimportant
   - Neutral
   - Important
   - Very important
   - N/A; I do not know enough about the topic to comment

Please respond to each question on a five-point Likert scale based on how confident you feel about each topic.

1. How confident do you feel about applying the science of positive psychology to direct patient care?
   - Very unconfident
   - Unconfident
   - Neutral
   - Confident
   - Very confident
2. How confident do you feel about applying principles of leadership to developing a therapeutic alliance with patients?
   - Very unconfident
   - Unconfident
   - Neutral
   - Confident
   - Very confident

Please respond to each statement on a five-point Likert scale ranging from "Strongly disagree" to "Strongly agree."

1. I am familiar with common positive psychology concepts and their operational definitions (e.g., flow, flourishing, hope, etc).
   - Strongly disagree
   - Disagree
   - Neutral
   - Agree
   - Strongly Agree
2. I am knowledgeable about the basic neurobiology of key positive psychology traits and how the brain changes in response to these traits.
   - Strongly disagree
   - Disagree
   - Neutral
   - Agree
   - Strongly Agree
3. I can classify the components of hope to nurturing well-being and use this information to improve patient care.
   - Strongly disagree
   - Disagree
   - Neutral
   - Agree
   - Strongly Agree
4. I can describe how goal prioritization can promote better health and how this concept applies to the communities I serve.
   - Strongly disagree
   - Disagree
   - Neutral
   - Agree
   - Strongly Agree
5. I am familiar with how key interpersonal leadership concepts and cognitive biases influence the patient-provider dyad.
   - Strongly disagree
   - Disagree
   - Neutral
   - Agree
   - Strongly Agree
6. I am able to describe how emotional intelligence, self-awareness, and psychological safety impact patient care.
   - Strongly disagree
   - Disagree
   - Neutral
   - Agree
   - Strongly Agree
7. I can describe the role of the physician/provider as the leader in the therapeutic alliance.
   - Strongly disagree
   - Disagree
   - Neutral
   - Agree
   - Strongly Agree

Please provide final reflections on this module in the sections below.

1. Based on the contents of this module, what changes do you plan to make to your practice of medicine to improve your patient care?

______________________________________________________________________________

______________________________________________________________________________

______________________________________________________________________________

______________________________________________________________________________

1. What were the strengths of this module?

______________________________________________________________________________

______________________________________________________________________________

______________________________________________________________________________

______________________________________________________________________________

1. What changes would you make to this module to improve the content delivery?

______________________________________________________________________________

______________________________________________________________________________

______________________________________________________________________________

______________________________________________________________________________

To receive your follow-up survey, please provide your email:

_________________________________________________

Introduction to Positive Psychology & Leadership in the Therapeutic Alliance - Follow-up Survey

Please provide feedback on any changes to your direct patient care practices or behaviors in health care settings that have occurred as a result of the positive psychology and interpersonal leadership content you received 6-8 weeks ago.

1. How do you identify in terms of race/ethnicity? Mark all that apply.
   - American Indian / Alaskan Native
   - Asian / Pacific Islander
   - Black or African American
   - Hispanic
   - White / Caucasian
   - Other: ___________________
2. How do you identify in terms of gender? *Mark only one oval.*
   - Cisgender Male
   - Cisgender Female
   - Transgender Male
   - Transgender Female
   - Other: ___________________
3. What is your age?

______________________

1. What is your educational level?

- Medical student
- Resident
- Fellow
- Faculty
- Other: ___________________

1. What is your medical specialty?

- Family medicine
- Internal medicine
- Pediatrics
- Psychiatry
- General surgery
- Obstetrics and gynecology
- Other: ___________________

1. On a scale from 1 to 10, to what extent did the positive psychology content (i.e., overview of key frameworks, relevant neurobiology, hope-nurturing strategies, and goal prioritization) you received influence your patient care practices?

_______

1. If any, what specific changes have you made based on the positive psychology content?

______________________________________________________________________________

______________________________________________________________________________

1. On a scale from 1 to 10, to what extent did the interpersonal leadership content (i.e., overview of key concepts, cognitive biases, emotional intelligence, self- awareness, psychological safety, and therapeutic alliance) you received influence your patient care practices?

_______

1. If any, what specific changes have you made based on the interpersonal leadership content?

______________________________________________________________________________

______________________________________________________________________________

1. On a scale from 1 to 10, how relevant do you feel the positive psychology and leadership content is to direct patient care?

_______

1. What feedback do you have about the content now that several weeks have passed? This section is optional.

______________________________________________________________________________

______________________________________________________________________________

______________________________________________________________________________

______________________________________________________________________________
