# Supplementary material for: How Positive Psychology Can Augment Leadership Through the Therapeutic Alliance
Source: MedEdPORTAL. 2025 Mar 20;21:11510. doi: 10.15766/mep_2374-8265.11510 (PMC11922800; doi:10.15766/mep_2374-8265.11510)
Supplement: Supplementary file 1 — Intro to Positive Psychology.pptxIntro to Leadership in the Therapeutic Alliance.pptxFacilitator Guide.docxSurveys.docx [file mep_2374-8265.11510-s001.zip › A. Intro to Positive Psychology.pptx]

## Slide 1
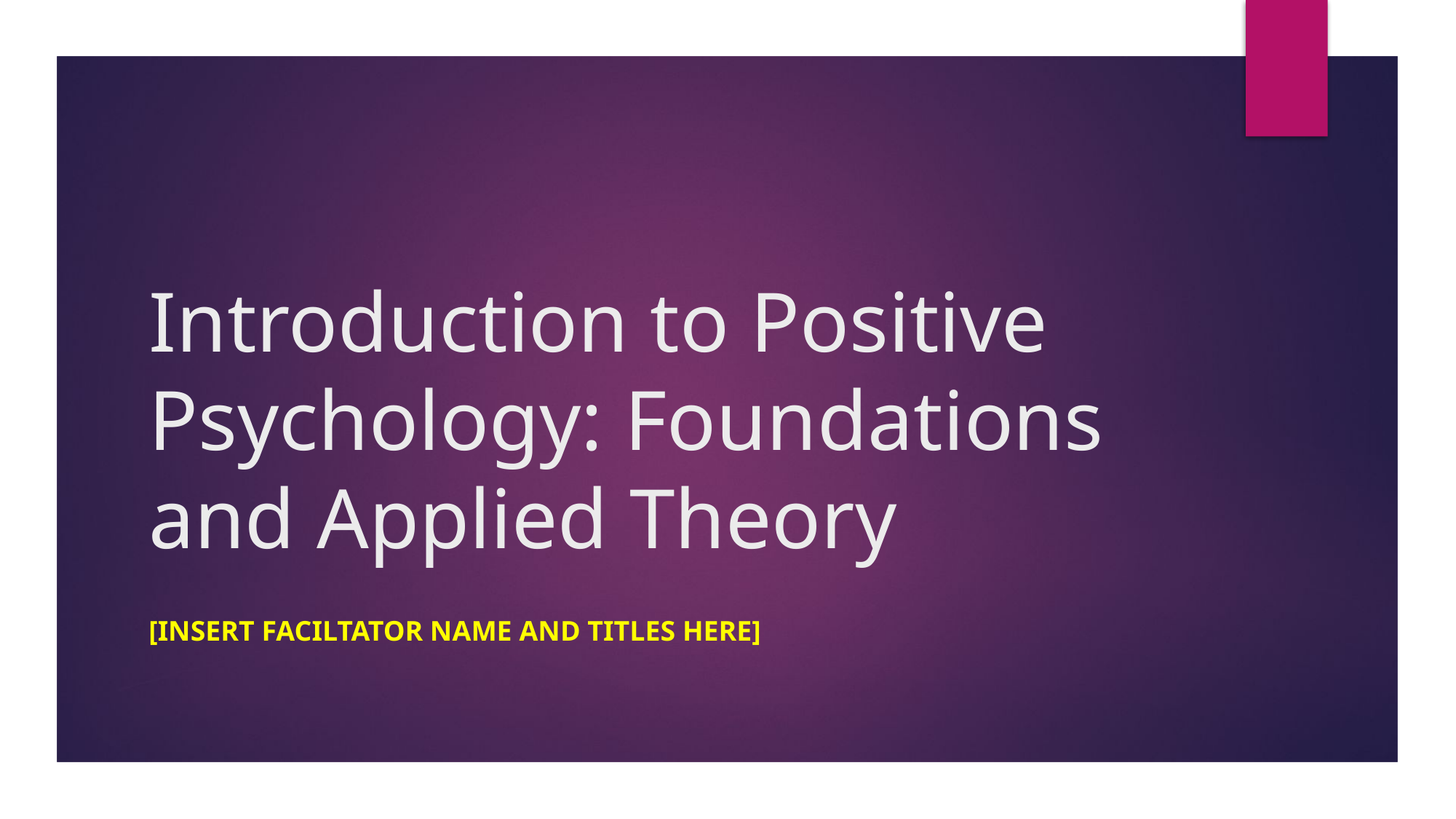

# Introduction to Positive Psychology: Foundations and Applied Theory
[Insert FACILTATOR NAME AND TITLES HERE]

## Slide 2
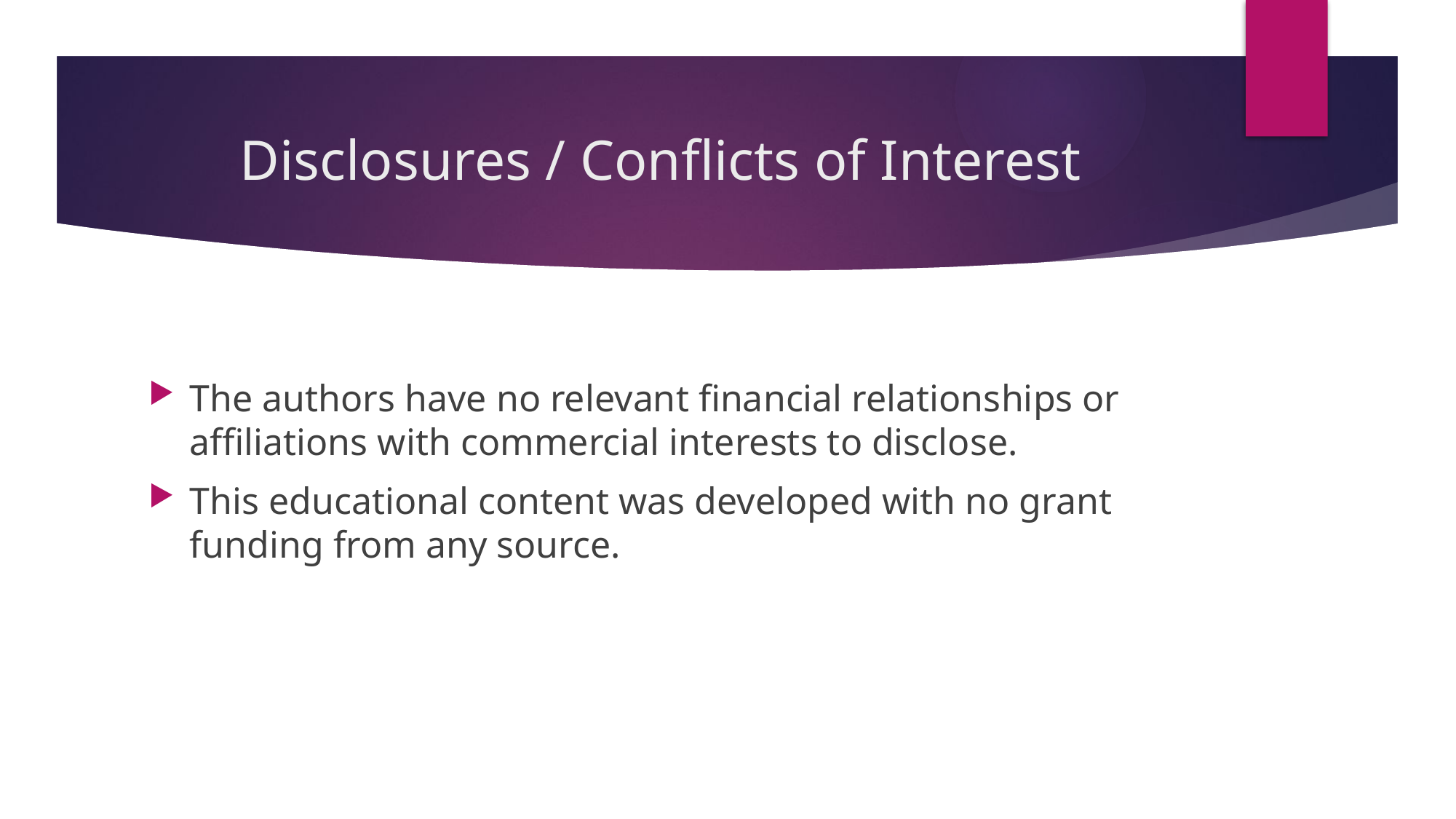

# Disclosures / Conflicts of Interest
The authors have no relevant financial relationships or affiliations with commercial interests to disclose.
This educational content was developed with no grant funding from any source.

## Slide 3
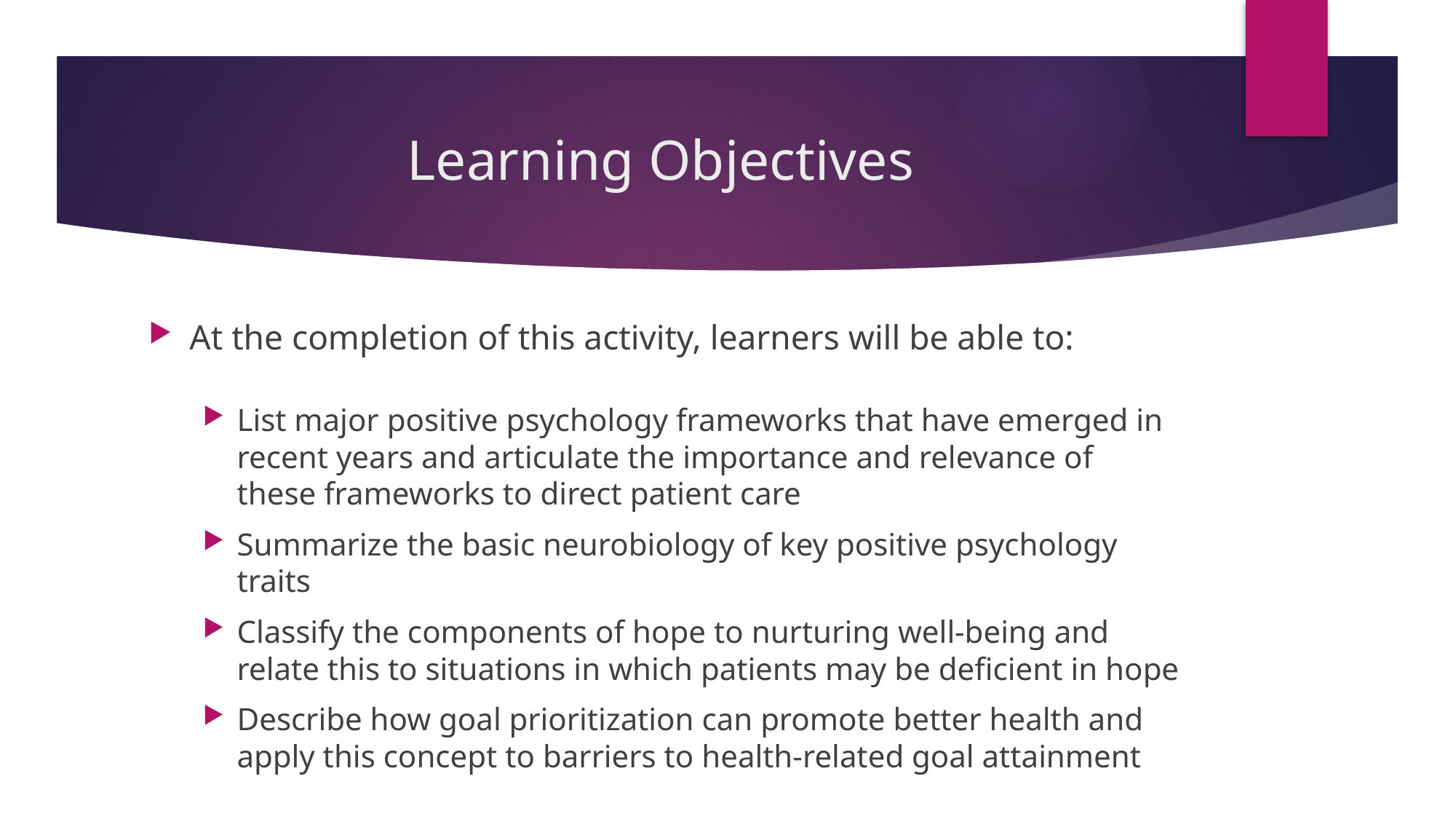

# Learning Objectives
At the completion of this activity, learners will be able to:
List major positive psychology frameworks that have emerged in recent years and articulate the importance and relevance of these frameworks to direct patient care
Summarize the basic neurobiology of key positive psychology traits
Classify the components of hope to nurturing well-being and relate this to situations in which patients may be deficient in hope
Describe how goal prioritization can promote better health and apply this concept to barriers to health-related goal attainment

## Slide 4
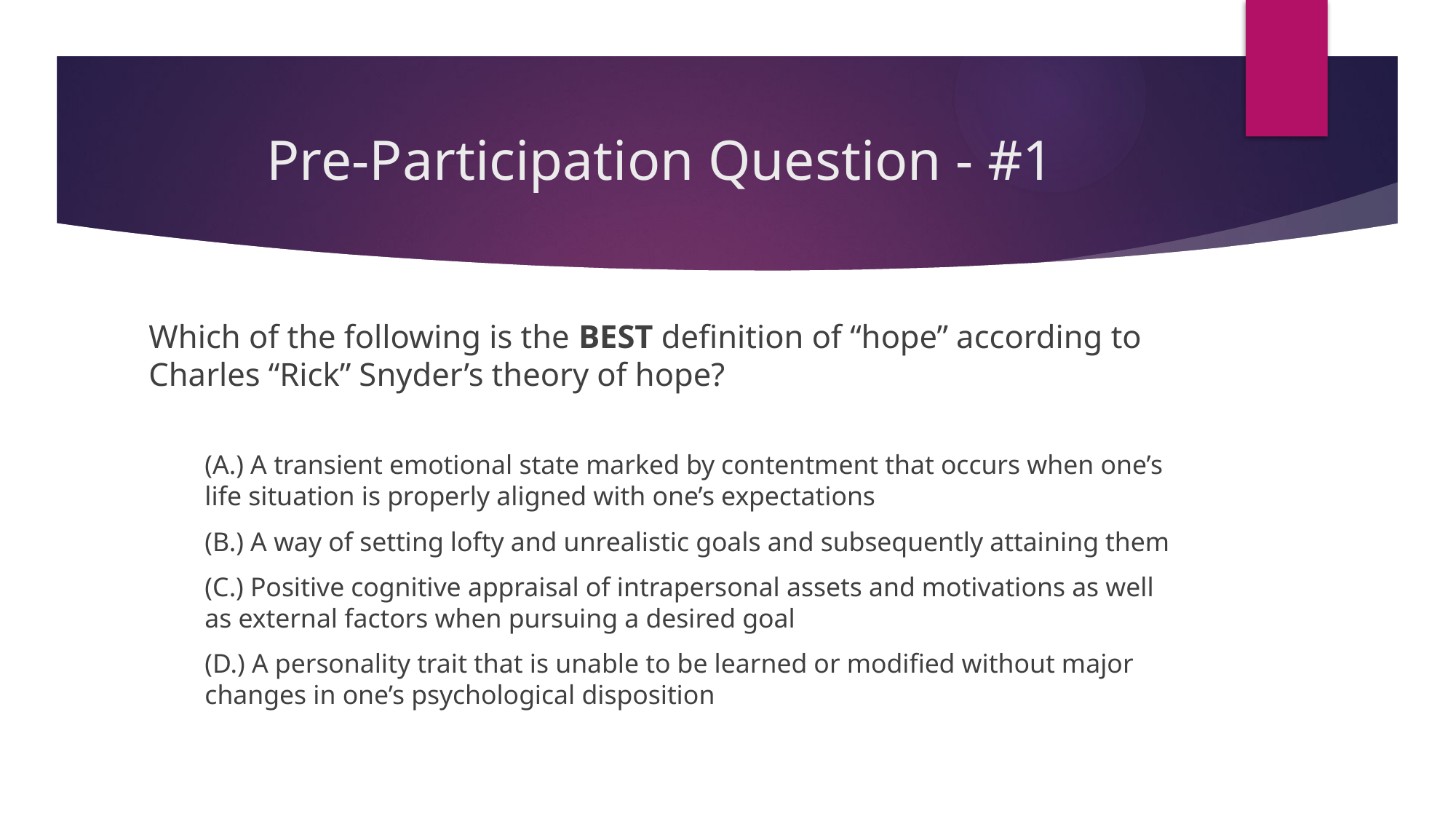

# Pre-Participation Question - #1
Which of the following is the BEST definition of “hope” according to Charles “Rick” Snyder’s theory of hope?
(A.) A transient emotional state marked by contentment that occurs when one’s life situation is properly aligned with one’s expectations
(B.) A way of setting lofty and unrealistic goals and subsequently attaining them
(C.) Positive cognitive appraisal of intrapersonal assets and motivations as well as external factors when pursuing a desired goal
(D.) A personality trait that is unable to be learned or modified without major changes in one’s psychological disposition

## Slide 5
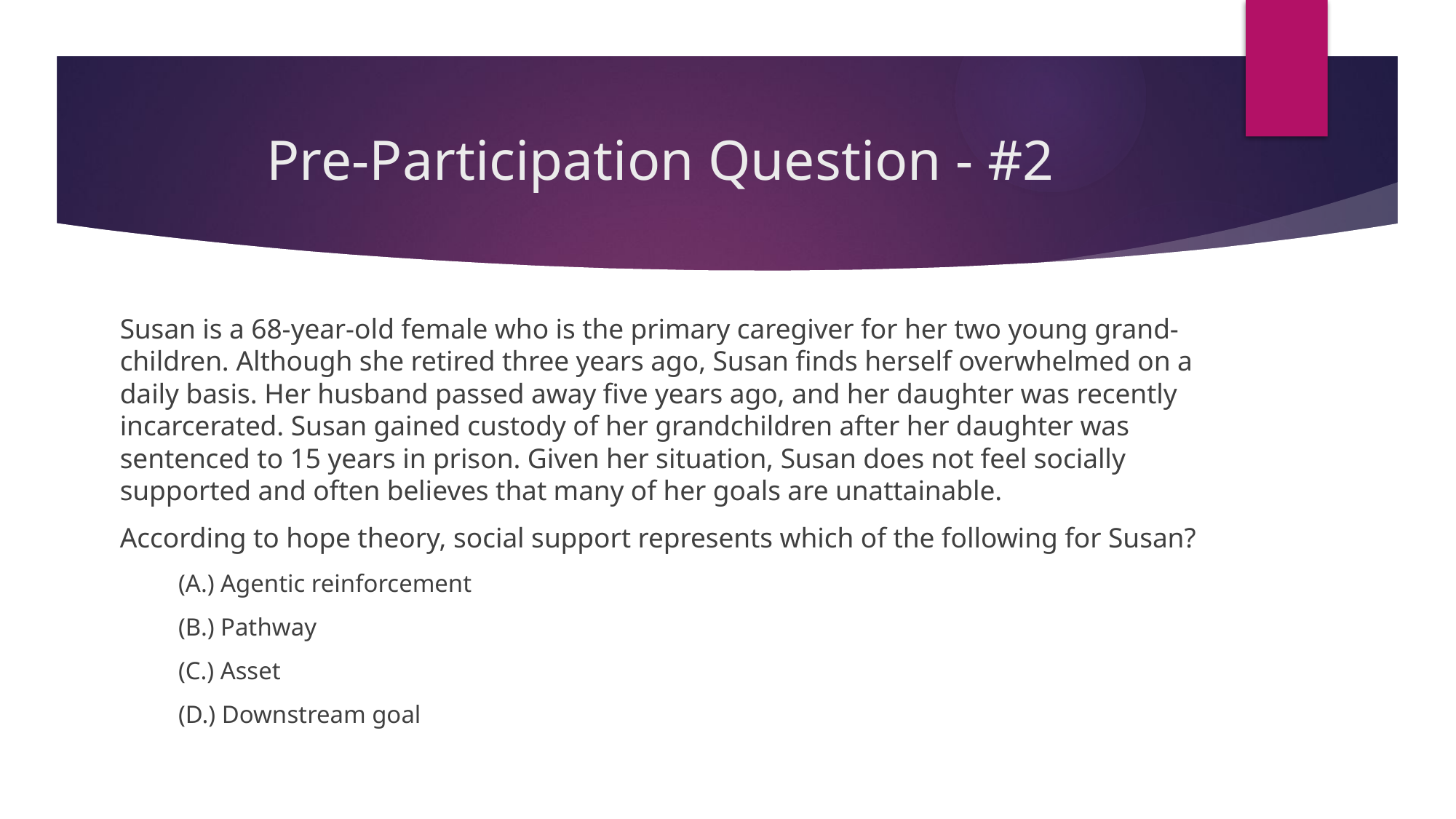

# Pre-Participation Question - #2
Susan is a 68-year-old female who is the primary caregiver for her two young grand-children. Although she retired three years ago, Susan finds herself overwhelmed on a daily basis. Her husband passed away five years ago, and her daughter was recently incarcerated. Susan gained custody of her grandchildren after her daughter was sentenced to 15 years in prison. Given her situation, Susan does not feel socially supported and often believes that many of her goals are unattainable.
According to hope theory, social support represents which of the following for Susan?
(A.) Agentic reinforcement
(B.) Pathway
(C.) Asset
(D.) Downstream goal

## Slide 6
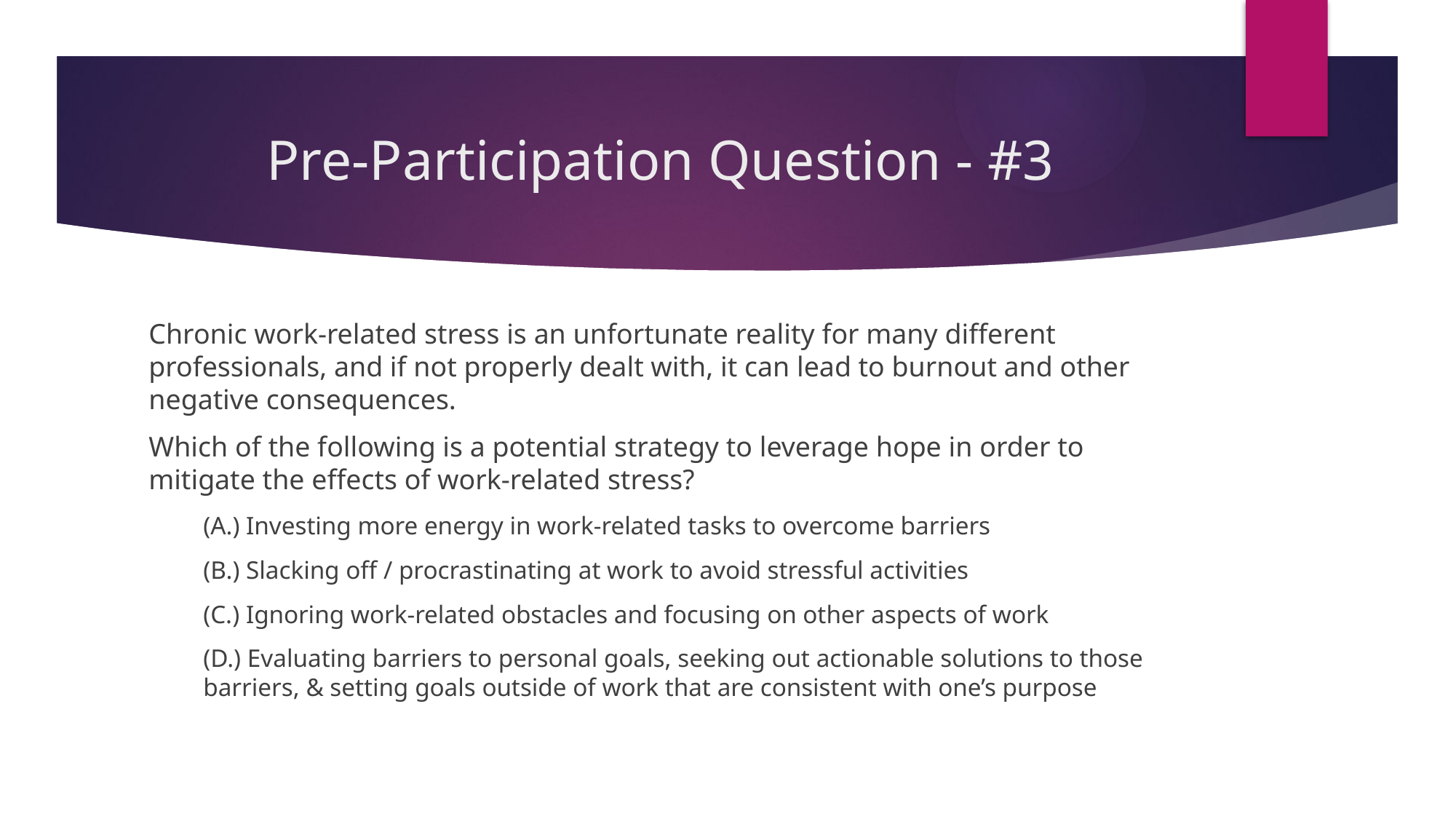

# Pre-Participation Question - #3
Chronic work-related stress is an unfortunate reality for many different professionals, and if not properly dealt with, it can lead to burnout and other negative consequences.
Which of the following is a potential strategy to leverage hope in order to mitigate the effects of work-related stress?
(A.) Investing more energy in work-related tasks to overcome barriers
(B.) Slacking off / procrastinating at work to avoid stressful activities
(C.) Ignoring work-related obstacles and focusing on other aspects of work
(D.) Evaluating barriers to personal goals, seeking out actionable solutions to those barriers, & setting goals outside of work that are consistent with one’s purpose

## Slide 7
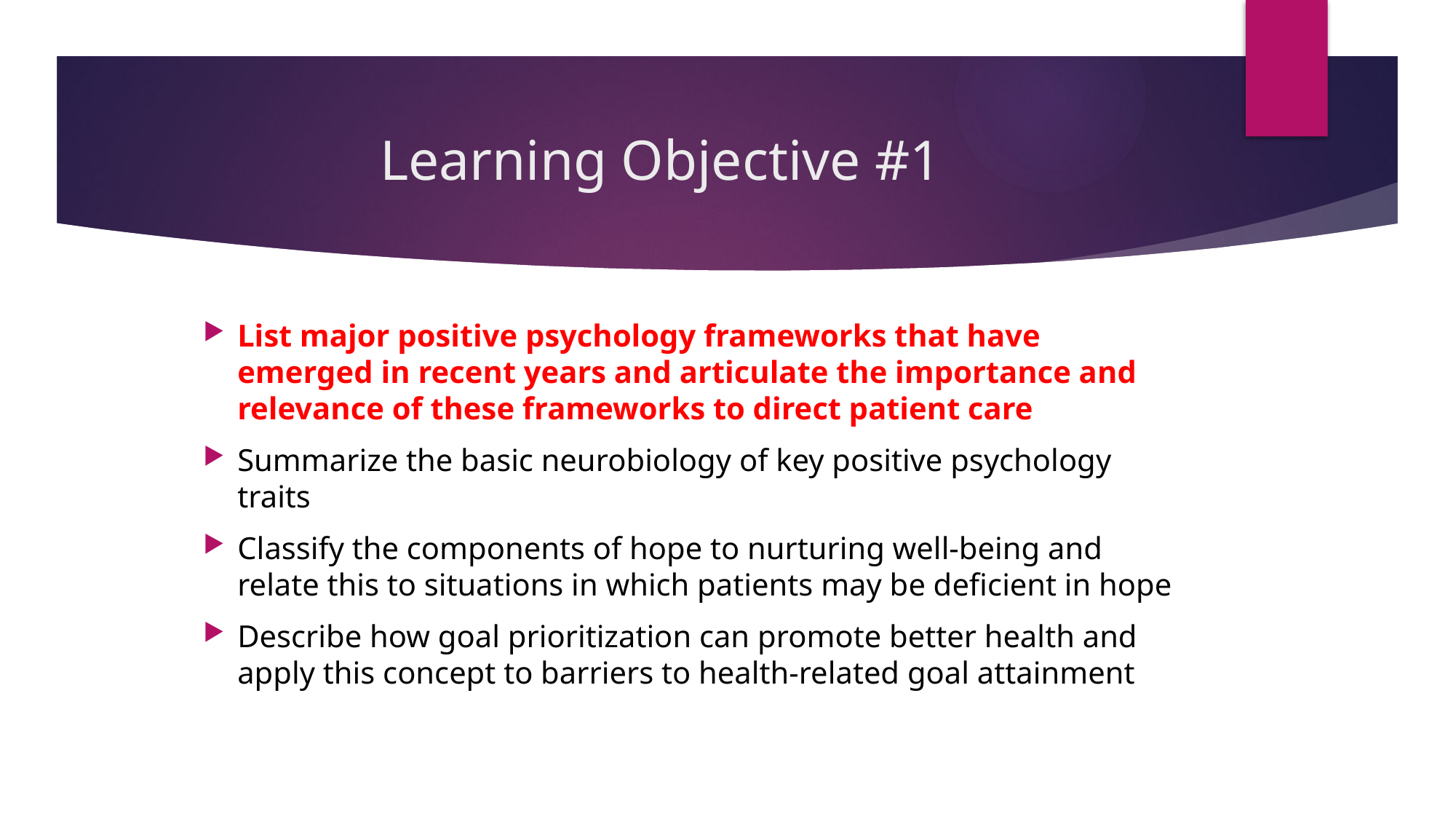

# Learning Objective #1
List major positive psychology frameworks that have emerged in recent years and articulate the importance and relevance of these frameworks to direct patient care
Summarize the basic neurobiology of key positive psychology traits
Classify the components of hope to nurturing well-being and relate this to situations in which patients may be deficient in hope
Describe how goal prioritization can promote better health and apply this concept to barriers to health-related goal attainment

## Slide 8
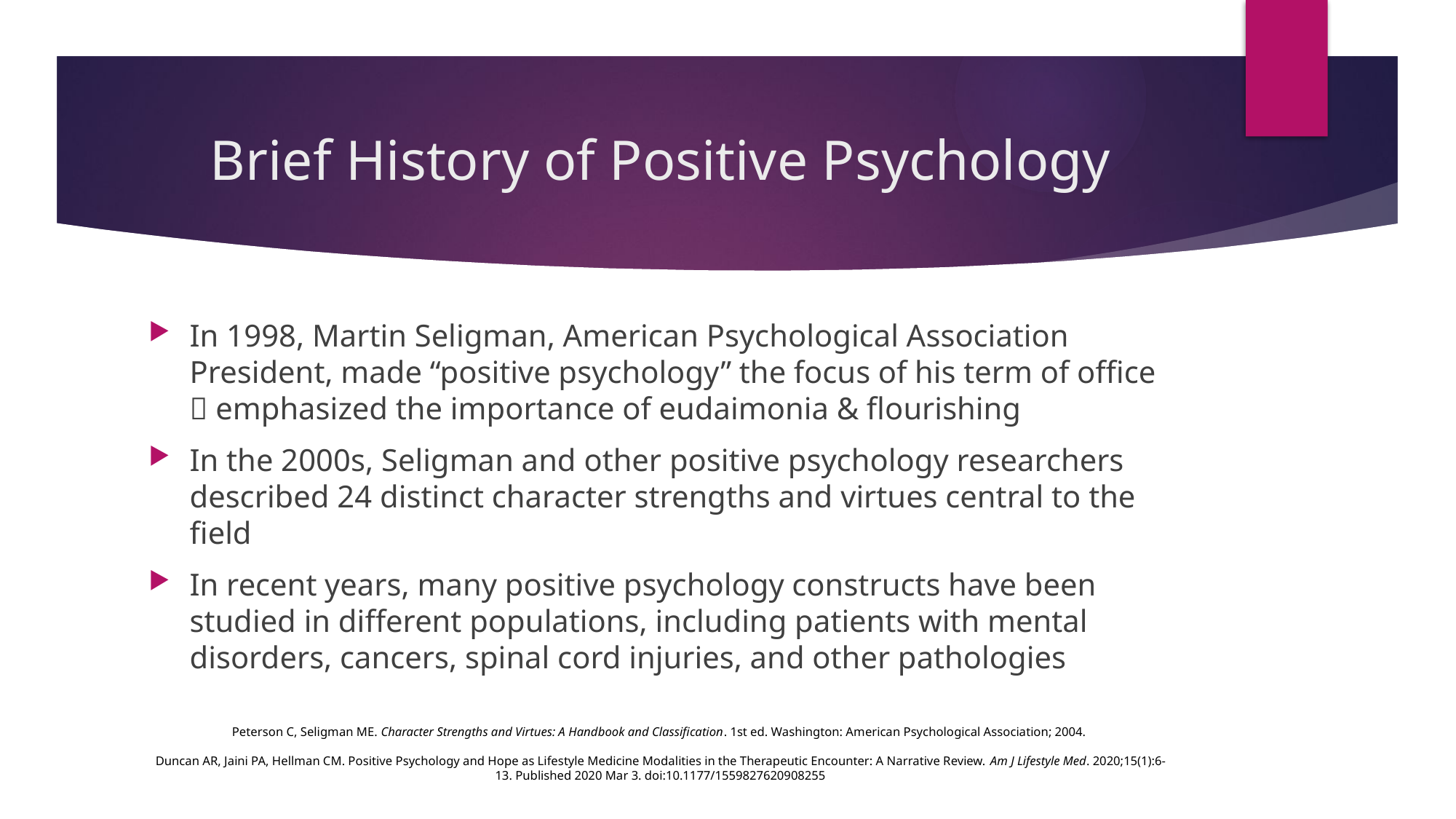

# Brief History of Positive Psychology
In 1998, Martin Seligman, American Psychological Association President, made “positive psychology” the focus of his term of office  emphasized the importance of eudaimonia & flourishing
In the 2000s, Seligman and other positive psychology researchers described 24 distinct character strengths and virtues central to the field
In recent years, many positive psychology constructs have been studied in different populations, including patients with mental disorders, cancers, spinal cord injuries, and other pathologies
Peterson C, Seligman ME. Character Strengths and Virtues: A Handbook and Classification. 1st ed. Washington: American Psychological Association; 2004.
Duncan AR, Jaini PA, Hellman CM. Positive Psychology and Hope as Lifestyle Medicine Modalities in the Therapeutic Encounter: A Narrative Review. Am J Lifestyle Med. 2020;15(1):6-13. Published 2020 Mar 3. doi:10.1177/1559827620908255

## Slide 9
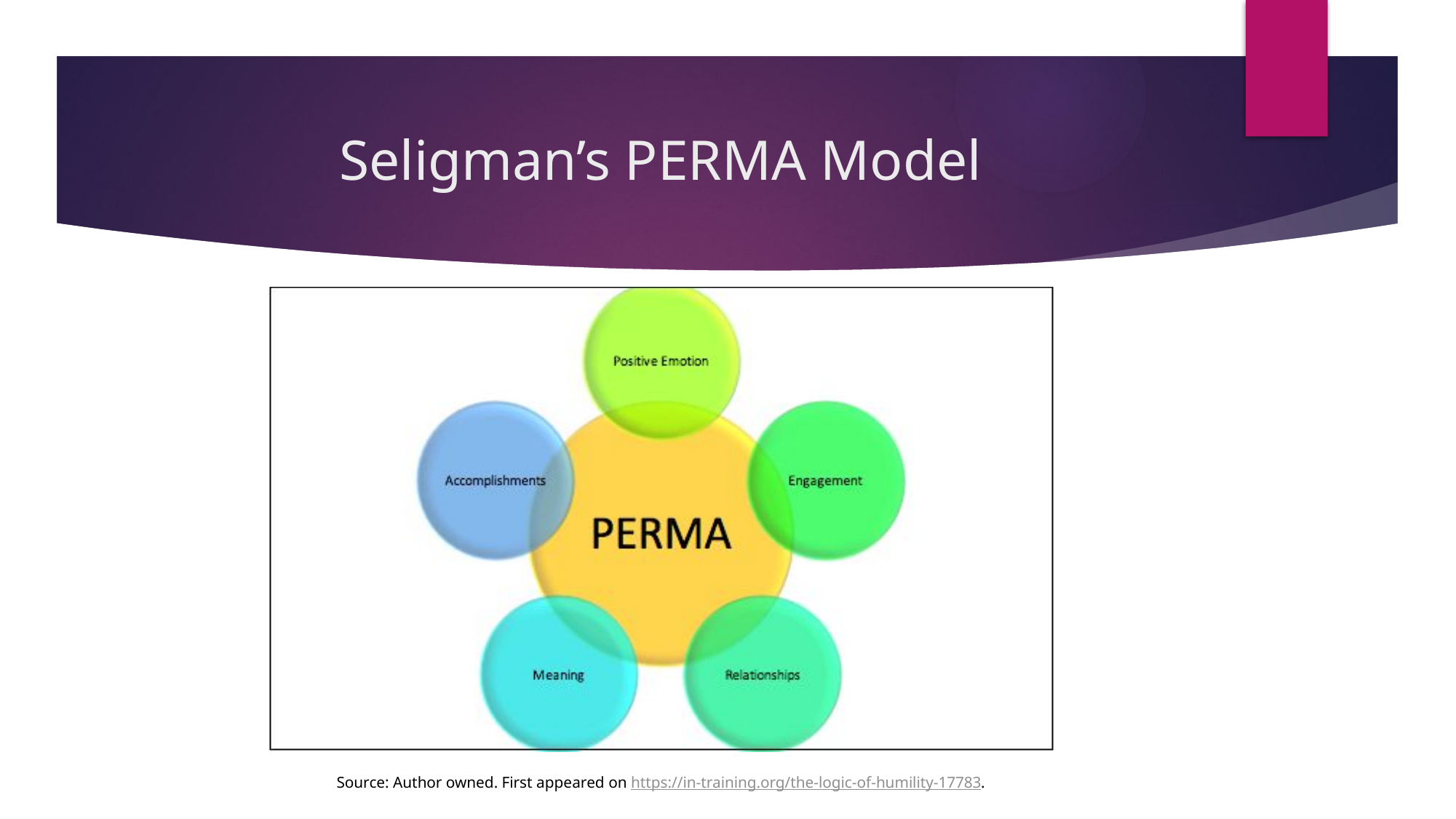

# Seligman’s PERMA Model
Source: Author owned. First appeared on https://in-training.org/the-logic-of-humility-17783.

## Slide 10
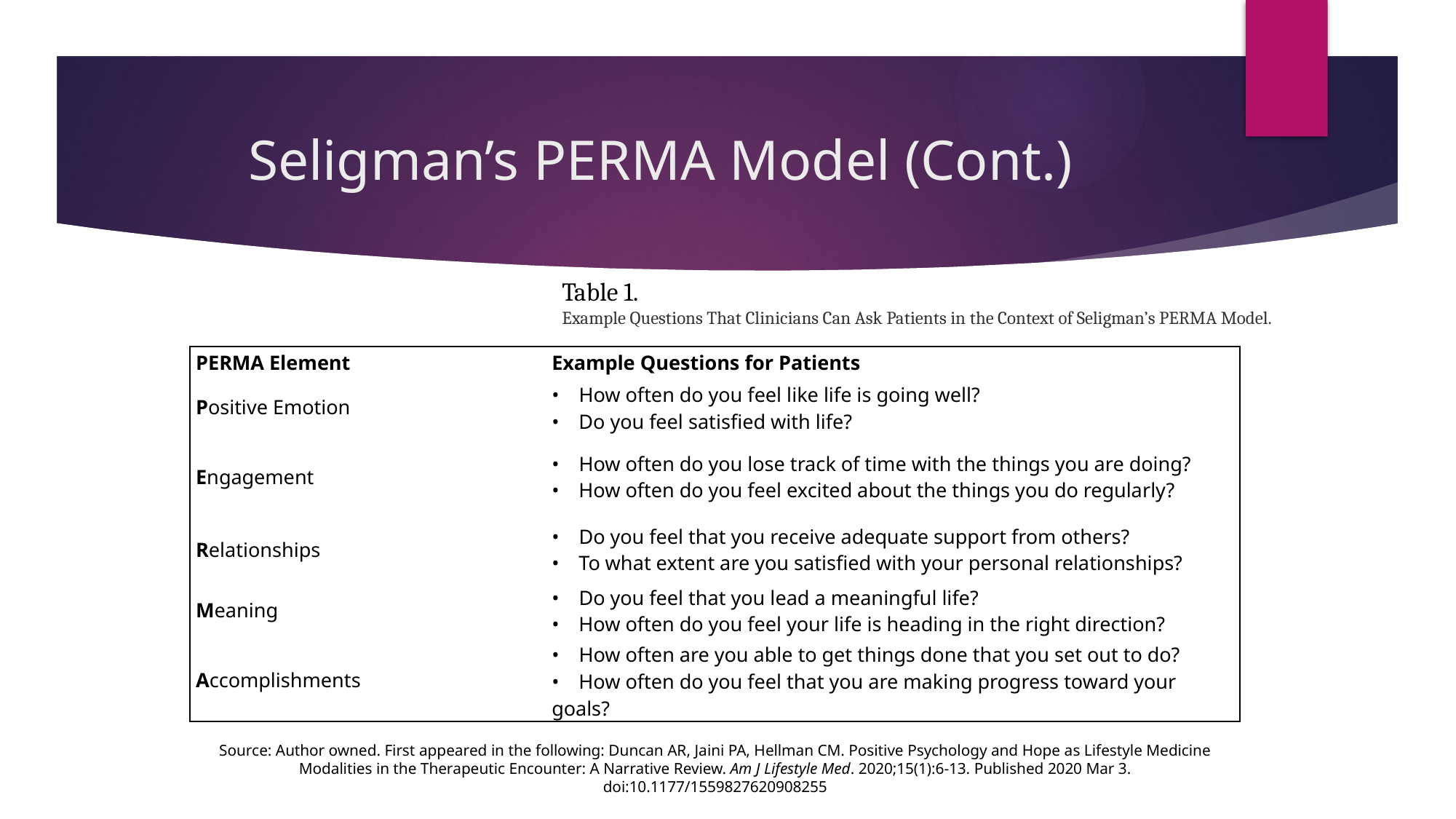

# Seligman’s PERMA Model (Cont.)
Table 1.
Example Questions That Clinicians Can Ask Patients in the Context of Seligman’s PERMA Model.
| PERMA Element | Example Questions for Patients |
| --- | --- |
| Positive Emotion | • How often do you feel like life is going well? • Do you feel satisfied with life? |
| Engagement | • How often do you lose track of time with the things you are doing? • How often do you feel excited about the things you do regularly? |
| Relationships | • Do you feel that you receive adequate support from others? • To what extent are you satisfied with your personal relationships? |
| Meaning | • Do you feel that you lead a meaningful life? • How often do you feel your life is heading in the right direction? |
| Accomplishments | • How often are you able to get things done that you set out to do? • How often do you feel that you are making progress toward your goals? |
Source: Author owned. First appeared in the following: Duncan AR, Jaini PA, Hellman CM. Positive Psychology and Hope as Lifestyle Medicine Modalities in the Therapeutic Encounter: A Narrative Review. Am J Lifestyle Med. 2020;15(1):6-13. Published 2020 Mar 3. doi:10.1177/1559827620908255

## Slide 11
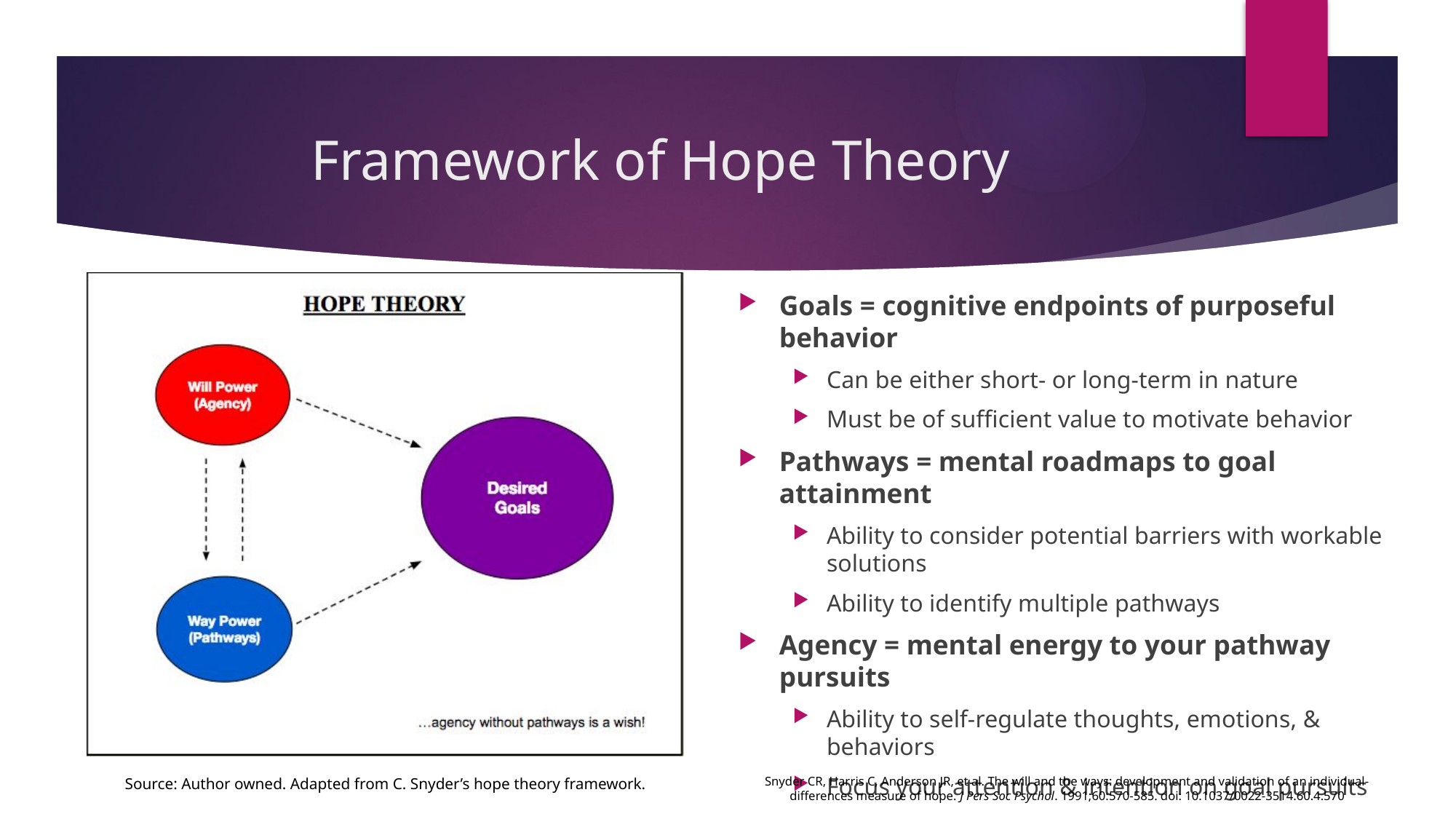

# Framework of Hope Theory
Goals = cognitive endpoints of purposeful behavior
Can be either short- or long-term in nature
Must be of sufficient value to motivate behavior
Pathways = mental roadmaps to goal attainment
Ability to consider potential barriers with workable solutions
Ability to identify multiple pathways
Agency = mental energy to your pathway pursuits
Ability to self-regulate thoughts, emotions, & behaviors
Focus your attention & intention on goal pursuits
Source: Author owned. Adapted from C. Snyder’s hope theory framework.
Snyder CR, Harris C, Anderson JR, et al. The will and the ways: development and validation of an individual-differences measure of hope. J Pers Soc Psychol. 1991;60:570-585. doi: 10.1037/0022-3514.60.4.570
…agency without pathways is a wish!

## Slide 12
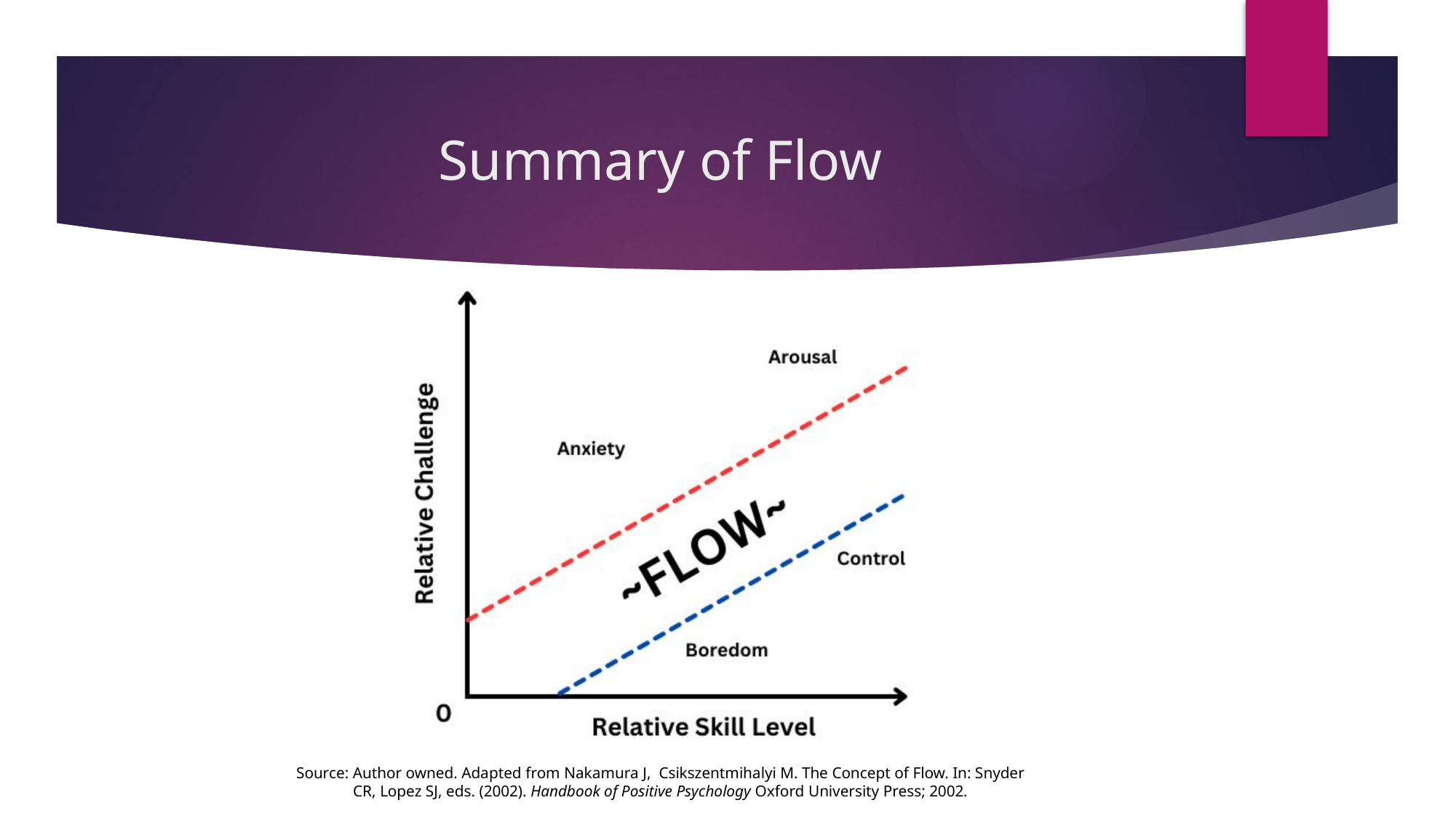

# Summary of Flow
Source: Author owned. Adapted from Nakamura J, Csikszentmihalyi M. The Concept of Flow. In: Snyder CR, Lopez SJ, eds. (2002). Handbook of Positive Psychology Oxford University Press; 2002.

## Slide 13
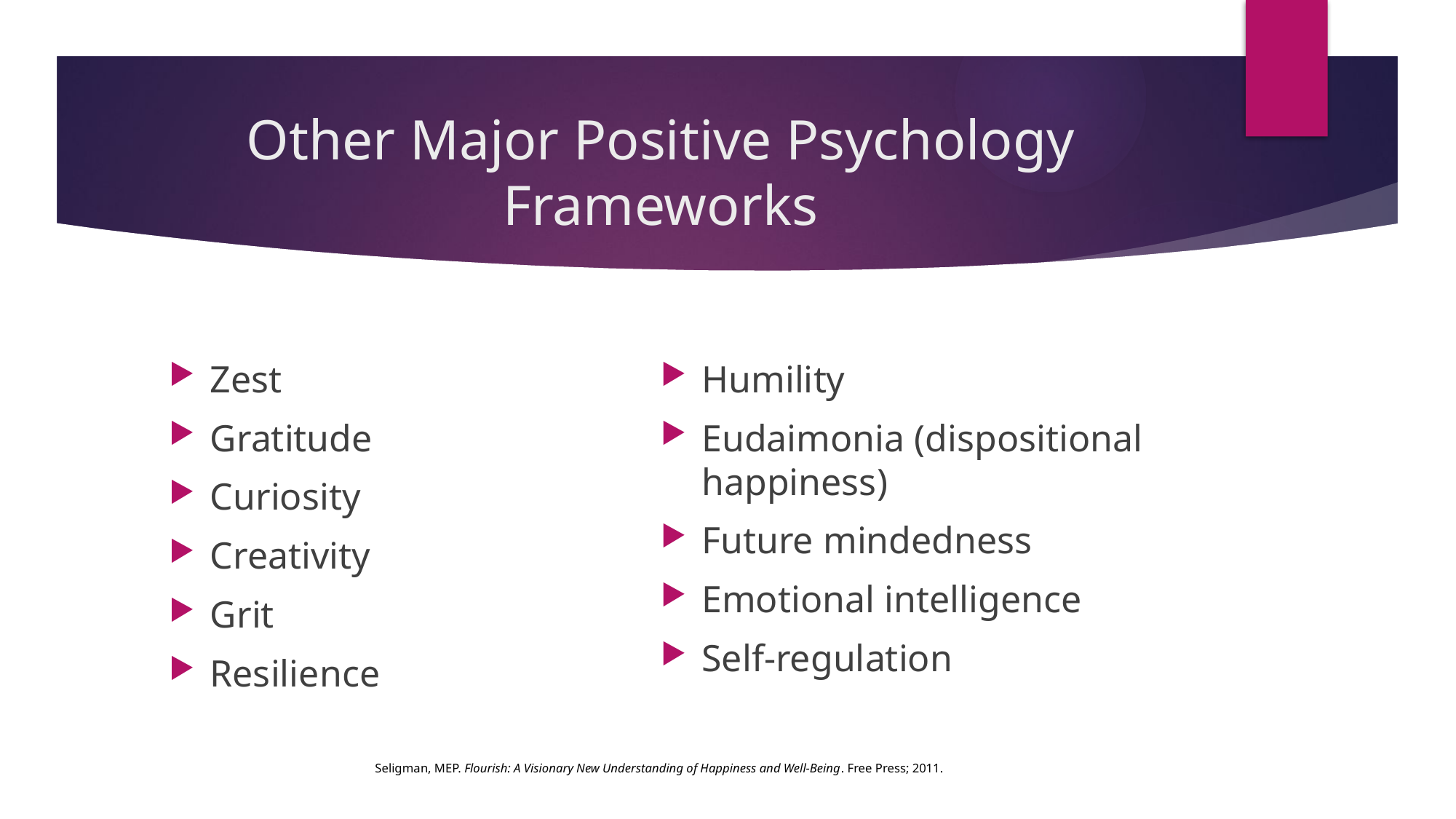

# Other Major Positive Psychology Frameworks
Zest
Gratitude
Curiosity
Creativity
Grit
Resilience
Humility
Eudaimonia (dispositional happiness)
Future mindedness
Emotional intelligence
Self-regulation
Seligman, MEP. Flourish: A Visionary New Understanding of Happiness and Well-Being. Free Press; 2011.

## Slide 14
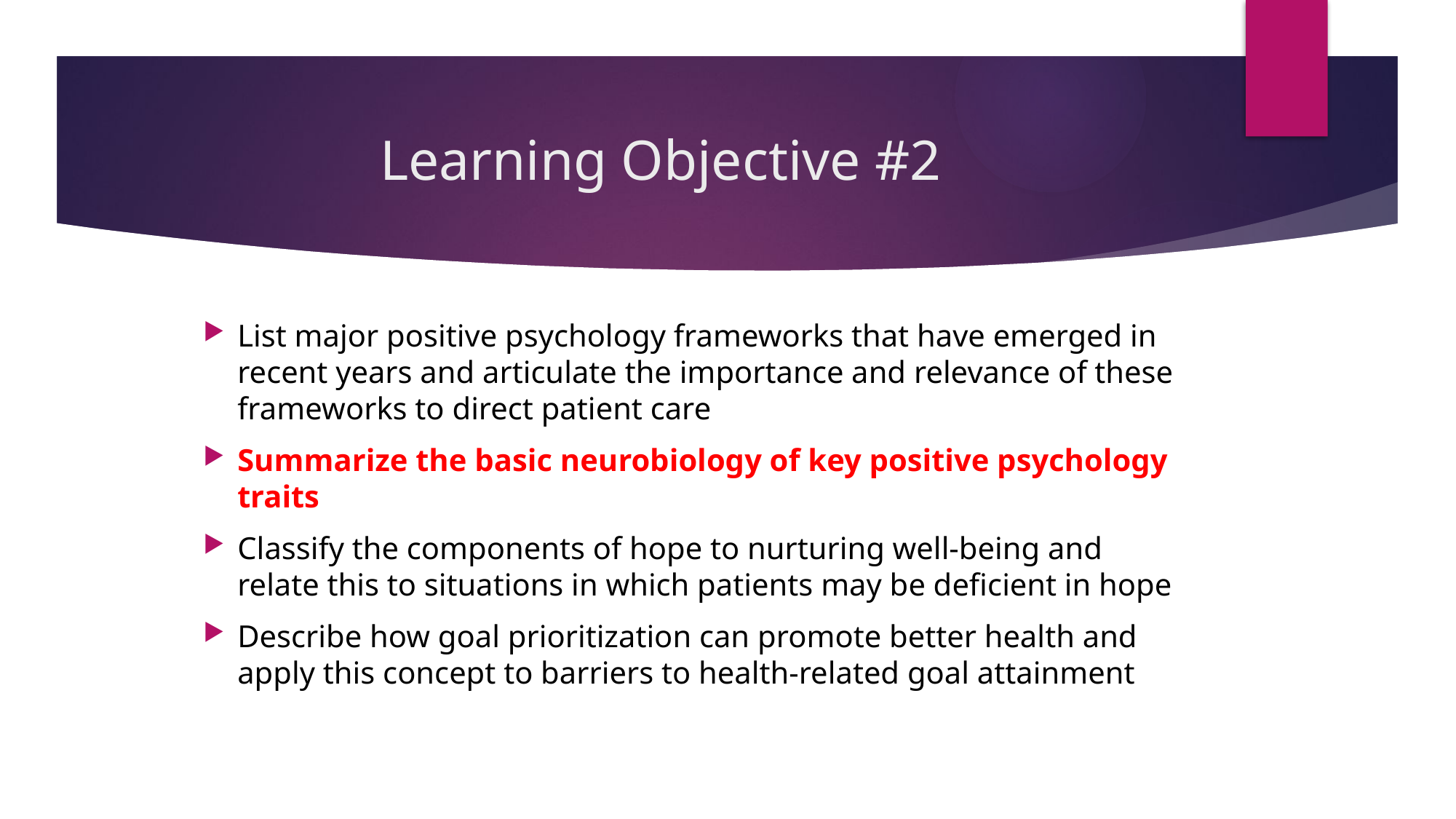

# Learning Objective #2
List major positive psychology frameworks that have emerged in recent years and articulate the importance and relevance of these frameworks to direct patient care
Summarize the basic neurobiology of key positive psychology traits
Classify the components of hope to nurturing well-being and relate this to situations in which patients may be deficient in hope
Describe how goal prioritization can promote better health and apply this concept to barriers to health-related goal attainment

## Slide 15
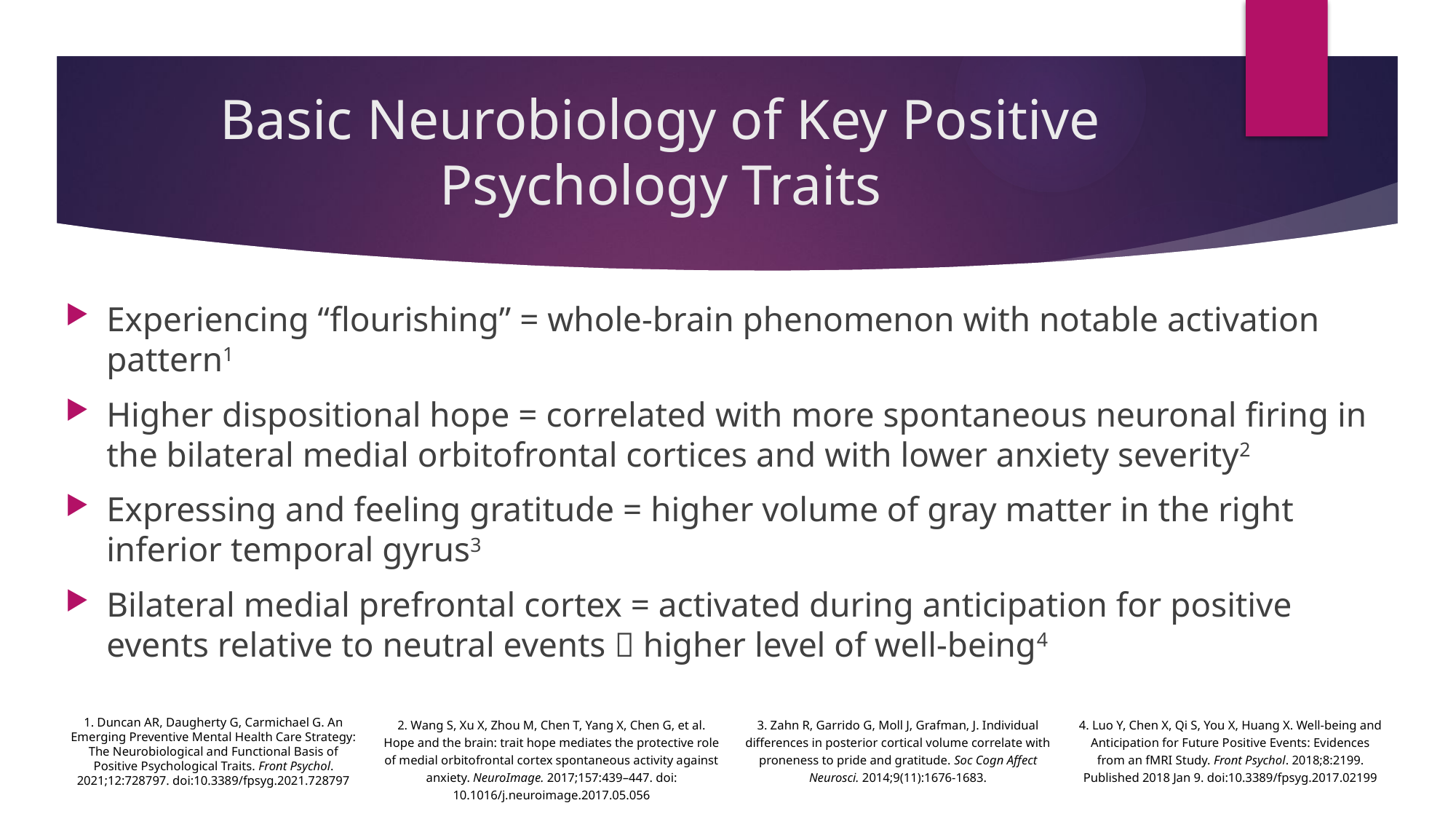

# Basic Neurobiology of Key Positive Psychology Traits
Experiencing “flourishing” = whole-brain phenomenon with notable activation pattern1
Higher dispositional hope = correlated with more spontaneous neuronal firing in the bilateral medial orbitofrontal cortices and with lower anxiety severity2
Expressing and feeling gratitude = higher volume of gray matter in the right inferior temporal gyrus3
Bilateral medial prefrontal cortex = activated during anticipation for positive events relative to neutral events  higher level of well-being4
1. Duncan AR, Daugherty G, Carmichael G. An Emerging Preventive Mental Health Care Strategy: The Neurobiological and Functional Basis of Positive Psychological Traits. Front Psychol. 2021;12:728797. doi:10.3389/fpsyg.2021.728797
2. Wang S, Xu X, Zhou M, Chen T, Yang X, Chen G, et al. Hope and the brain: trait hope mediates the protective role of medial orbitofrontal cortex spontaneous activity against anxiety. NeuroImage. 2017;157:439–447. doi: 10.1016/j.neuroimage.2017.05.056
3. Zahn R, Garrido G, Moll J, Grafman, J. Individual differences in posterior cortical volume correlate with proneness to pride and gratitude. Soc Cogn Affect Neurosci. 2014;9(11):1676-1683.
4. Luo Y, Chen X, Qi S, You X, Huang X. Well-being and Anticipation for Future Positive Events: Evidences from an fMRI Study. Front Psychol. 2018;8:2199. Published 2018 Jan 9. doi:10.3389/fpsyg.2017.02199

## Slide 16
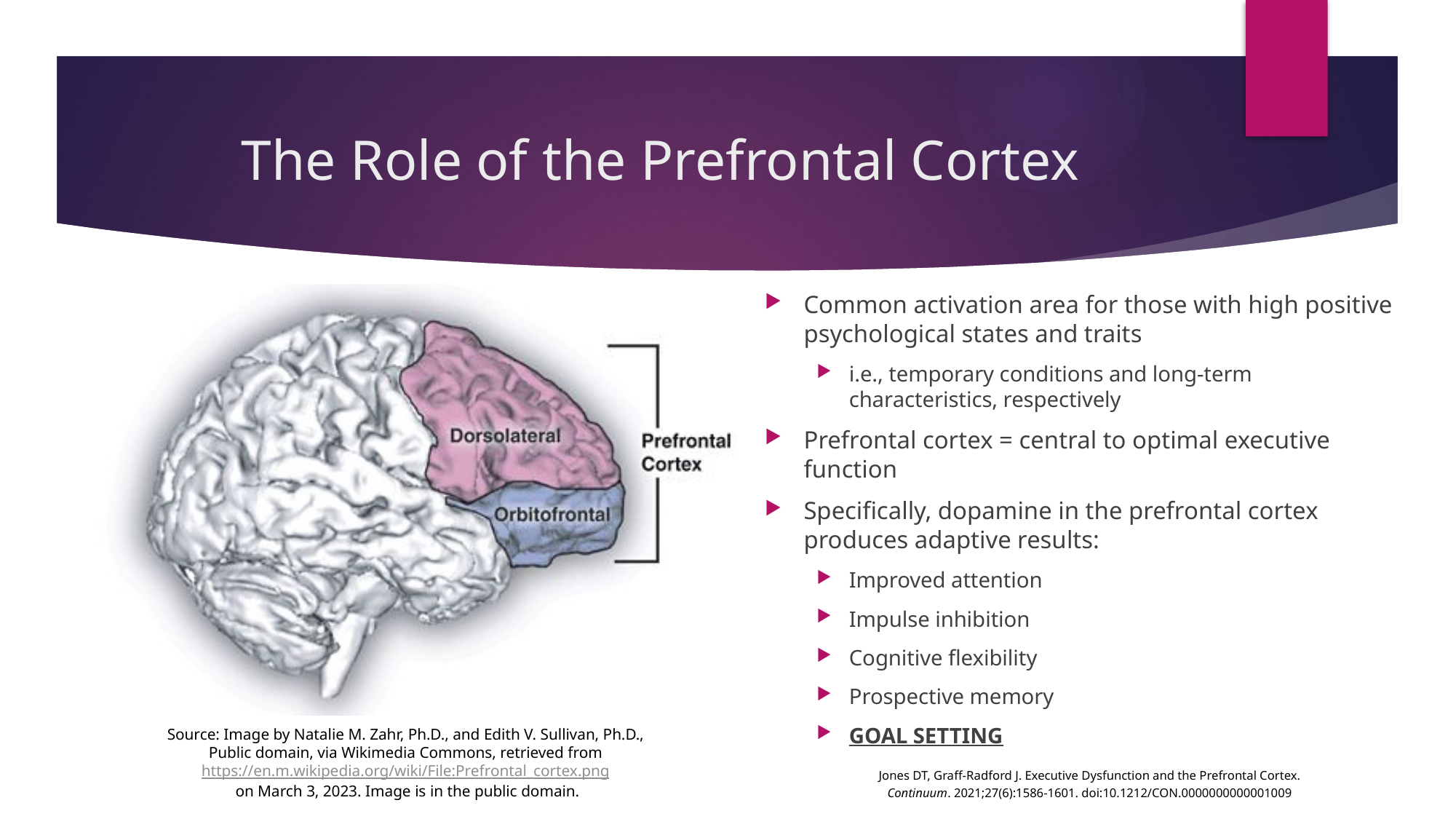

# The Role of the Prefrontal Cortex
Common activation area for those with high positive psychological states and traits
i.e., temporary conditions and long-term characteristics, respectively
Prefrontal cortex = central to optimal executive function
Specifically, dopamine in the prefrontal cortex produces adaptive results:
Improved attention
Impulse inhibition
Cognitive flexibility
Prospective memory
GOAL SETTING
Source: Image by Natalie M. Zahr, Ph.D., and Edith V. Sullivan, Ph.D.,
Public domain, via Wikimedia Commons, retrieved from https://en.m.wikipedia.org/wiki/File:Prefrontal_cortex.png
on March 3, 2023. Image is in the public domain.
Jones DT, Graff-Radford J. Executive Dysfunction and the Prefrontal Cortex. Continuum. 2021;27(6):1586-1601. doi:10.1212/CON.0000000000001009

## Slide 17
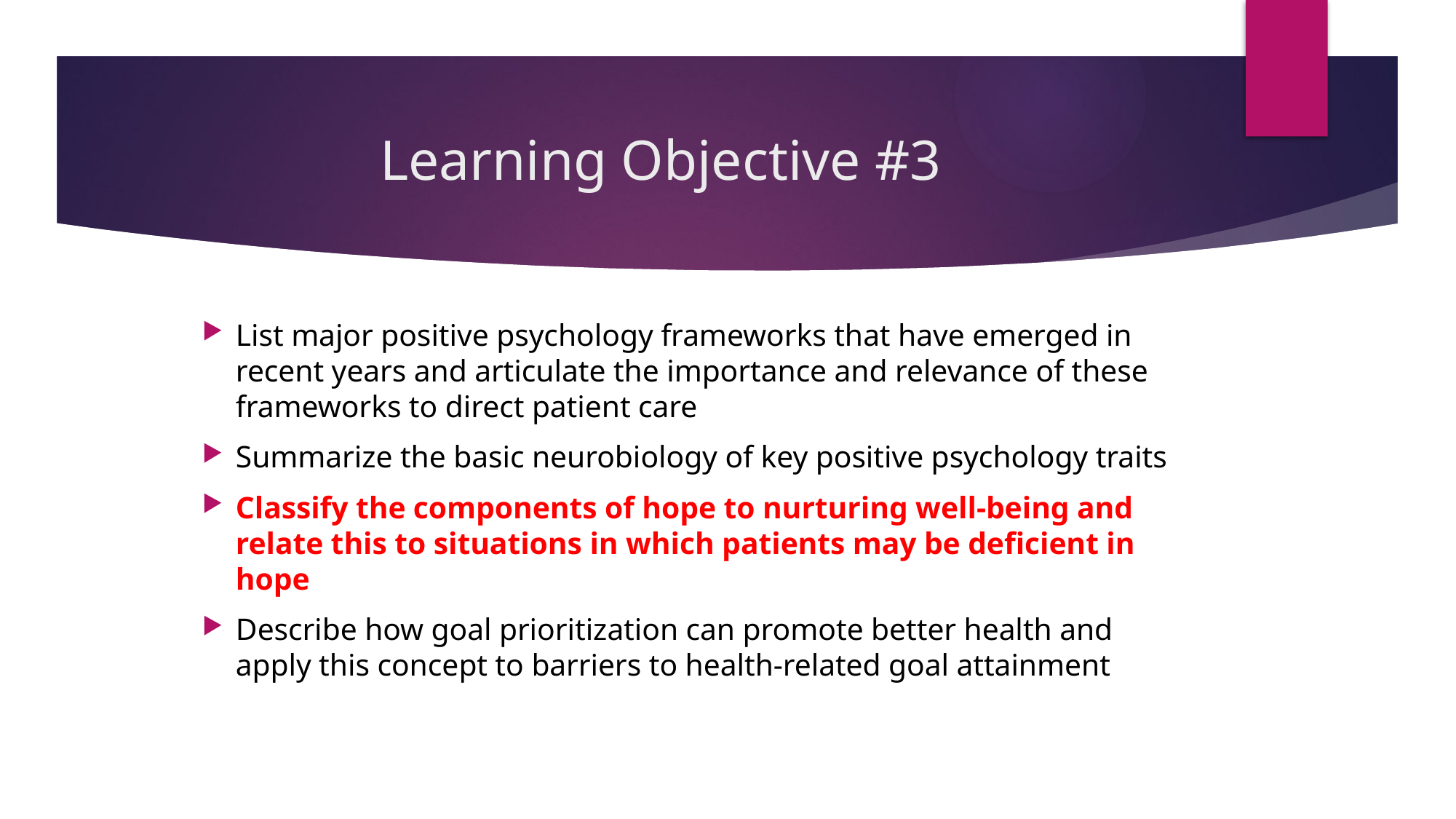

# Learning Objective #3
List major positive psychology frameworks that have emerged in recent years and articulate the importance and relevance of these frameworks to direct patient care
Summarize the basic neurobiology of key positive psychology traits
Classify the components of hope to nurturing well-being and relate this to situations in which patients may be deficient in hope
Describe how goal prioritization can promote better health and apply this concept to barriers to health-related goal attainment

## Slide 18
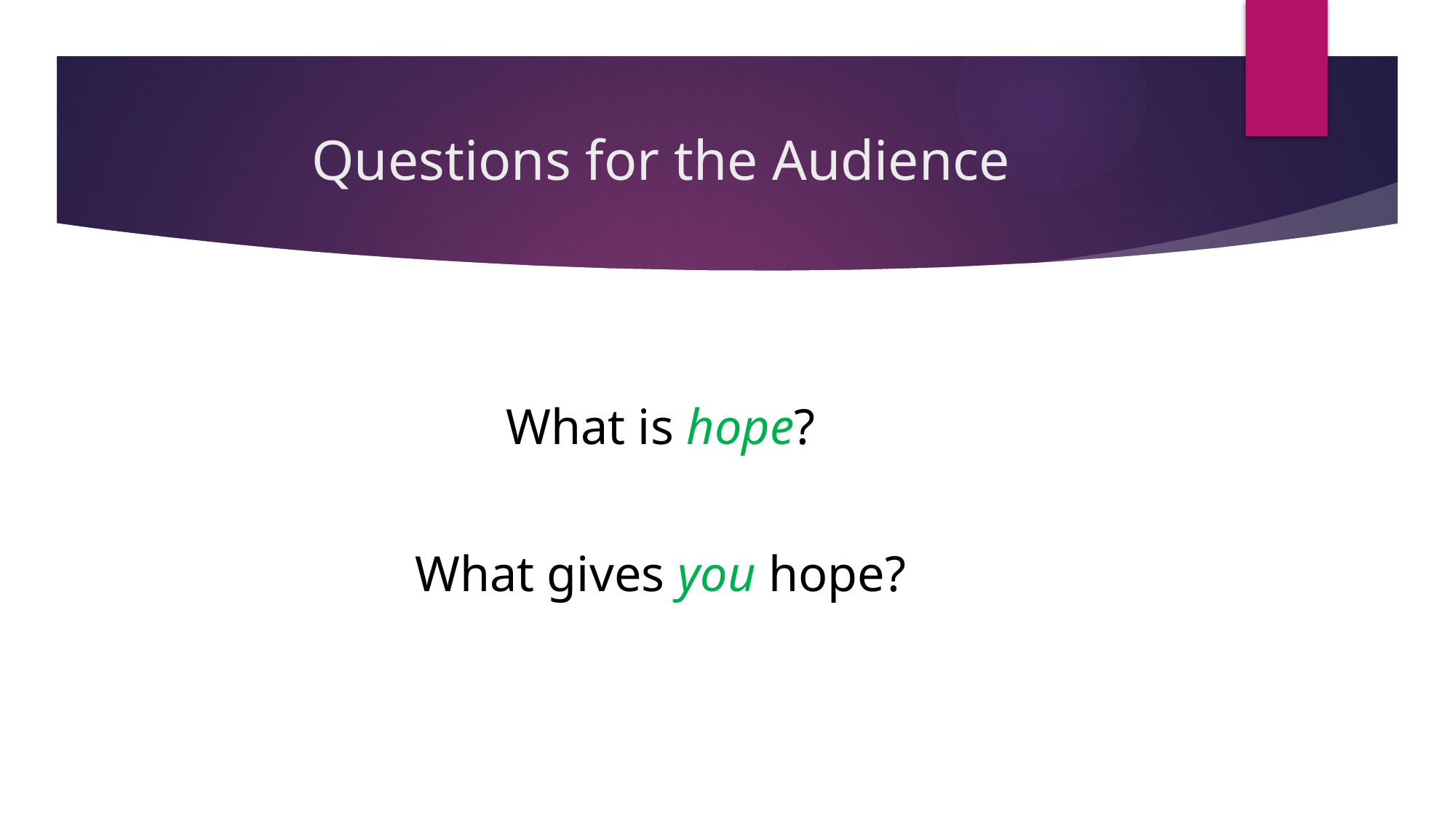

# Questions for the Audience
What is hope?
What gives you hope?

## Slide 19
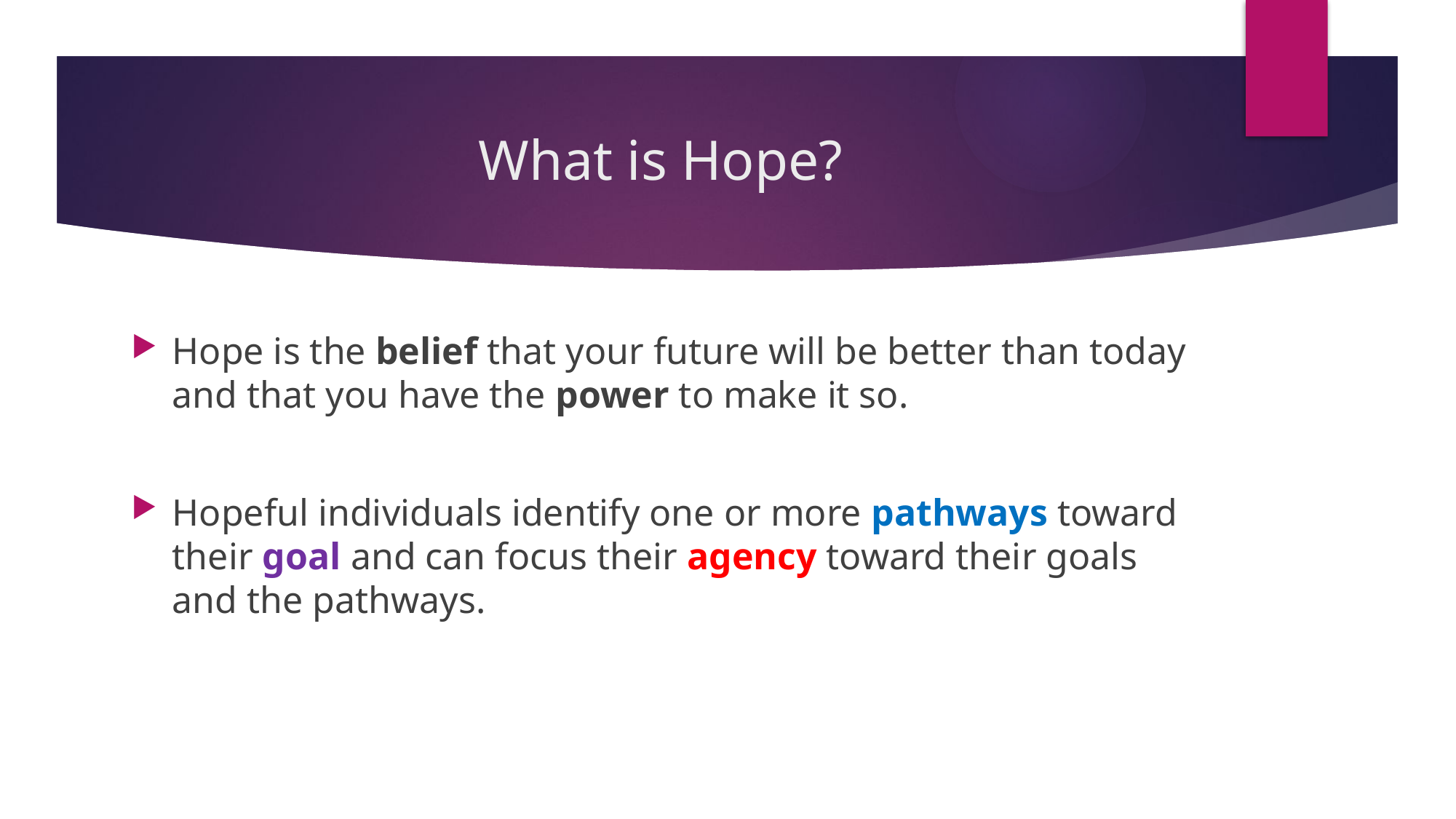

# What is Hope?
Hope is the belief that your future will be better than today and that you have the power to make it so.
Hopeful individuals identify one or more pathways toward their goal and can focus their agency toward their goals and the pathways.

## Slide 20
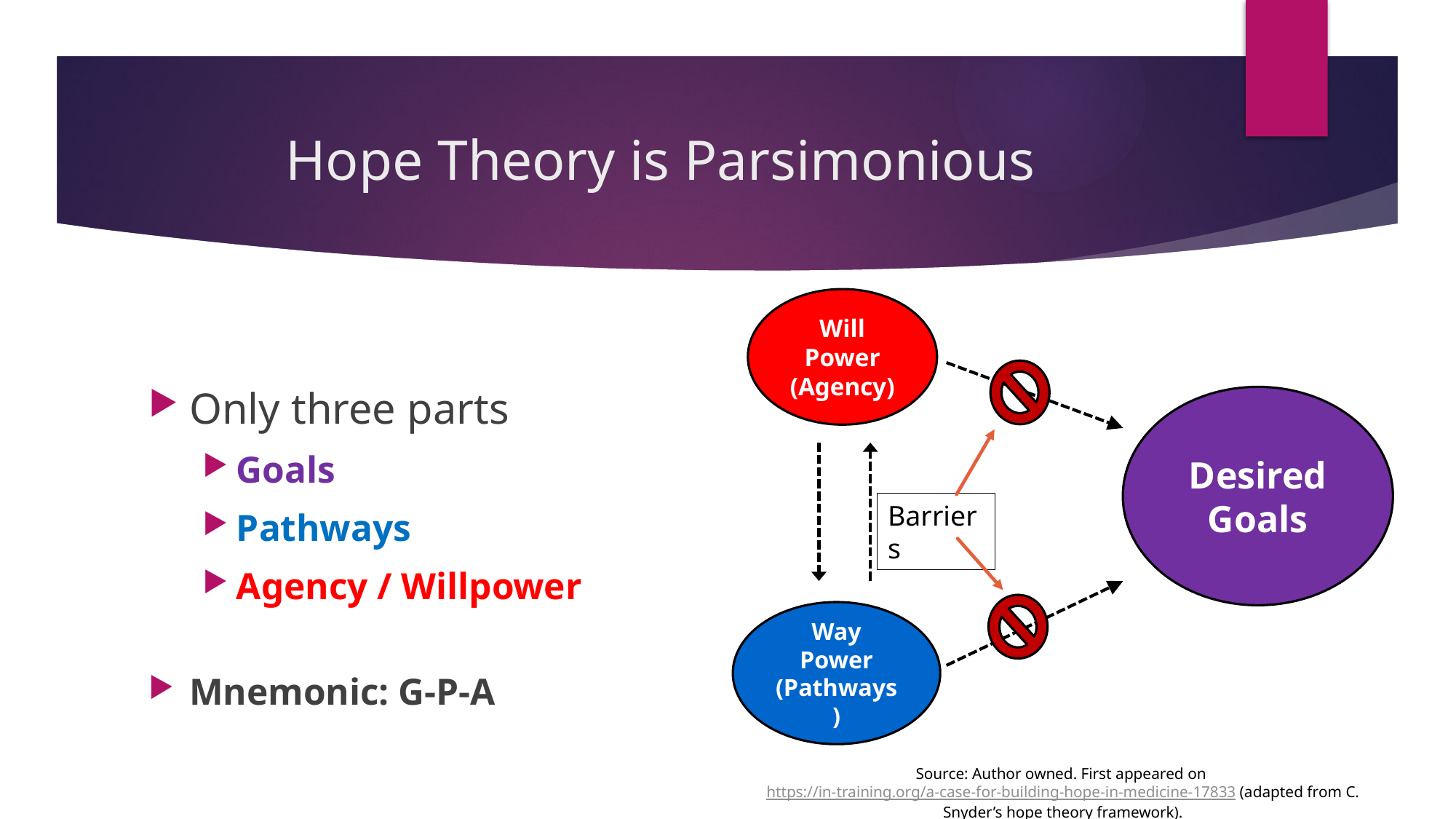

# Hope Theory is Parsimonious
Will Power
(Agency)
Desired
Goals
Way Power
(Pathways)
Only three parts
Goals
Pathways
Agency / Willpower
Mnemonic: G-P-A
Barriers
Source: Author owned. First appeared on https://in-training.org/a-case-for-building-hope-in-medicine-17833 (adapted from C. Snyder’s hope theory framework).

## Slide 21
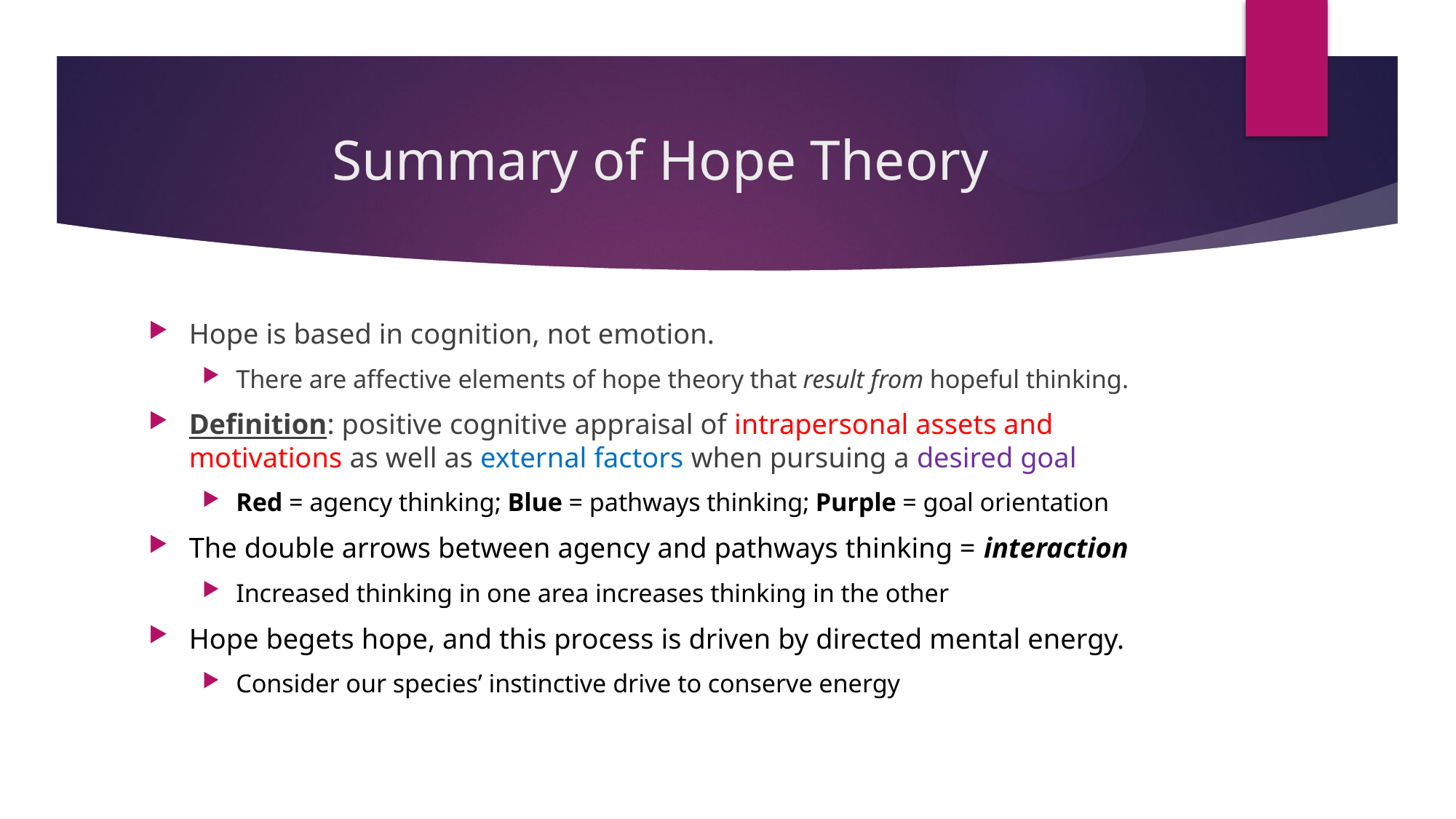

# Summary of Hope Theory
Hope is based in cognition, not emotion.
There are affective elements of hope theory that result from hopeful thinking.
Definition: positive cognitive appraisal of intrapersonal assets and motivations as well as external factors when pursuing a desired goal
Red = agency thinking; Blue = pathways thinking; Purple = goal orientation
The double arrows between agency and pathways thinking = interaction
Increased thinking in one area increases thinking in the other
Hope begets hope, and this process is driven by directed mental energy.
Consider our species’ instinctive drive to conserve energy

## Slide 22
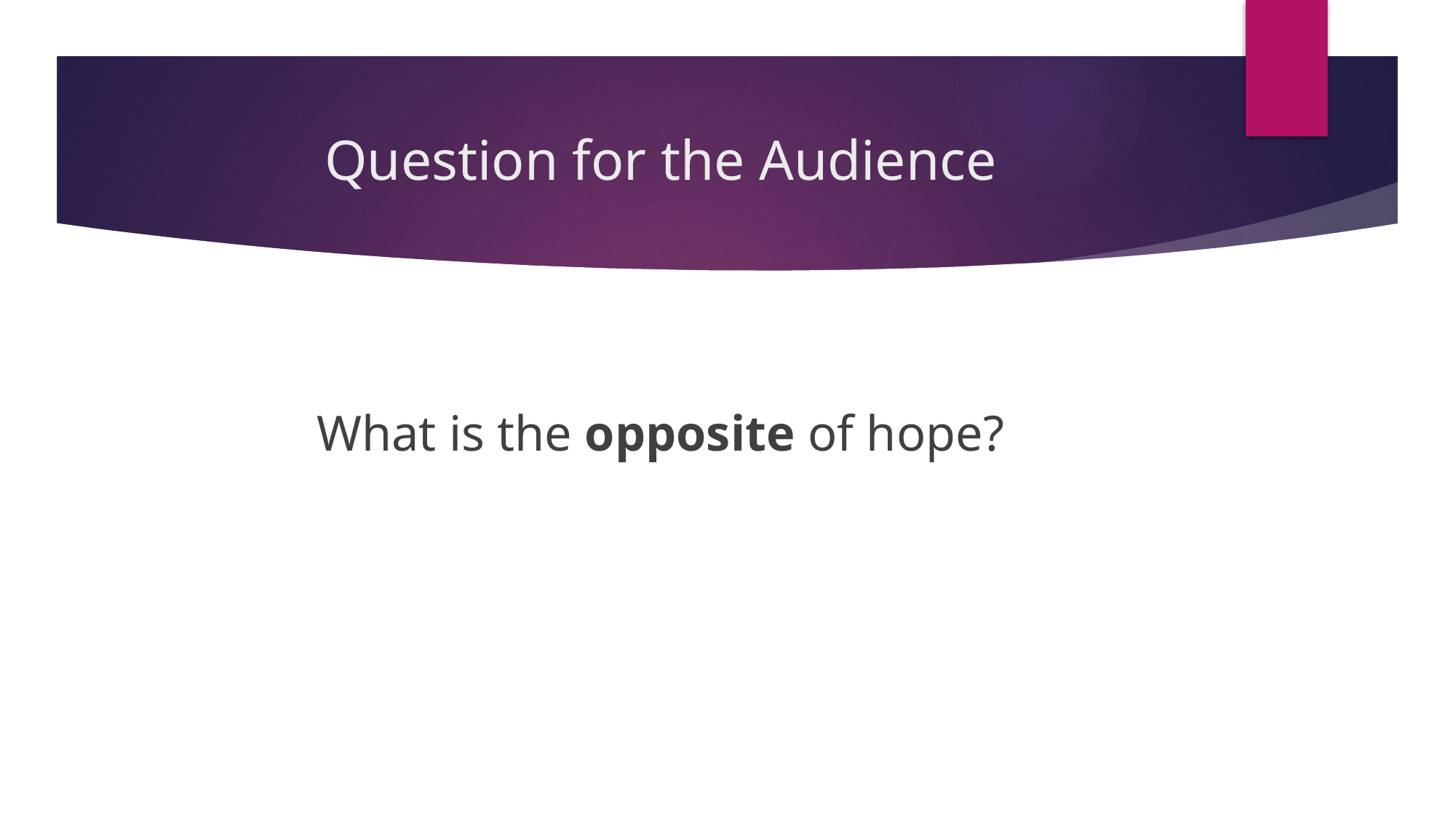

# Question for the Audience
What is the opposite of hope?

## Slide 23
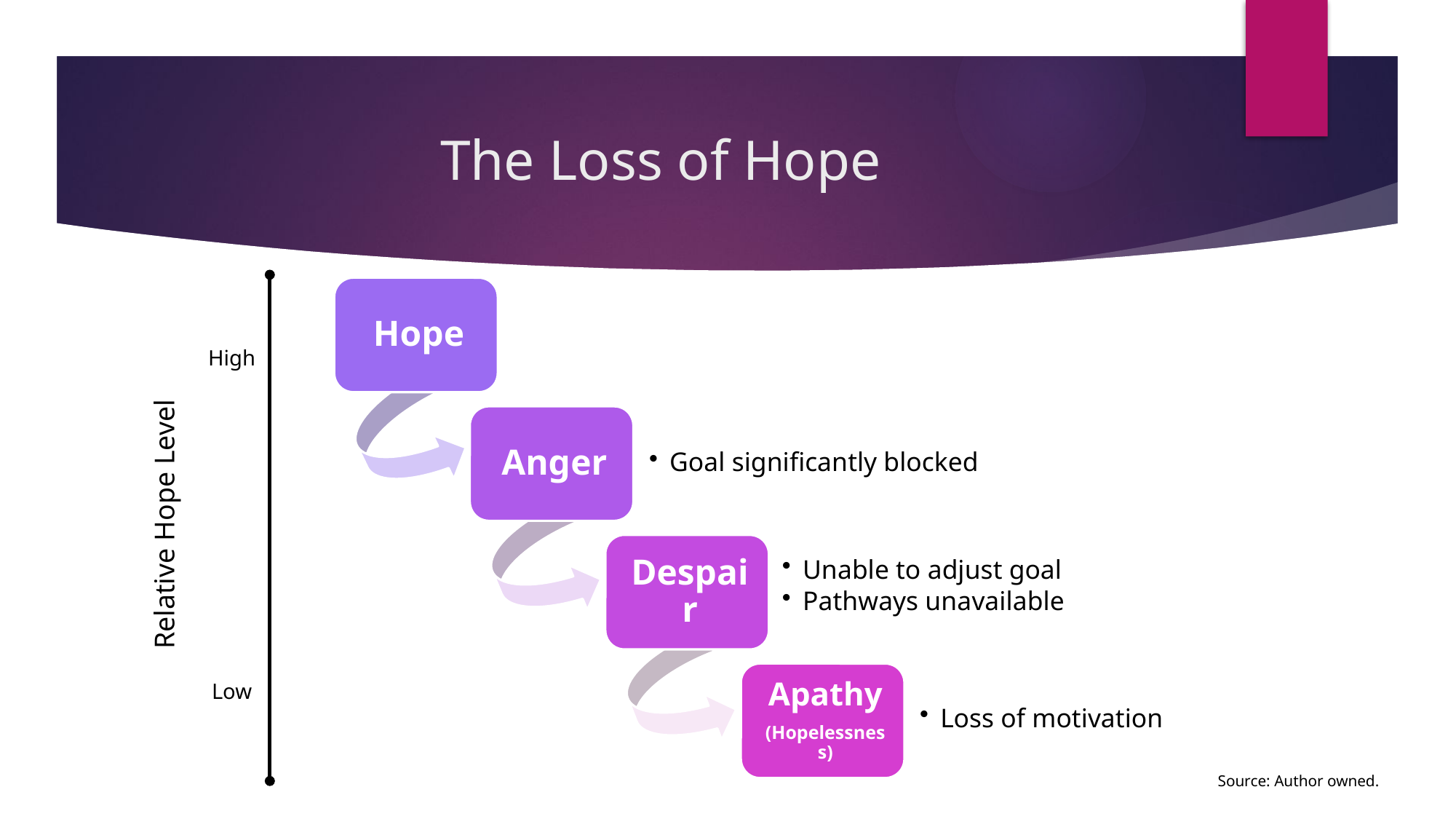

# The Loss of Hope
High
Relative Hope Level
Low
Source: Author owned.

## Slide 24
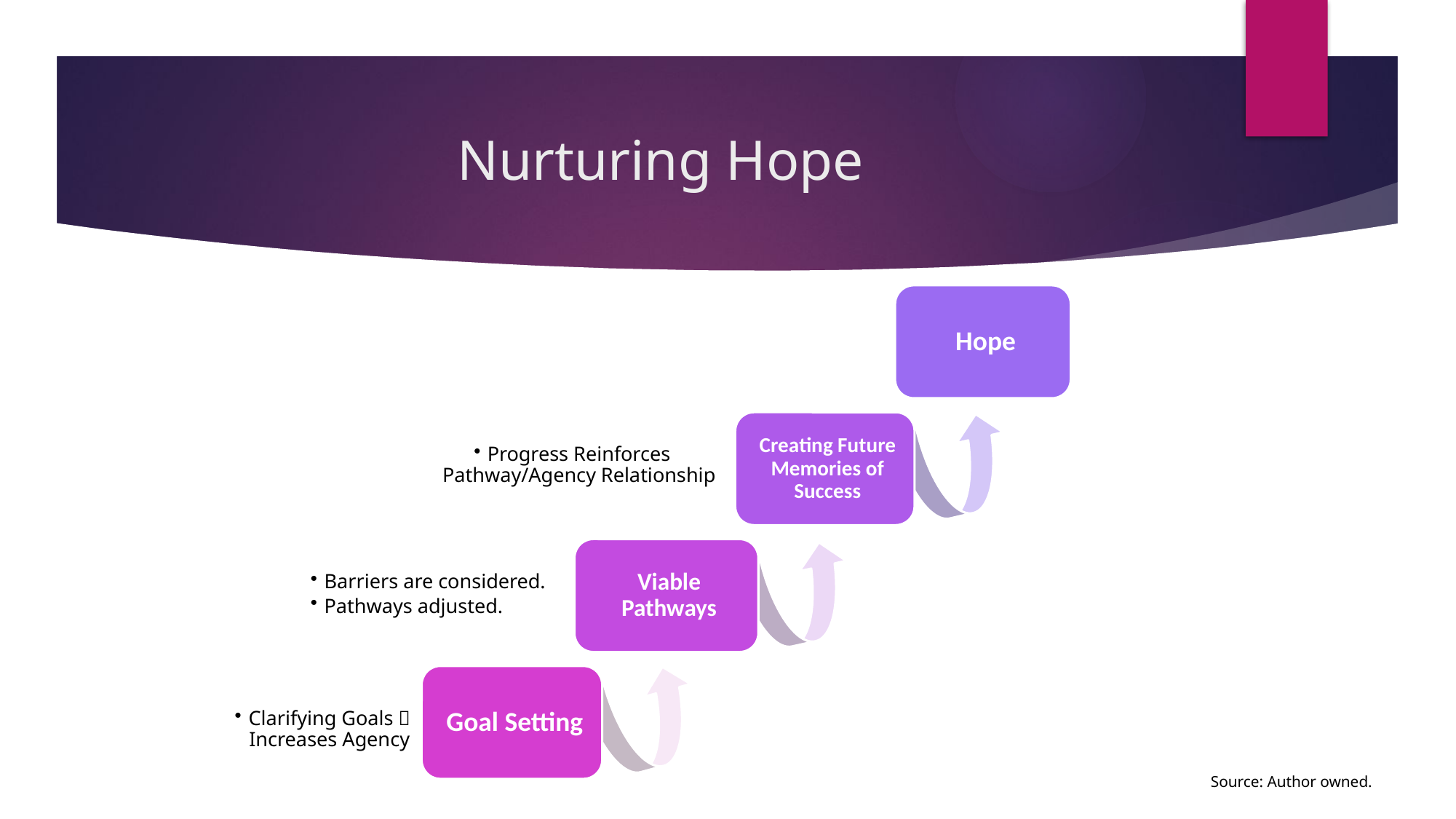

# Nurturing Hope
Source: Author owned.

## Slide 25
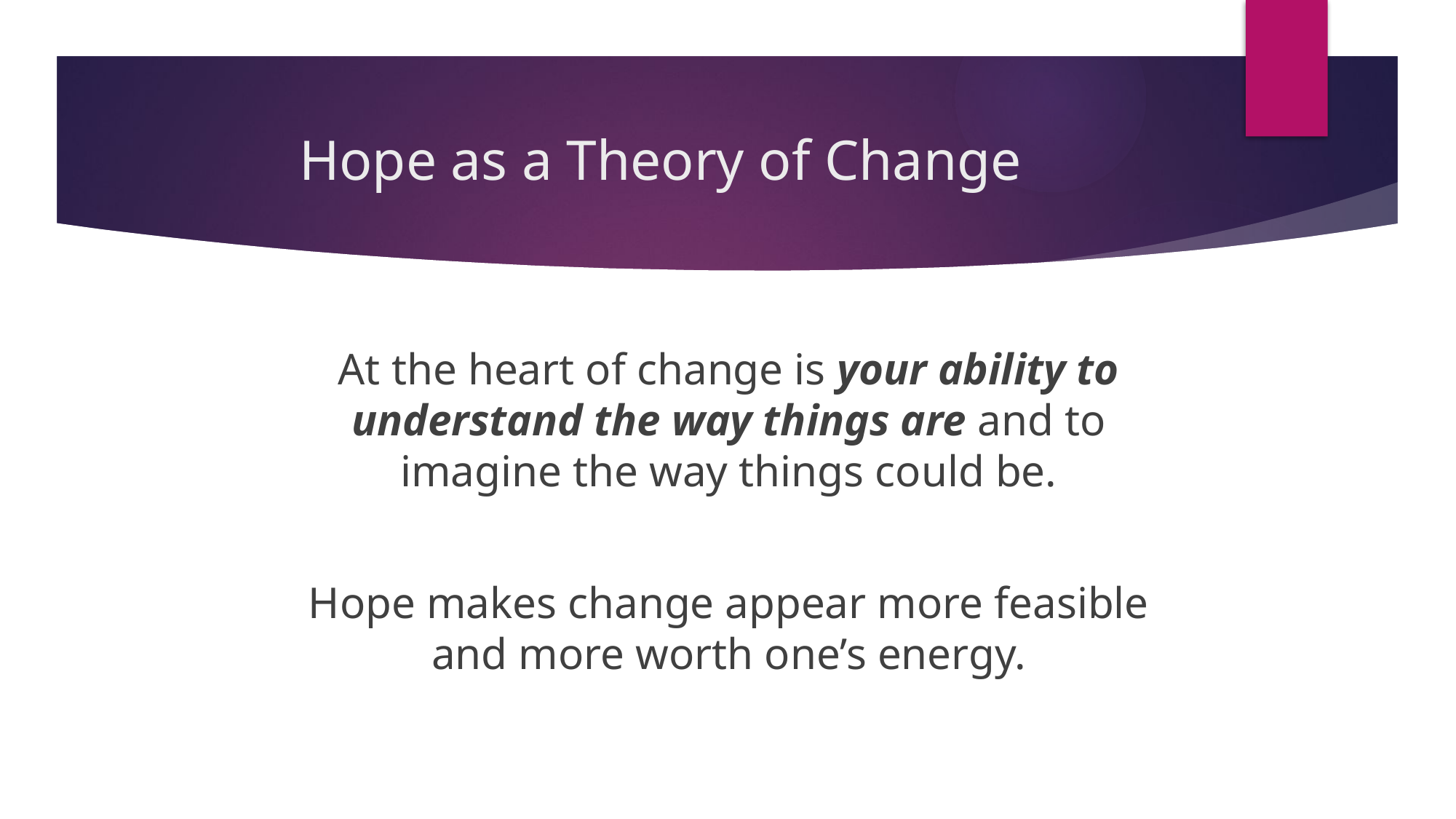

# Hope as a Theory of Change
At the heart of change is your ability to understand the way things are and to imagine the way things could be.
Hope makes change appear more feasible and more worth one’s energy.

## Slide 26
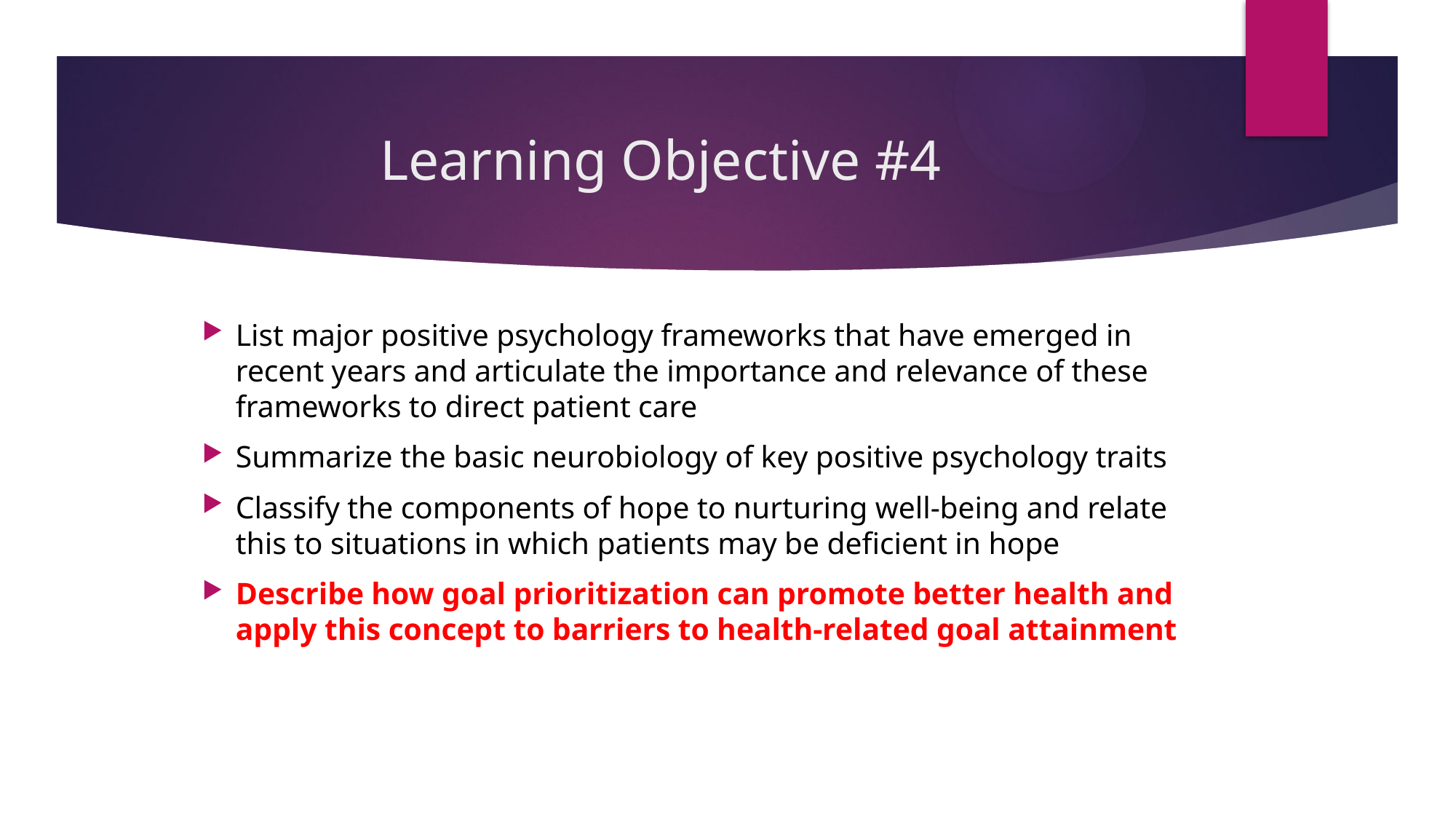

# Learning Objective #4
List major positive psychology frameworks that have emerged in recent years and articulate the importance and relevance of these frameworks to direct patient care
Summarize the basic neurobiology of key positive psychology traits
Classify the components of hope to nurturing well-being and relate this to situations in which patients may be deficient in hope
Describe how goal prioritization can promote better health and apply this concept to barriers to health-related goal attainment

## Slide 27
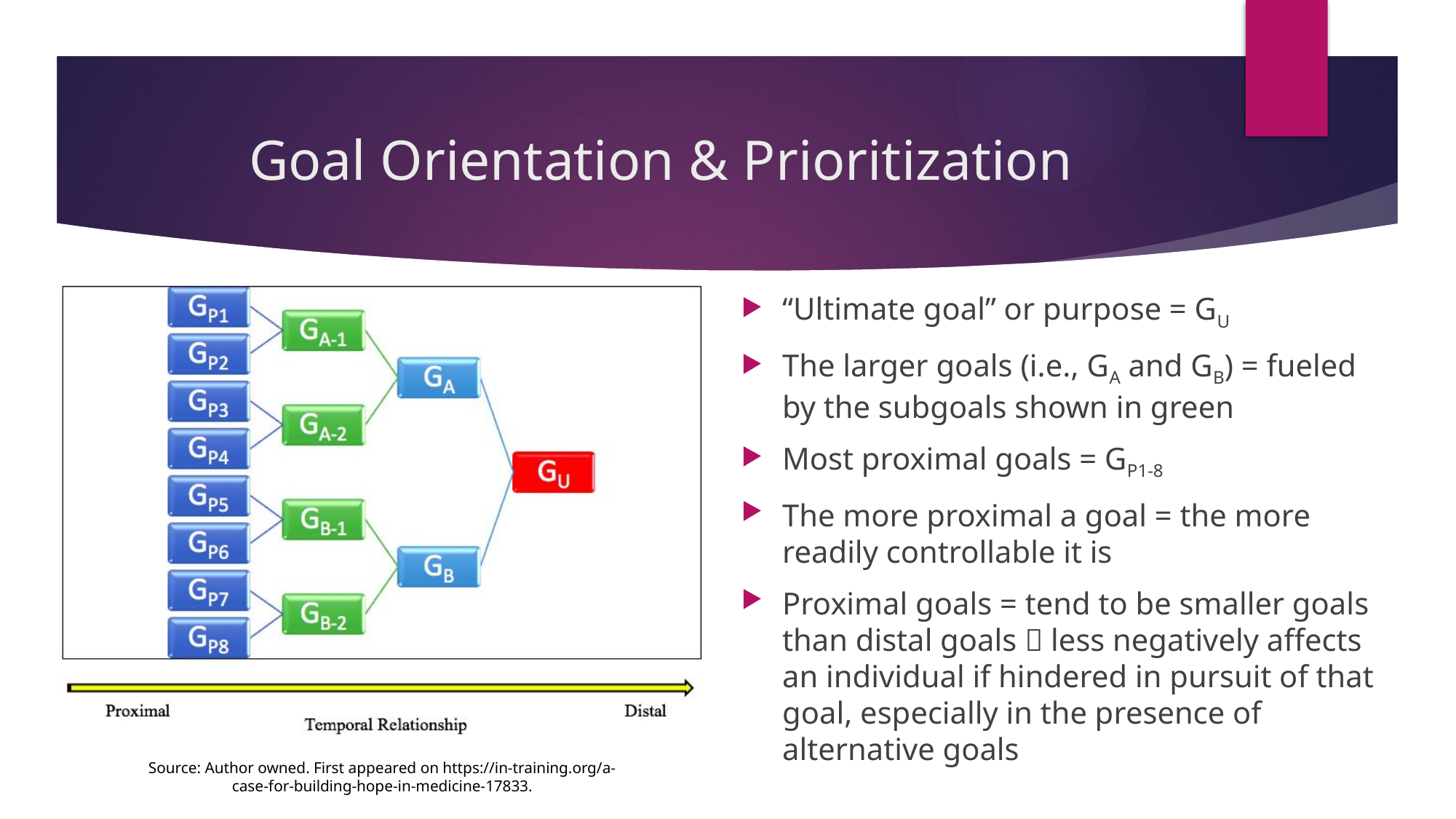

# Goal Orientation & Prioritization
“Ultimate goal” or purpose = GU
The larger goals (i.e., GA and GB) = fueled by the subgoals shown in green
Most proximal goals = GP1-8
The more proximal a goal = the more readily controllable it is
Proximal goals = tend to be smaller goals than distal goals  less negatively affects an individual if hindered in pursuit of that goal, especially in the presence of alternative goals
Source: Author owned. First appeared on https://in-training.org/a-case-for-building-hope-in-medicine-17833.

## Slide 28
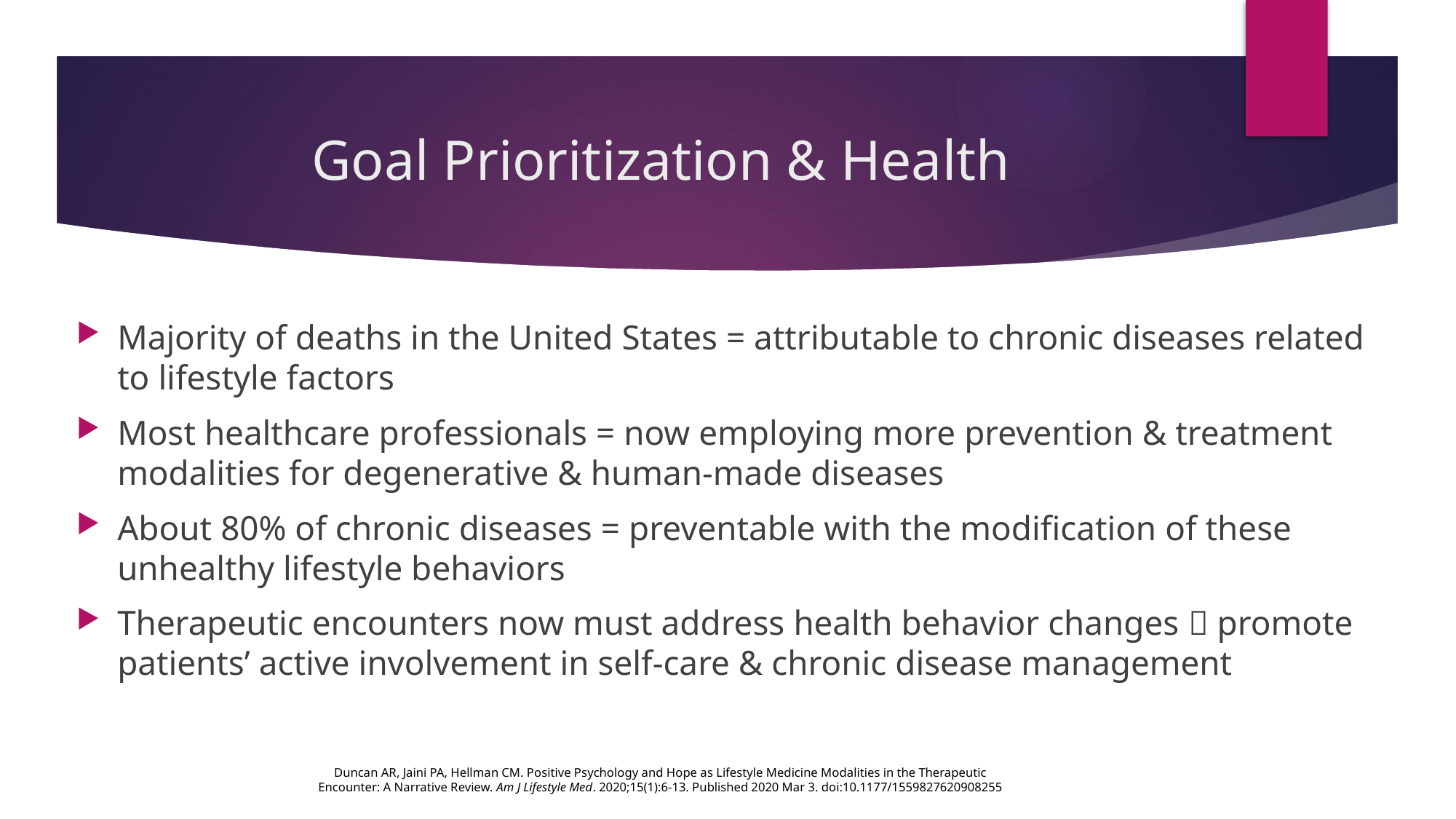

# Goal Prioritization & Health
Majority of deaths in the United States = attributable to chronic diseases related to lifestyle factors
Most healthcare professionals = now employing more prevention & treatment modalities for degenerative & human-made diseases
About 80% of chronic diseases = preventable with the modification of these unhealthy lifestyle behaviors
Therapeutic encounters now must address health behavior changes  promote patients’ active involvement in self-care & chronic disease management
Duncan AR, Jaini PA, Hellman CM. Positive Psychology and Hope as Lifestyle Medicine Modalities in the Therapeutic Encounter: A Narrative Review. Am J Lifestyle Med. 2020;15(1):6-13. Published 2020 Mar 3. doi:10.1177/1559827620908255

## Slide 29
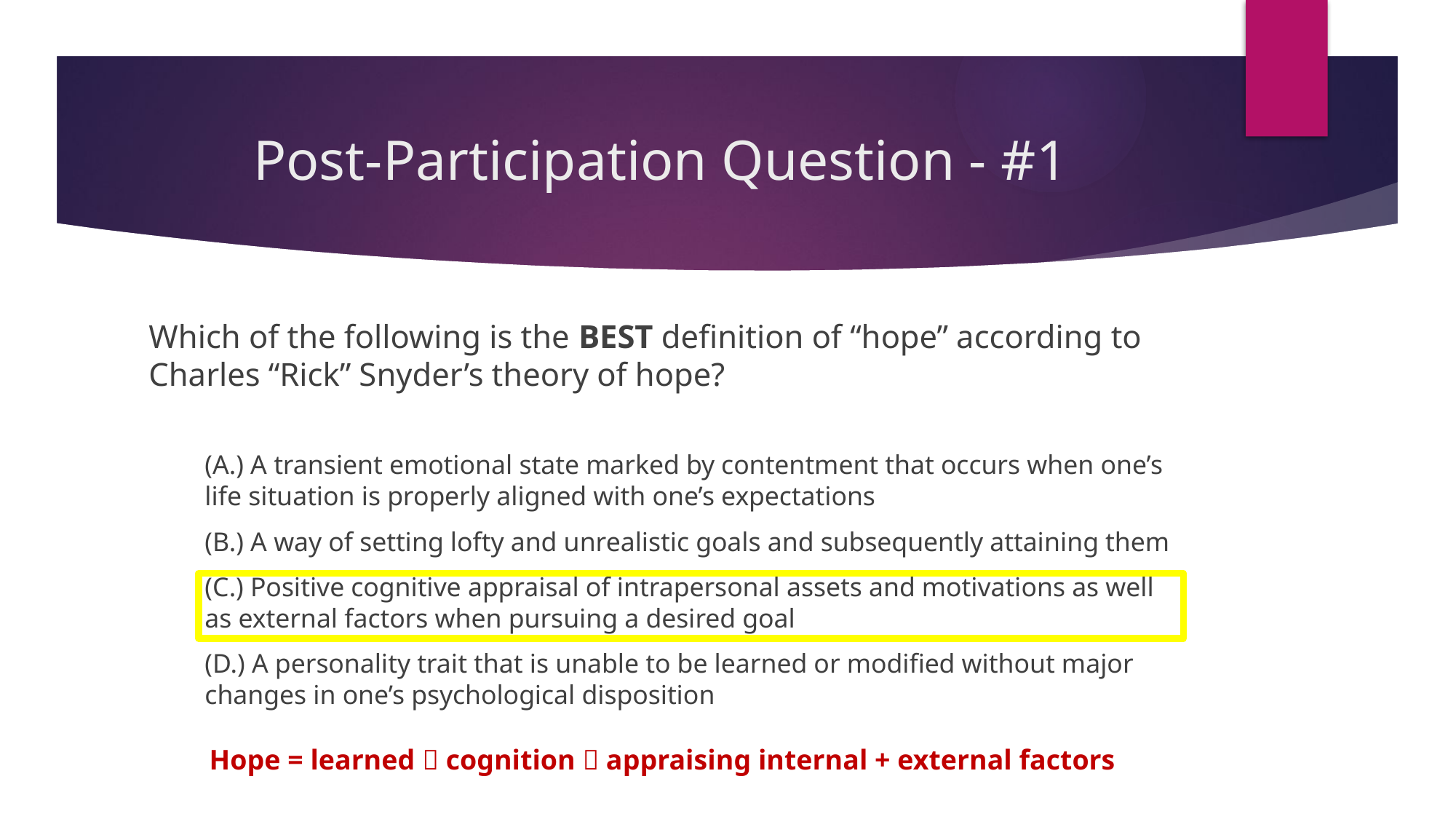

# Post-Participation Question - #1
Which of the following is the BEST definition of “hope” according to Charles “Rick” Snyder’s theory of hope?
(A.) A transient emotional state marked by contentment that occurs when one’s life situation is properly aligned with one’s expectations
(B.) A way of setting lofty and unrealistic goals and subsequently attaining them
(C.) Positive cognitive appraisal of intrapersonal assets and motivations as well as external factors when pursuing a desired goal
(D.) A personality trait that is unable to be learned or modified without major changes in one’s psychological disposition
Hope = learned  cognition  appraising internal + external factors

## Slide 30
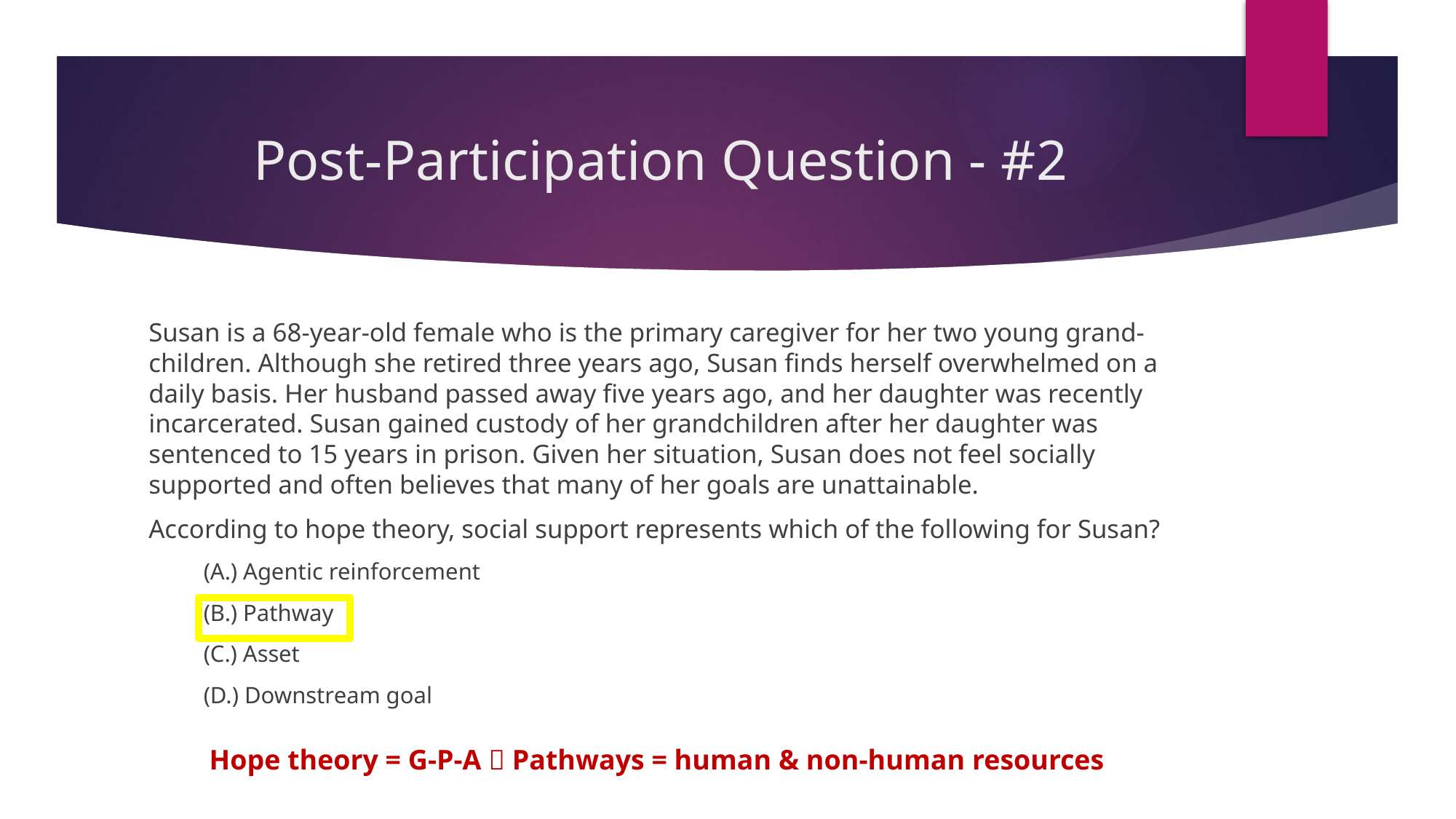

# Post-Participation Question - #2
Susan is a 68-year-old female who is the primary caregiver for her two young grand-children. Although she retired three years ago, Susan finds herself overwhelmed on a daily basis. Her husband passed away five years ago, and her daughter was recently incarcerated. Susan gained custody of her grandchildren after her daughter was sentenced to 15 years in prison. Given her situation, Susan does not feel socially supported and often believes that many of her goals are unattainable.
According to hope theory, social support represents which of the following for Susan?
(A.) Agentic reinforcement
(B.) Pathway
(C.) Asset
(D.) Downstream goal
Hope theory = G-P-A  Pathways = human & non-human resources

## Slide 31
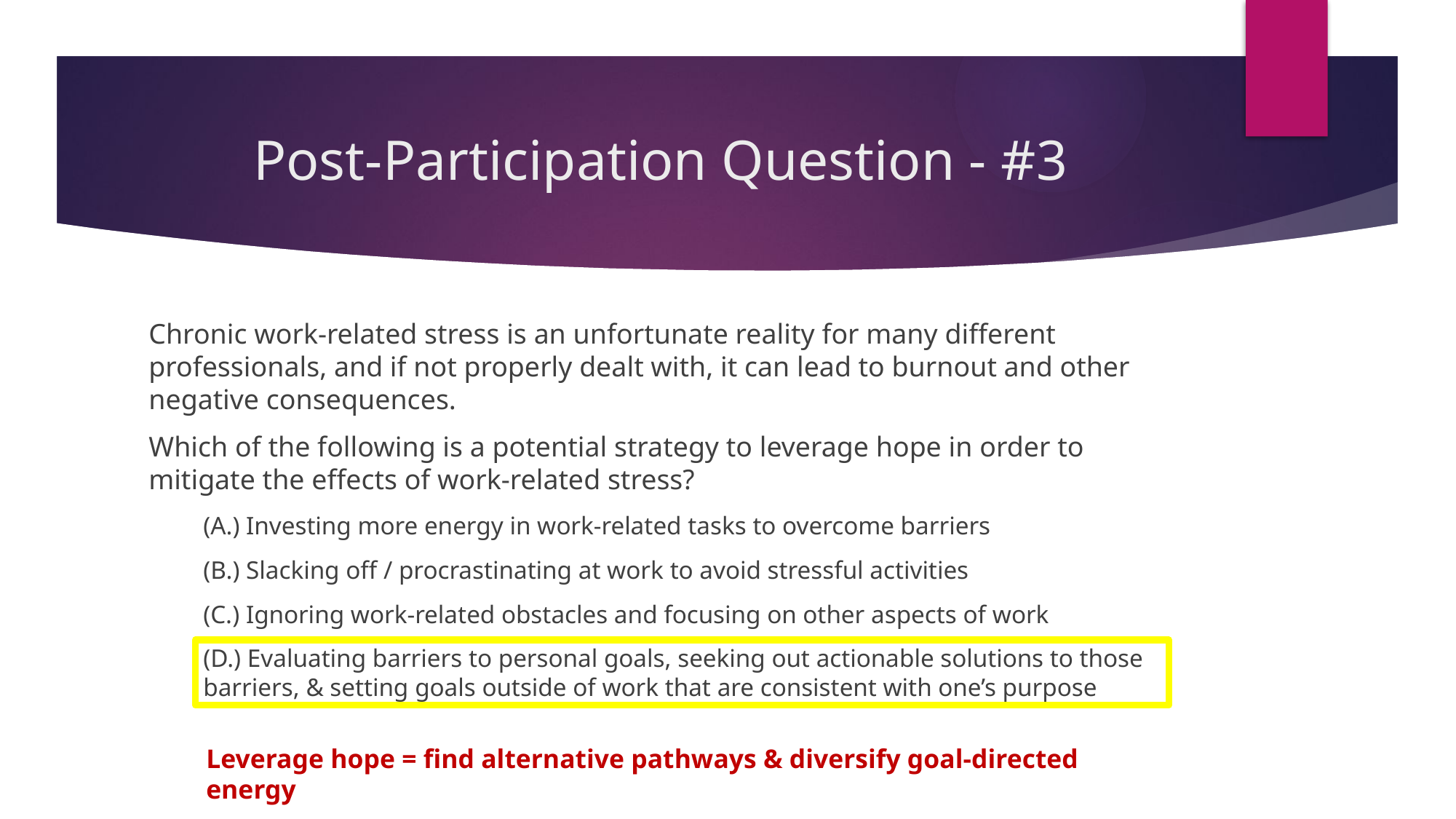

# Post-Participation Question - #3
Chronic work-related stress is an unfortunate reality for many different professionals, and if not properly dealt with, it can lead to burnout and other negative consequences.
Which of the following is a potential strategy to leverage hope in order to mitigate the effects of work-related stress?
(A.) Investing more energy in work-related tasks to overcome barriers
(B.) Slacking off / procrastinating at work to avoid stressful activities
(C.) Ignoring work-related obstacles and focusing on other aspects of work
(D.) Evaluating barriers to personal goals, seeking out actionable solutions to those barriers, & setting goals outside of work that are consistent with one’s purpose
Leverage hope = find alternative pathways & diversify goal-directed energy

## Slide 32
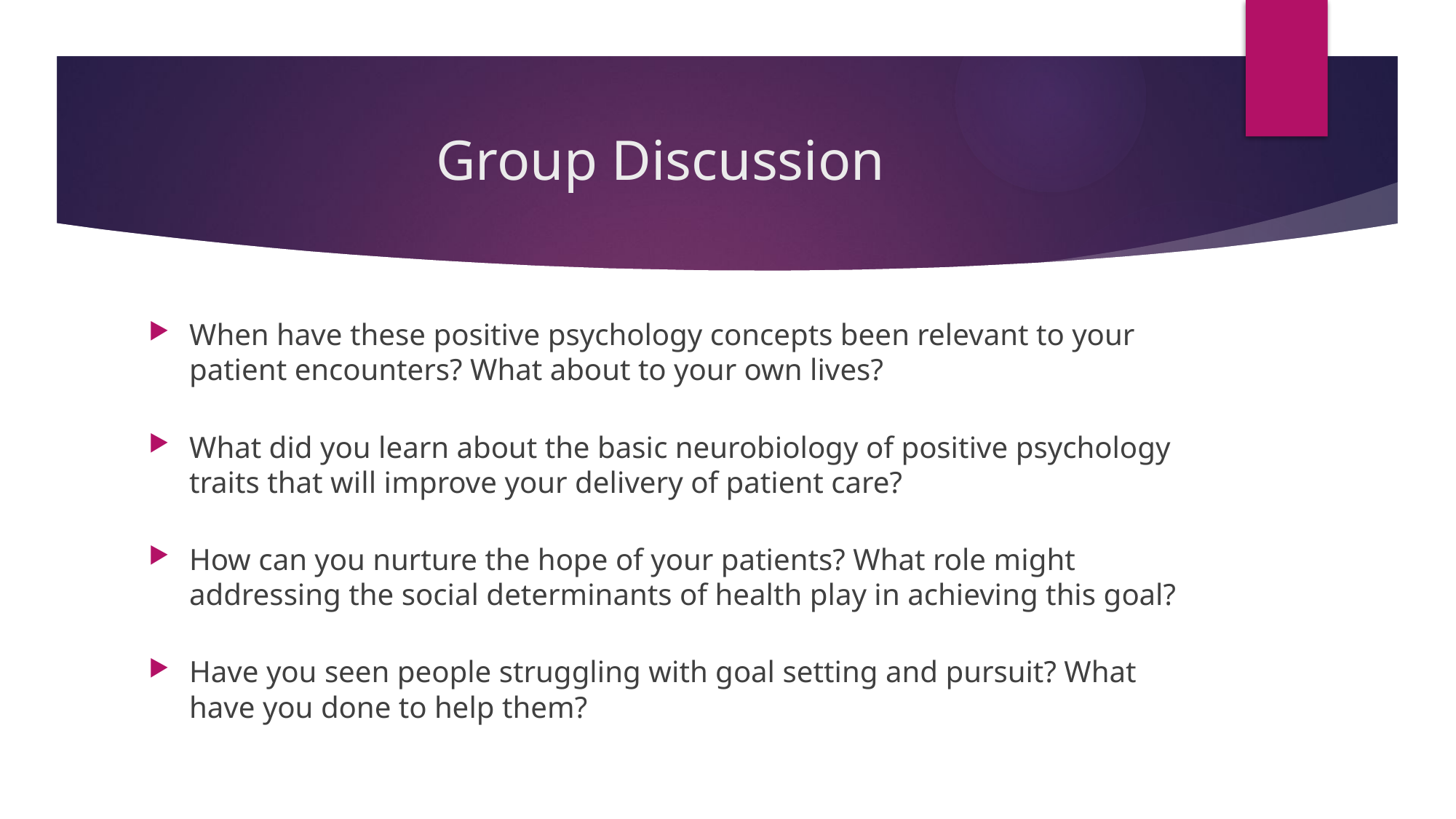

# Group Discussion
When have these positive psychology concepts been relevant to your patient encounters? What about to your own lives?
What did you learn about the basic neurobiology of positive psychology traits that will improve your delivery of patient care?
How can you nurture the hope of your patients? What role might addressing the social determinants of health play in achieving this goal?
Have you seen people struggling with goal setting and pursuit? What have you done to help them?

## Slide 33
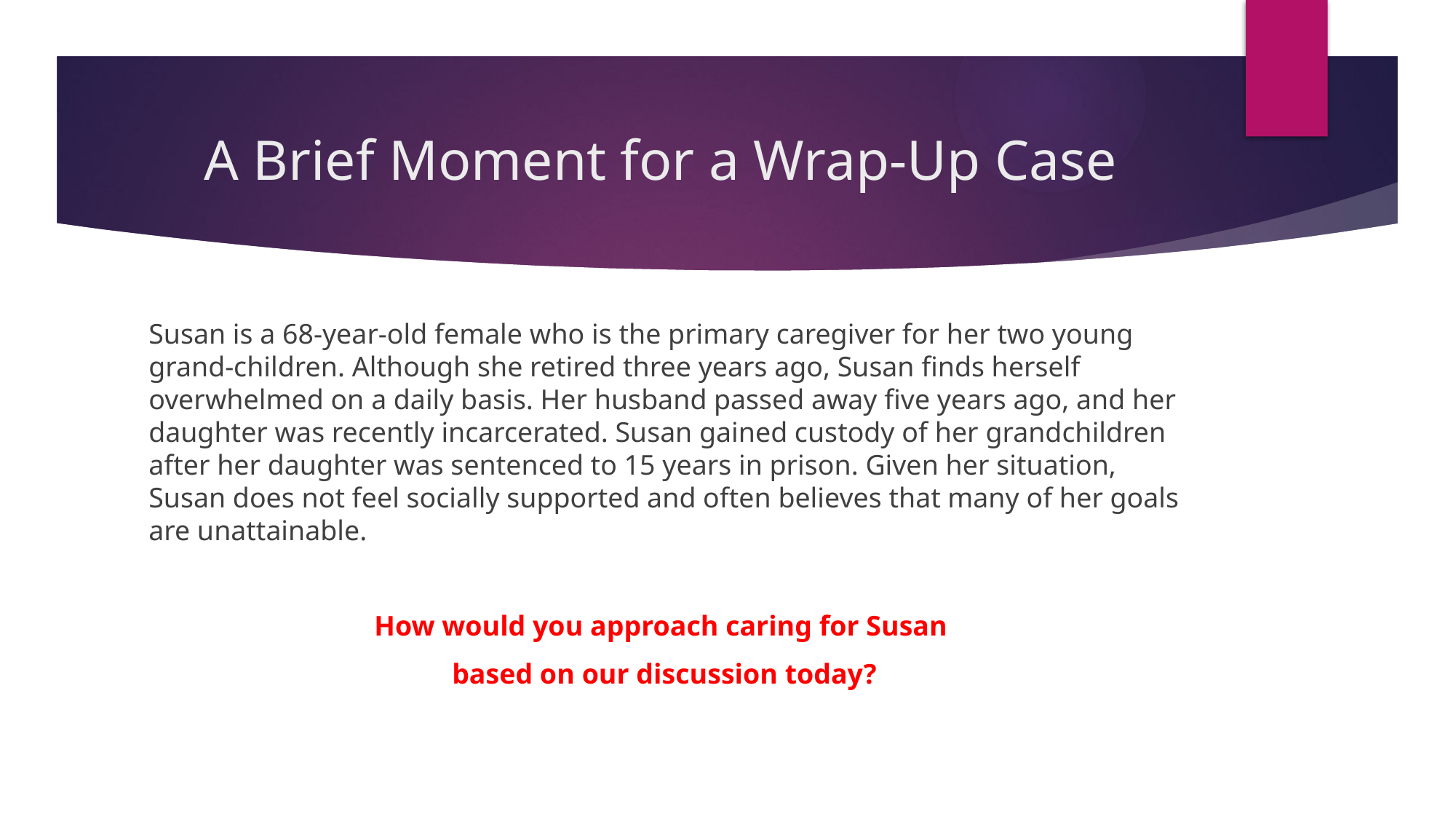

# A Brief Moment for a Wrap-Up Case
Susan is a 68-year-old female who is the primary caregiver for her two young grand-children. Although she retired three years ago, Susan finds herself overwhelmed on a daily basis. Her husband passed away five years ago, and her daughter was recently incarcerated. Susan gained custody of her grandchildren after her daughter was sentenced to 15 years in prison. Given her situation, Susan does not feel socially supported and often believes that many of her goals are unattainable.
How would you approach caring for Susan
based on our discussion today?

## Slide 34
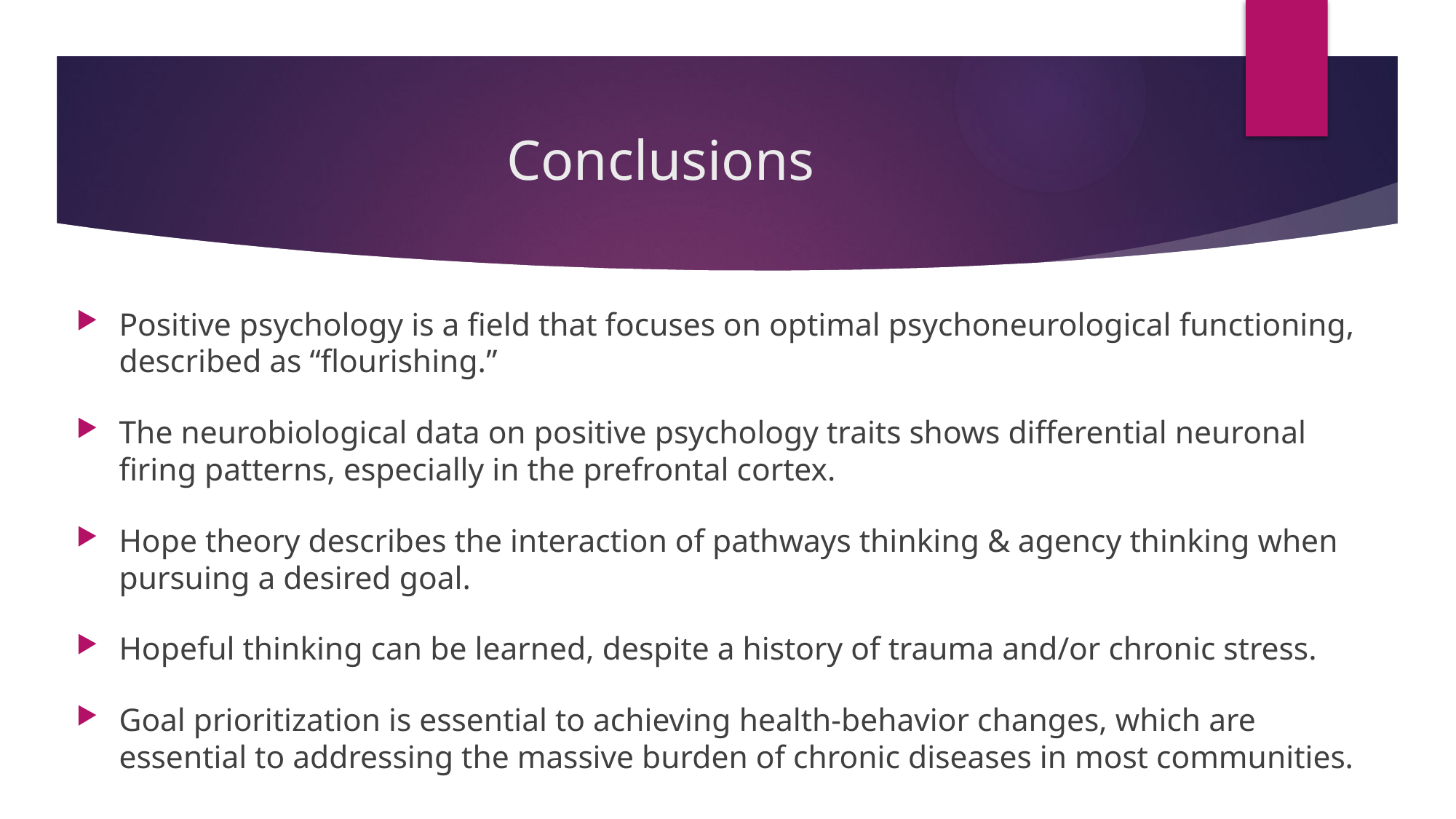

# Conclusions
Positive psychology is a field that focuses on optimal psychoneurological functioning, described as “flourishing.”
The neurobiological data on positive psychology traits shows differential neuronal firing patterns, especially in the prefrontal cortex.
Hope theory describes the interaction of pathways thinking & agency thinking when pursuing a desired goal.
Hopeful thinking can be learned, despite a history of trauma and/or chronic stress.
Goal prioritization is essential to achieving health-behavior changes, which are essential to addressing the massive burden of chronic diseases in most communities.

## Slide 35
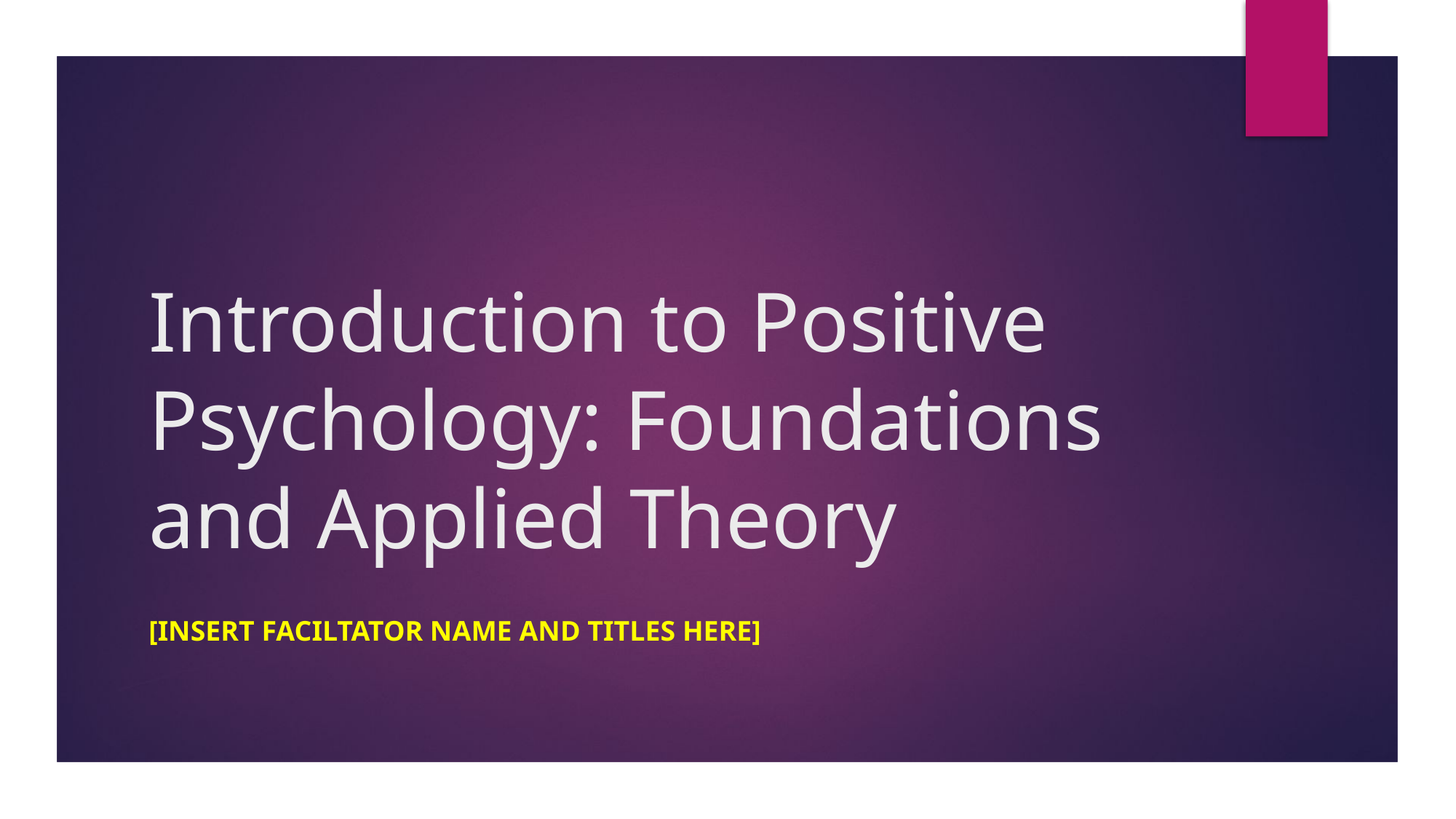

# Introduction to Positive Psychology: Foundations and Applied Theory
[Insert FACILTATOR NAME AND TITLES HERE]
