# Supplementary material for: How Positive Psychology Can Augment Leadership Through the Therapeutic Alliance
Source: MedEdPORTAL. 2025 Mar 20;21:11510. doi: 10.15766/mep_2374-8265.11510 (PMC11922800; doi:10.15766/mep_2374-8265.11510)
Supplement: Supplementary file 1 — Intro to Positive Psychology.pptxIntro to Leadership in the Therapeutic Alliance.pptxFacilitator Guide.docxSurveys.docx [file mep_2374-8265.11510-s001.zip › C. Facilitator Guide.docx]

**Facilitator’s Guide for Introduction to Positive Psychology: Foundations and Applied Theory & Introduction to Leadership in the Therapeutic Alliance Educational Modules**

Developed by

Ashten Duncan, MD, MPH

*July 2024*

Facilitator’s Guide for Introduction to Positive Psychology: Foundations and Applied Theory & Introduction to Leadership in the Therapeutic Alliance Educational Modules

**Background**

For several decades, the majority of disease, disability, and death in the United States has been attributable to chronic diseases that are directly linked to preventable lifestyle factors.^1^ Reflecting this reality, the United States Department of Health and Human Services (HHS) has outlined several leading health indicators tied to modifiable health behaviors in Healthy People 2030.^2^ As a result of this health paradigm shift, modern healthcare requires physicians and other healthcare professionals who are adept at applying psychology and leadership principles to direct patient care to help patients achieve health-behavior changes and other health-related goals.^3-5^ However, this content is not currently emphasized in many undergraduate and graduate medical curricula, and there needs to be clear, standardized content to educate medical trainees, including medical students and resident physicians. Consequently, physicians outside of behavioral healthcare settings do not routinely apply these concepts to their care of patients.^6^

In the late 20^th^ century, positive psychology emerged as a field that distinguished itself from the rest of psychology and psychiatry by focusing on the cognitive, affective, and behavioral dimensions of optimal human functioning, which refers to people being able to achieve their goals and potentials in the absence of adversity.^7^ Major psychological states and traits identified include frameworks like hope,^8^ flow,^9^ and many others connected to psychosocial flourishing, which occurs when people can focus on their purposes in life rather than individual challenges..^10^ Functional MRI and other neurobiological studies have demonstrated how the brain works differently when self-reported levels of positive psychological domains like hope are high,^11,12^ and this difference in the way humans behave and the way their brains work has major implications for executive function.^13^ For these reasons, it has been theorized that positive psychology has a direct and critical role to play in promoting health behavior changes in patients.^3^

Most undergraduate and graduate medical curricula acknowledge how indispensable strong leadership is to high-quality patient care and teamwork, particularly in topics like motivational interviewing^14^ and bidirectional performance feedback.^15^ Underpinning this emphasis is the well-accepted theory that adequate leadership practices lead to better outcomes for an organization,^16^ which can include better health outcomes for a healthcare organization. More recent research on interpersonal leadership has also demonstrated how impactful skills and qualities like emotional intelligence,^17^ psychological safety,^18^ and specific adaptive leadership styles^19^ are to achieving optimal organizational performance. For these reasons, a deep understanding of leadership concepts has the potential to improve direct patient care, especially when coupled with major underlying theories of positive psychology.

**Resource Description**

This educational resource consists of two interactive workshops lasting one hour each. Two slide decks have been created to deliver the content. Facilitators only needed a conference room, projector, screen, and computer to implement the workshops. The first workshop concerns foundational topics and applied theory in positive psychology, and the second centers on interpersonal leadership in direct patient care and the therapeutic alliance. After receiving the content, learners will be able to apply these topics to direct patient care and understand the foundations of optimal goal-setting environments.

**Purpose, Goals, and Objectives**

The purpose of this content is to synthesize foundation concepts and theories in positive psychology with applied leadership concepts to provide a roadmap for helping patients navigate crucial health behavior changes.

The following are the specific learning objectives for this content:

Introduction to Positive Psychology: Foundations and Applied Theory

1. List major positive psychology frameworks that have emerged in recent years and articulate the importance and relevance of these frameworks to direct patient care
2. Summarize the basic neurobiology of key positive psychology traits
3. Classify the components of hope to nurturing well-being and relate this to situations in which patients may be deficient in hope
4. Describe how goal prioritization can promote better health and apply this concept to barriers to health-related goal attainment

Introduction to Leadership in the Therapeutic Alliance

1. Summarize key interpersonal leadership concepts and cognitive biases that influence the patient-provider dyad and articulate the importance and relevance of these topics to direct patient care
2. Describe how emotional intelligence, self-awareness, and psychological safety impact patient care and use this knowledge to navigate common patient care challenges
3. Identify the role of the physician/provider as a leader in the therapeutic alliance and apply this understanding to cases involving patient care

**Intended Audience(s)**

The workshops are intended for medical students and resident physicians from any medical or surgical specialty with little or no knowledge, experience, or skill in applying positive psychology and interpersonal leadership to direct patient care. They are also intended as a refresher for those who already have knowledge and experience in these areas. This is an introductory program—the workshops focus on the **basics** of positive psychology and interpersonal leadership in healthcare settings.

**Prerequisites**

There are no specific prerequisites for these workshops. However, before receiving the content, it is recommended that learners be familiar with the basic principles of motivational interviewing and shared decision-making and have some exposure to direct patient care.

**Instructor Qualifications and Responsibilities**

Instructors should ideally be experienced in clinical medicine and/or direct patient care (e.g., healthcare professionals) to facilitate discussions among the learners about how the topics apply to the provision of healthcare services. Additional training in psychology and leadership is preferred but optional. Beyond this, there are no requirements for special expertise or training. The instructor should review the slide decks ahead of time to understand all topics being introduced to the learners. Background information on the topics can be obtained from the sources listed at the end of this guide. The instructor should be skilled at facilitating group discussions and allow ample time for the learners to share their ideas and questions at the times designated in the slide decks.

**Required Resources**

- Facilitator’s guide
- Projector and screen
- “Introduction to Positive Psychology: Foundations and Applied Theory” Slide Deck
- “Introduction to Leadership in the Therapeutic Alliance” Slide Deck
- Conference room
- Participants’ smartphones (recommended)

**Suggested Agenda and Timeline**

**Introduction to Positive Psychology: Foundations and Applied Theory (Total duration = 1 hour)**

- Pre-participation survey: 3 minutes
- Slides 1- 13: Introduction, Learning Objectives, Pre-Participation Questions, and First Learning Objective Content: 12-15 minutes
- Slides 14-16: Second Learning Objective Content: 5 minutes
- Slides 17-25: Third Learning Objective Content: 10 minutes
- Slides 26-28: Fourth Learning Objective Content: 5 minutes
- Slides 29-31: Post-Participation Questions: 3 minutes
- Slide 32: Group Discussion: 10-15 minutes
- Slide 33: Case Discussion: 2-3 minutes
- Slides 34-35: Conclusions and Wrap-Up: 2 minutes
  - Optional for additional time: See the extension activities below and/or spend more time on Slides 32 and 33
- Post-participation survey: 2-3 minutes (can occur during break time 🡪 only if not presenting the next presentation immediately 🡪 if so, skip to the next section)

**Introduction to Leadership in the Therapeutic Alliance (Total duration = 1 hour)**

- Pre-participation survey: 3 minutes (if not already completed)
- Slides 1- 12: Introduction, Learning Objectives, and First Learning Objective Content: 15-20 minutes
- Slides 13-18: Second Learning Objective Content: 10 minutes
- Slides 19-21: Third Learning Objective Content: 4 minutes
- Slide 22: Putting It All Together with an Activity: 10 minutes
- Slide 23-24: Audience Case: 6-7 minutes
- Slide 25: Group Discussion: 5-10 minutes
- Slides 26-27: Conclusions and Wrap-Up: 2 minutes
  - Optional for additional time: See the extension activities below and/or spend more time on Slide 25
- Post-participation survey: 2-3 minutes

**Procedures for Implementation**

To deliver this educational content, set up the slide decks and project the slides onto a screen for the group to see. For both workshops, the slides’ content will address each learning objective. There are transition slides to remind everyone which learning objective is being addressed at any given time.

In the first slide deck, the workshop starts with pre-participation questions that will be revisited at the end. Have the learners record their answers without discussion in the beginning and then have them discuss their answers at the end. Following these questions, there will be audience engagement questions, group discussion questions, and interactive cases requiring the application of the information covered in the presentation. Allow at least 1 minute of discussion for each question to optimize each learning opportunity. Verbalize each conclusion point toward the end of the presentation to make sure every learner has a firm understanding of the key takeaways.

The approach in the second slide deck will be mostly the same, except that there will be no pre-participation questions that will be revisited later in the presentation. Also, there will be an emotional intelligence assessment on slide #22, which learners can access by scanning the QR code on the screen. Otherwise, there are no other major considerations for this slide deck.

**Slide Instructions**

**Introduction to Positive Psychology: Foundations and Applied Theory**

- Slide 1: Title Slide
  - Add name(s) and background information of facilitator(s) to this slide.
  - Facilitators should introduce themselves to the audience and discuss their roles in their institutions.
  - Facilitators should indicate that they are following the content and guidelines of this module.
  - State the name of this presentation on the slide.
- Slide 2: Disclosures / Conflicts of Interest
  - Read the statements on the slide aloud.
  - Facilitators should make any relevant disclosures at this time.
- Slide 3: Learning Objectives
  - Review the learning objectives with the audience:
    - List major positive psychology frameworks that have emerged in recent years
    - Summarize the basic neurobiology of key positive psychology traits
    - Classify the components of hope to nurturing well-being
    - Describe how goal prioritization can promote better health
- Slide 4: Pre-Participation Question - #1
  - Read the question and all of the answer choices out loud.
  - Tell the audience to make a quick mental or physical note of their answers.
- Slide 5: Pre-Participation Question - #2
  - Read the question and all of the answer choices out loud.
  - Remind the audience to make a quick mental or physical note of their answers.
- Slide 6: Pre-Participation Question - #3
  - Read the question and all of the answer choices out loud.
  - Remind the audience to make a quick mental or physical note of their answers.
- Slide 7: Learning Objective #1
  - Read the learning objective in red out loud.
- Slide 8: Brief History of Positive Psychology
  - Read the text on the slide.
  - Here are recommended comments to make for each bullet point:
    - At its core, positive psychology is about what the brain and mind are doing under the best of conditions. It is about optimal functioning, which refers to what humans do when they are free of adversity and able to realize their individual potentials in their physical and social environments.
    - Some of these strengths and virtues include prudence, perseverance, gratitude, and leadership. We will discuss these more later in the presentation.
    - As a result of this research, we now have evidence of the validity of measuring these constructs across cultures, which lends credence to the idea that these topics apply to humans in general.
- Slide 9: Seligman’s PERMA Model
  - This slide contains an author-created diagram of the PERMA model, which stands for positive emotions, engagement, relationships, meaning, and accomplishments. According to Martin Seligman, these are the requirements for human flourishing, which occurs when a person is not actively facing adversity and can focus on self-actualization.
  - Facilitators can mention that these are several pillars of positive psychology and note that these pillars are as important to our well-being as they are to the well-being of our patients.
  - The next slide will go into much more detail about how to explore these domains with patients.
- Slide 10: Seligman’s PERMA Model (Cont.)
  - Review the elements of PERMA again briefly based on the first column of the table.
  - Read a few example questions out loud to the group to underscore the content.
  - Facilitators can make personal comments about how these questions can spur conversations that allow patients to discuss their lives, desires, and goals about their health.
- Slide 11: Framework of Hope Theory
  - Facilitators should start the discussion by mentioning that the figure is a diagram of hope theory, which is a framework created by the late Rick Snyder from the University of Kansas. This is the most commonly used hope framework in positive psychology and is a parsimonious behavior change theory.
  - The bolded text on this slide contains the most important points to make verbatim.
  - The sub-bullet points give more information about goals, pathways, and agency thinking, respectively. They can be paraphrased or read aloud verbatim.
  - Facilitators should refer back to the diagram when discussing these three key components of the hope theory framework to make connections between the image and words.
- Slide 12: Summary of Flow
  - The diagram on this slide summarizes flow, a positive mental state of being entirely focused on your work in which your ability to meet its demands is perfectly balanced by the skills you possess.
  - This is often captured in the common vernacular by describing someone as being “in the zone.”
  - The concept of flow came from the work of Hungarian-American psychologist Mihály Csíkszentmihályi in the 1970s.
  - Facilitators should emphasize the graph’s axes, which show the relative challenge of a task on the Y-axis and one’s skill level on the X-axis.
  - People tend to enter flow states when challenge and skill are appropriately matched.
  - The dashed lines are meant to show the boundaries of the flow state, beyond which we experience one of the outcomes stated on the graph (e.g., boredom, anxiety, etc.).
- Slide 13: Other Major Positive Psychology Frameworks
  - This is a summary slide of major concepts in positive psychology, which was briefly introduced in Slide 8.
  - Facilitators do not need to read each point out loud; mentioning a few is sufficient to underscore the field’s vastness.
  - Facilitators should mention emotional intelligence, which entails self-awareness, self-management, social awareness, and relationship management. This concept will show up again in the next slide deck.
- Slide 14: Learning Objective #2
  - Read the learning objective in red out loud.
- Slide 15: Basic Neurobiology of Key Positive Psychology Traits
  - Due to the complexity of the summarized findings on this slide, facilitators should stick to what is written and read everything aloud verbatim.
  - For the first bullet point, the facilitator should reiterate the definition of flourishing, which happens when humans are not preoccupied with obstacles and are focused on their psychological and social well-being and what they need to do to achieve their potentials.
  - For the second bullet point, facilitators can differentiate dispositional (or trait) and state hope by mentioning that dispositions are enduring patterns within an individual and that states are transient measurements of a psychological phenomenon at a single point in time. The important part is that the audience understands that states may or may not reflect what is happening over time.
  - After going through each bullet point, facilitators should emphasize that while the whole brain is involved in these phenomena, one particular area of activity increases markedly.
  - This will be explored in depth in the next slide.
- Slide 16: The Role of the Prefrontal Cortex
  - Facilitators should start by highlighting the two key areas of the prefrontal cortex: the dorsolateral and orbitofrontal areas.
  - The dorsolateral area is responsible for planning, working memory, task switching, and response inhibition.
  - The orbitofrontal area is responsible for processing rewards and punishments, integrating sensory information to guide decision-making based on expected outcomes, and regulating social behavior by inhibiting inappropriate responses.
  - Together, these prefrontal cortex areas result in “executive function,” which is emphasized on the slide.
  - The role of the prefrontal cortex in executive function should be emphasized and then connected to the bold and underlined bullet point at the bottom of the slide (i.e., goal setting).
  - Facilitators can comment on how a more active prefrontal cortex can lead to better decision-making and the ability to make long-term plans.
- Slide 17: Learning Objective #3
  - Read the learning objective in red out loud.
- Slide 18: Questions for the Audience
  - Read each question and give the audience about 1-2 minutes to respond with their reflections.
  - While there are no right or wrong answers to these questions, facilitators should expect to hear descriptions of future orientation, looking forward, expecting a better tomorrow, or something that keeps you grounded during moments of hardship. It is also entirely acceptable for the audience to echo definitions from Snyder’s hope theory, specifically around pursuing a goal
  - The most important part of this exercise is showing that hope, while a shared phenomenon among humans, arises from widely different goals, values, attitudes, and beliefs.
  - Facilitators can make that point after attendees give a few answers.
- Slide 19: What is Hope?
  - This slide recaps the points explored in depth in Slide 11. Therefore, facilitators can keep all comments brief.
  - Facilitators can point out the slide’s color coding, which connects the terms important to hope theory to the model everyone looked at in Slide 11.
- Slide 20: Hope Theory is Parsimonious
  - The text on this slide reinforces the definition of hope in Rick Snyder’s hope theory.
  - Facilitators do not need to explain this in great detail since the audience just saw it.
  - Facilitators should instead focus their time on the hope theory diagram, which is similar to the one shown in Slide 11 but now contains markers representing barriers.
  - The main point to articulate in this slide is that barriers to desired goals require people to overcome those barriers, set new goals of similar value to them, and/or abandon their goals.
- Slide 21: Summary of Hope Theory
  - Facilitators should read this slide verbatim as it highlights deeper understandings of hope and emphasizes nuanced points of view about the model, such as the reciprocal interaction between pathways and agency thinking.
  - Moreover, this slide gives the exact answer to one of the pre-participation questions.
- Slide 22: Question for the Audience
  - Similar to Slide 18, facilitators should give the audience about 1-2 minutes to offer single words or phrases to answer the question about the opposite of hope.
  - The best answer to this is “apathy,” which is the loss of goal-directed energy due to repeated or insurmountable barriers.
  - Some people may answer “depression,” “despair,” or “anger,” but these still imply some energy remains.
  - If someone answers “hopelessness,” facilitators should prompt the audience to devise a synonym for this to see if they can come up with “apathy.”
- Slide 23: The Loss of Hope
  - Facilitators should start by answering the previous question and pointing this out on the diagram.
  - Facilitators should then start from hope and go down each level to show how people lose hope, reading each accompanying text on the right-hand side.
  - There should be a final point: when hope is completely exhausted, people give up on goals. If this happens repeatedly, this can become a learned behavior and expectation.
- Slide 24: Nurturing Hope
  - Facilitators should start this discussion at the bottom with goal setting and go up level by level, reading the accompanying text on the left-hand side.
  - This is the opposite of the sequence shown in Slide 23.
  - The most crucial point to emphasize in this slide is that new memories must be created to show people they have control over their lives and can produce meaningful change with their mental energy.
  - This can be very difficult, especially since humans have a tendency toward negativity bias that reinforces negative experiences far more strongly than positive ones in our memories.
- Slide 25: Hope as a Theory of Change
  - Now that the audience has learned much about hope and hope theory, facilitators can quickly read the text on the slide verbatim.
  - Facilitators should stress the title of this slide since that is the main takeaway.
- Slide 26: Learning Objective #4
  - Read the learning objective in red out loud.
  - This is the final learning objective for the presentation.
- Slide 27: Goal Orientation & Prioritization
  - It is recommended that facilitators follow the text on the slide and start by addressing the yellow arrow at the bottom of the figure, which shows time.
  - After this, facilitators should define one’s ultimate goal in the figure and comment on how it guides goal setting for smaller and more immediate tasks like buying gas or going to a grocery store.
  - Facilitators should then discuss how larger goals influence proximal goal-setting, which creates a flow of energy from now to sometime in the undefined future toward the ultimate goal or purpose.
  - The main takeaway from this slide is that goals and goal-setting are very complex and necessary aspects of human beings, and this is largely why shared decision-making in health-behavior change conversations is so critical.
- Slide 28: Goal Prioritization & Health
  - Facilitators should read the text on the slide verbatim, adding personal comments as appropriate based on their experience with these points.
  - Facilitators should then refer back to Slide 27 and emphasize the importance of understanding where people are putting their energy in their daily lives relative to their goals. This can help clinicians identify opportunities to incorporate health-benefiting goals that are congruent with patients’ pre-established goals based on their values.
- Slide 29: Post-Participation Question - #1
  - This slide and the next two slides are all identical in content to what was shown in the pre-participation question slides.
  - The main difference is that when the facilitator advances the slide, a yellow box will appear to show the correct answer, and then red text will show the explanation.
  - Facilitators should give the audience only about 15 seconds to say their answers out loud before showing the correct one.
- Slide 30: Post-Participation Question - #2
  - Follow the instructions for Slide 29.
- Slide 31: Post-Participation Question - #3
  - Follow the instructions for Slide 29.
- Slide 32: Group Discussion
  - Depending on the amount of time remaining, facilitators should take about 10-15 minutes to read some of these questions out loud and ask the audience to answer them.
  - There are no right or wrong answers; these are meant to promote self-reflection.
  - Anticipated discussion points include exploring hope theory or any of the other positive psychology frameworks introduced in the presentation, identifying specific involvement of the prefrontal cortex in positive psychology traits, associating social determinants of health like transportation, health literacy, access to healthy foods, and financial stability with hope and goal orientation, and describing barriers to goal pursuit.
  - The points above are the key teaching points, so facilitators should cover them if the participants do not bring them up themselves.
  - There will likely be more questions on this slide than can be answered in the remaining time, so facilitators should feel empowered to pose the questions to the audience for reflection after the presentation (i.e., rhetorical questions).
- Slide 33: A Brief Moment for a Wrap-Up Case
  - Facilitators should read the case out loud to the audience and then immediately ask them to answer the question in red.
  - Anticipated discussion should involve a careful discussion of how different positive psychology frameworks apply to Susan’s situation, how hope could be nurtured by identifying discrete health-related goals, and how a professional could work with Susan to prioritize her goals based on her values.
  - Facilitators can prompt the group with these concepts if this discussion does not occur.
  - This part should take about 2-3 minutes for adequate participation.
- Slide 34: Conclusions
  - Facilitators should read these points verbatim and concisely add personal comments based on experience.
- Slide 35: Concluding Title Slide
  - Add name(s) and background information of facilitator(s) to this slide.
  - Thank the audience for their participation and attention.
  - If facilitators are going to proceed with the next presentation immediately, consider giving the audience a few minutes for a break before starting the next one.

**Introduction to Leadership in the Therapeutic Alliance**

- Slide 1: Title Slide
  - Add name(s) and background information of facilitator(s) to this slide.
  - Facilitators should introduce themselves to the audience and discuss their roles in their institutions.
  - Facilitators should indicate that they are following the content and guidelines of this module.
  - State the name of this presentation on the slide.
- Slide 2: Disclosures / Conflicts of Interest
  - Read the statements on the slide aloud.
  - Facilitators should make any relevant disclosures at this time.
  - Comments can be brief if addressed in the last presentation immediately before this.
- Slide 3: Learning Objectives
  - Review the learning objectives with the audience:
    - Summarize key interpersonal leadership concepts and cognitive biases that influence the patient-provider dyad
    - Describe how emotional intelligence, self-awareness, and psychological safety impact patient care
    - Describe the role of the physician/provider as a leader in the therapeutic alliance
- Slide 4: Learning Objective #1
  - Read the learning objective in red out loud.
- Slide 5: What is Interpersonal Leadership?
  - Facilitators should read the bullet points on the slide verbatim to the audience.
  - For the second bullet point, facilitators can provide additional examples of leadership styles not shown on the slide.
  - For the third bullet point, facilitators can prompt the audience for self-reflection on when different leadership styles are necessary. Expected responses include using more advisory leadership styles when patients are more engaged in their care and more instructive styles when patients need more direction.
  - For the fourth bullet point, facilitators should note that servant leadership means that there are times when the patient should lead decision-making and when the provider should lead.
  - Facilitators should emphasize the importance of adaptability in leadership style to achieve optimal outcomes.
- Slide 6: Components of Adaptive Leadership
  - Facilitators should read the main bullet points under “Core leadership” and “Adaptive leadership” and summarize the examples on the slide.
  - The sub-bullet points provide examples of what the main bullet points mean.
  - Facilitators should emphasize that adaptive leadership also includes all of the components of core leadership in addition to the four characteristics listed in that column.
  - Facilitators should conclude the slide by stating that adaptation is the most essential aspect of adaptive leadership. This means leaders are willing to incorporate new experiences and information to improve their skills over time.
- Slide 7: Interpersonal Leadership in Healthcare
  - Facilitators should read the bullet points on the slide verbatim to the audience and can add examples of the common strategies mentioned based on personal experience.
  - For the second bullet point, facilitators should consider defining motivational interviewing as a communication style that tasks the provider with helping patients change health-related behaviors by exploring and resolving ambivalence toward change. This will be explored in much more detail later in the presentation.
  - For the third bullet point, facilitators can refer to the presentation on positive psychology—particularly the content on goal prioritization—to drive the point home and directly link the contents together.
- Slide 8: Question for the Audience
  - Read the question aloud and give the audience about 1-2 minutes to respond with their reflections.
  - The most important part of this exercise is for the audience to recognize what can jeopardize strong leadership approaches when working with patients.
  - Example responses may include providers defining goals and values for the patient, patients not actively participating in their care, power imbalances, unidirectional communication (mostly from providers to patients), and a lack of shared understanding.
  - The most preferred response is one about cognitive biases (including prejudices) because it will be discussed in much more detail in the following slide.
- Slide 9: Ubiquity of Cognitive Biases
  - Given the number of important definitions on this slide, facilitators should stick to what is written and read everything aloud verbatim.
  - For the second bullet point, facilitators can give examples of explicit and implicit biases and note that emerging research suggests that many, if not most, of our biases are implicit. Recognizing implicit biases requires interventions like implicit association tests to reveal underlying thoughts and beliefs.
  - For the third bullet point, facilitators should refer back to the first bullet point to emphasize that biases are neither entirely positive nor negative. Different biases serve different purposes, and awareness of those purposes is crucial for supporting our patients.
  - For the fourth bullet point, facilitators can refer to Daniel Kahneman’s work around availability heuristics if they would like to learn more on the subject.
- Slide 10: Types of Cognitive Biases
  - Facilitators should start by describing the examples of cognitive biases shown in the figure on the left-hand side.
  - Facilitators are encouraged to review the definitions of these biases prior to presenting, which can be easily accessed online.
  - In particular, facilitators may want to define the Dunning-Kruger effect (i.e., overestimation of one’s abilities or knowledge in a specific area due to a lack of skill or knowledge), framing effect (i.e., decision-making based on how an issue is presented rather than on the facts presented), and gambler’s fallacy (i.e., erroneous belief that a random event is more likely to occur because a series of the opposite outcomes occurred) since these may be less familiar to the audience.
  - Each bullet point should be read aloud to the audience.
  - For the second bullet point, facilitators can prompt the audience for examples of anchoring bias in their clinical work. The best response is when providers focus on only one or two diagnoses when listening to a patient rather than considering the many possibilities that explain the same symptoms or concerns.
- Slide 11: Question for the Audience
  - Read the question aloud and give the audience about 1-2 minutes to respond with their reflections.
  - Example responses to this question include decreasing the quality of care, leading to more healthcare errors, lowering patient satisfaction, and delaying accurate diagnosis and management.
  - The most important part of this exercise is for the audience to recognize that biases can negatively affect healthcare.
- Slide 12: Cognitive Biases & Interpersonal Leadership
  - Given the importance of underscoring the theoretical overlap between goal orientation and leadership practices, facilitators should stick to what is written and read everything aloud verbatim.
  - For the second bullet point, facilitators should emphasize that patients ultimately decide what their goals are and how to spend their energy pursuing different goals.
  - For the fourth bullet point, facilitators can list barriers like the social determinants of health to emphasize that blaming a patient for a suboptimal health outcome is seldom helpful and usually does not get to the root cause of the outcome.
- Slide 13: Learning Objective #2
  - Read the learning objective in red out loud.
- Slide 14: Emotional Intelligence
  - Facilitators should start by describing the emotional intelligence framework shown in the figure on the left-hand side.
  - Special attention should be paid to the four core components of emotional intelligence: self-awareness, self-management, social awareness, and relationship management.
  - Subcomponents of these four core components can be briefly summarized.
  - All of the text on the right-hand side should be read aloud verbatim.
  - Facilitators should emphasize the need to improve skills in all four domains of emotional intelligence to increase EI meaningfully and to focus on areas of weakness first.
- Slide 15: Johari Window – Building Self-Awareness
  - Facilitators should start by summarizing the figure on the left-hand side, which shows the four main domains of the Johari Window.
  - Facilitators should provide a concrete example of how the Johari Window applies to healthcare, including information that certain professionals know and others do not and how that information can be shared in a group.
  - Each bullet point on the right-hand side should be read aloud.
- Slide 16: Psychological Safety
  - Facilitators should start by summarizing the elements of a psychologically safe environment in the panel entitled “Inputs.”
  - After that, facilitators should relate these elements to favorable outcomes listed in the right panel of the figure.
  - Each bullet point on the right-hand side should be read aloud.
- Slide 17: Why Psychological Safety Matters
  - This figure summarizes key concepts from the cited text in relation to crucial conversations.
  - When someone says something, the first step is to perceive the statement’s meaning, represented by the outermost arrows on each side.
  - After this, the meaning is interpreted, leading to an emotional response and an action. The inner arrows represent this sequence.
  - The red circle shows how people will tend toward silence (i.e., suppressed response) or violence (e.g., arguing, insulting, etc.) if psychological safety is lacking.
  - The middle green circle shows how safety must be achieved before people can enter into a pool of shared meaning.
  - Facilitators should articulate all of these points when showing this slide.
- Slide 18: Question for the Audience
  - Read the question aloud and give the audience about 1-2 minutes to respond with their reflections.
  - The most important part of this exercise is for the audience to articulate how psychological safety is tied to communication and how its deficiency leads people not to share information freely.
  - Facilitators can show Slides 16 and 17 again if the audience needs help to answer this question.
- Slide 19: Learning Objective #3
  - Read the learning objective in red out loud.
- Slide 20: Therapeutic Alliance
  - Facilitators should start by giving an overview of the therapeutic alliance framework shown in the figure on the left-hand side.
  - Special attention should be paid to the four critical steps to establishing a therapeutic alliance: investing in the beginning, eliciting the patient’s perspective, demonstrating empathy, and investing in the end.
  - Subcomponents of these four steps can be briefly summarized.
  - Facilitators can provide an example of a patient encounter that exemplifies each of these steps to drive the points home to the audience.
  - All of the text on the right-hand side should be read aloud verbatim.
- Slide 21: Overlap with Motivational Interviewing (MI)
  - Facilitators should start by highlighting the four major themes of motivational interviewing: open-ended questions, affirmations, reflective listening, and summarizing.
  - Rather than reading each action verbatim, facilitators can use these to briefly show what each motivational skill looks like in patient interactions.
  - Facilitators should discuss how the therapeutic alliance framework dovetails with the motivational interviewing framework.
  - Facilitators should briefly highlight each theme in the chart and relate it to the figure shown in Slide 20.
- Slide 22: Putting It All Together with an Activity
  - This is a suggested activity from the Institute for Health and Human Potential (https://www.ihhp.com/free-eq-quiz/) to get a baseline emotional intelligence measurement for each audience member.
  - This is meant to supplement the discussions on the next slide.
  - If facilitators would prefer to skip this activity, they should mention the Institute for Health and Human Potential and that free emotional intelligence assessments are available online.
  - If the activity is completed, allow the audience about 2 minutes to discuss their results voluntarily.
  - There is a placeholder box in the slide where facilitators can add a QR code to make it easier for learners to access the quiz. Add a code, list the website URL, or delete the placeholder box altogether.
- Slide 23: Audience Case
  - Facilitators should read the case out loud to the audience.
  - This should take at most 1-2 minutes.
- Slide 24: Audience Case (Cont.)
  - Facilitators should take about 5 minutes for this section to read each question individually and allow about 1-1.5 minutes for responses.
  - Examples of correct responses to the first question are the framing effect, sunk-cost fallacy, and availability heuristic.
    - Participants should justify their answers.
  - Facilitators should connect the second question with emotional intelligence only after participants respond.
  - For the third question, participants should identify non-confrontational strategies to make communication more manageable for the patient to establish psychological safety.
  - For the last question, example responses include the following: emotional intelligence, organizational justice, character, and development.
- Slide 25: Group Discussion
  - Depending on the amount of time remaining, facilitators should take about 5-10 minutes to read some of these questions out loud and ask the audience to answer them.
  - There are no right or wrong answers; these are meant to promote self-reflection.
  - Anticipated discussion points include descriptions of specific leadership styles like servant leadership, cognitive biases like anchoring bias and framing effects in interactions with patients, concrete strategies to navigate biases with patients, self-assessment of emotional intelligence and areas for improvement, and applications of the educational content to specific patient care situations.
  - The points above are the key teaching points, so facilitators should cover them if the participants do not bring them up themselves.
  - There will likely be more questions on this slide than can be answered in the remaining time, so facilitators should feel empowered to pose the questions to the audience for reflection after the presentation (i.e., rhetorical questions).
- Slide 26: Conclusions
  - Facilitators should read these points verbatim and concisely add personal comments based on experience.
- Slide 27: Concluding Title Slide
  - Add name(s) and background information of facilitator(s) to this slide.
  - Thank the audience for their participation and attention.
  - Ask the audience members to complete their post-participation survey.

**Assessment**

To assess the effectiveness of delivering the content in this workshop, it is recommended that the facilitator(s) administer the pre- and post-participation surveys (see below in the Evaluation section).

**Evaluation**

Pre-participation, immediate post-participation, and 6- to 8-week follow-up surveys were administered to assess the effectiveness of the course material. Printable versions of these surveys are provided as Appendix D.

**Relationship to Other Educational Materials**

This module is designed to be part of a three-part educational series focusing on the foundations and applications of positive psychology and interpersonal leadership in the therapeutic alliance context. The content in this series can serve as stand-alone learning opportunities or be administered in succession. Please consult *MedEdPORTAL* for more information.

**Extension Activities**

If there is extra time during the two-hour session or if the facilitator would like to extend the activity, it is recommended to show the following clips that provide additional background on the topics covered:^20-22^

Seligman M. The New Era of Positive Psychology | TED Talk. February 2004. Accessed August 31, 2024. <https://www.ted.com/talks/martin_seligman_the_new_era_of_positive_psychology?language=en>.

Description: *Overview of the history and direction of positive psychology as a field*

Bradberry T. The Power of Emotional Intelligence | Travis Bradberry | TEDxUCIrvine. YouTube. August 7, 2017. Accessed August 31, 2024. <https://www.youtube.com/watch?v=auXNnTmhHsk>.

Description: *Deeper dive into what emotional intelligence is and how it can be developed*

Brown B. The power of vulnerability | TED Talk. June 2010. Accessed August 31, 2024. <https://www.ted.com/talks/brene_brown_the_power_of_vulnerability?language=en>.

Description: *Detailed talk about vulnerability and communication and their power in life*

**References**

1. Centers for Disease Control and Prevention. National Center for Chronic Disease Prevention and Health Promotion (NCCDPHP): chronic disease in America. Accessed August 27, 2023. https://www.cdc.gov/chronicdisease/resources/infographic/chronic-diseases.htm.
2. Department of Health and Human Services [HHS]. Leading Health Indicators - Healthy People 2030. Accessed October 21, 2023. https://health.gov/healthypeople/objectives-and-data/leading-health-indicators.
3. Duncan AR, Jaini PA, Hellman CM. Positive Psychology and Hope as Lifestyle Medicine Modalities in the Therapeutic Encounter: A Narrative Review. *Am J Lifestyle Med*. 2020;15(1):6-13. Published 2020 Mar 3. doi:10.1177/1559827620908255
4. Smith K, Bhavsar M. A new era of health leadership. *Healthc Manag Forum*. 2021;34(6):332-335. doi:10.1177/08404704211040817
5. Palmer B, Walls M, Burgess Z, Stough C. Emotional intelligence and effective leadership. *Leadersh Organ Dev J*. 2001;22(1):5-10. doi:10.1108/01437730110380174
6. Lianov LS, Barron GC, Fredrickson BL, et al. Positive psychology in health care: defining key stakeholders and their roles. *Transl Behav Med*. 2020;10(3):637-647. doi:10.1093/tbm/ibz150
7. Lee Duckworth A, Steen TA, Seligman MEP. Positive psychology in clinical practice. *Annu Rev Clin Psychol*. 2005;1(1):629-651. doi:10.1146/annurev.clinpsy.1.102803.144154
8. Snyder CR, Harris C, Anderson JR, et al. The will and the ways: development and validation of an individual-differences measure of hope. *J Pers Soc Psychol*. 1991;60:570-585. doi: 10.1037/0022-3514.60.4.570
9. Nakamura J, Csikszentmihalyi M. The Concept of Flow. In: Snyder CR, Lopez SJ, eds. *Handbook of Positive Psychology*. Oxford University Press; 2002.
10. Peterson C, Seligman ME. *Character Strengths and Virtues: A Handbook and Classification*. 1st ed. Washington: American Psychological Association; 2004.
11. Duncan AR, Daugherty G, Carmichael G. An Emerging Preventive Mental Health Care Strategy: The Neurobiological and Functional Basis of Positive Psychological Traits.*Front Psychol*. 2021;12:728797. doi:10.3389/fpsyg.2021.728797
12. Wang S, Xu X, Zhou M, et al. Hope and the brain: Trait hope mediates the protective role of medial orbitofrontal cortex spontaneous activity against anxiety. *Neuroimage*. 2017;157:439-447. doi:10.1016/j.neuroimage.2017.05.056
13. Jones DT, Graff-Radford J. Executive Dysfunction and the Prefrontal Cortex. *Continuum.* 2021;27(6):1586-1601. doi:10.1212/CON.0000000000001009
14. Arbuckle MR, Foster FP, Talley RM, Covell NH, Essock SM. Applying Motivational Interviewing Strategies to Enhance Organizational Readiness and Facilitate Implementation Efforts. *Qual Manag Health Care*. 2020;29(1):1-6. doi:10.1097/QMH.0000000000000234
15. Ramani S, Könings K, Mann KV, van der Vleuten C. Uncovering the unknown: A grounded theory study exploring the impact of self-awareness on the culture of feedback in residency education. *Med Teach*. 2017;39(10):1065-1073. doi:10.1080/0142159X.2017.1353071
16. Lamm K, Carter H, Lamm A. A theory-based model of interpersonal leadership: An integration of the literature. *J Leadersh Educ*. 2016;15(4):183-205. doi:10.12806/v15/i4/t2
17. Goleman D. *Emotional intelligence: Why it can matter more than IQ*. Bloomsbury Publishing; 1996.
18. Grailey KE, Murray E, Reader T, Brett SJ. The presence and potential impact of psychological safety in the healthcare setting: an evidence synthesis. *BMC Health Serv Res*. 2021;21(1):773. Published 2021 Aug 5. doi:10.1186/s12913-021-06740-6
19. Yahaya R, Ebrahim F. Leadership styles and organizational commitment: Literature review. *J Manag Dev*. 2016;35(2):190-216. doi:10.1108/jmd-01-2015-0004
20. Seligman M. The New Era of Positive Psychology | TED Talk. 2004. Accessed August 31, 2024. https://www.ted.com/talks/martin_seligman_the_new_era_of_positive_psychology?language=en.
21. Bradberry T. The Power of Emotional Intelligence | Travis Bradberry | TEDxUCIrvine. YouTube. 2017. Accessed August 31, 2024. https://www.youtube.com/watch?v=auXNnTmhHsk.
22. Brown B. The power of vulnerability | TED Talk. 2010. Accessed August 31, 2024. https://www.ted.com/talks/brene_brown_the_power_of_vulnerability?language=en.

**Citation**

Duncan, AR. *Facilitator’s Guide for Introduction to Positive Psychology: Foundations and Applied Theory & Introduction to Leadership in the Therapeutic Alliance Educational Module*. Santa Fe, NM: University of New Mexico-Santa Fe Family Medicine Residency Program, 2024.
